# Supplementary material for: ‘We have been in lockdown since he was born’: a mixed methods exploration of the experiences of families caring for children with intellectual disability during the COVID-19 pandemic in the UK
Source: BMJ Open. 2021 Sep 30;11(9):e049386. doi: 10.1136/bmjopen-2021-049386 (PMC8487017; doi:10.1136/bmjopen-2021-049386)
Supplement: Supplementary data [file bmjopen-2021-049386supp001.pdf]

Supplementary file

Interview schedule

1. How do you feel the coronavirus pandemic is affecting you and your family, if at all? It may be helpful to consider your response in terms of routine, appointments, respite care, support networks, visiting family, childcare arrangements, work and related thoughts and feelings.

Prompts:

a. Can you tell me a bit more about the coronavirus pandemic has changed your and your child's:
   i. Day to day life?
   ii. Childcare?
   iii. Access to services? Have any appointments been re-arranged, taken place online or been cancelled/postponed? How easy have you found accessing online/telephone appointments?
   iv. Seeing friends and family?
   v. Access to support networks?

2. How do you feel the coronavirus pandemic is affecting your ability to cope?

3. How do you feel the coronavirus pandemic is affecting your parenting skills?

4. How are you getting along as a family? / How is everyone getting along together?

5. How do you feel the physical (social) distancing and lockdown/isolation measures have affected you and your child/children during the coronavirus pandemic, if at all?
If this is affecting you, can you explain or list in what ways?
For example, consider the impact on your support network, social activities, social media and online socialising, and general contact with family, friends and services etc.

6. How do you feel that the easing of lockdown measures/restrictions are affecting you and your child/children, if at all? If this is affecting you, can you explain or list in what ways? For example, consider the impact on your support network, social activities, social media and online socialising, and general contact with family, friends and services etc.

7. In an ideal world, what resources or allowances would you need to manage lockdown/ to help your child?

8. Are there any coping strategies that you are using during this coronavirus pandemic? If so, what are these coping strategies and how have they helped you?

9. Have there been any unexpected positives during the pandemic? Is there anything from the lockdown that you would like to carry forward into everyday life for you/your child?

10. Is there anything else about your experiences during this pandemic that you would like to mention?

Wolstencroft J, *et al.* *BMJ Open* 2022; 11:e049386. doi: 10.1136/bmjopen-2021-049386

Genetic variants of participants

Variant Type	Variants
Aneuploidy	48,XXYY
Aneuploidy	45,X
CNV	chr17p11.2 Duplication
CNV	chr18q12.3 Triplication
CNV	chr3q26.33-q27.1 Deletion
Aneuploidy	48,XXYY
CNV	chr7q11.23 Deletion
CNV	chr10q22.3-q23.2 Deletion
CNV	chr5q31.3 Duplication & chrXp22.1 Deletion
CNV	chr1q21.1-q21.2 Deletion
SNV	chr20p13 Missense & chrXq25 Missense
SNV	chrXp22.12 Splice site
CNV	chr16p13.11-p12.3 Duplication & chr7p21.1 Deletion
SNV	chr2q24.3 Missense
CNV	chr5q14.3 Deletion
SNV	chrXq26.1 Missense
CNV	chr16p13.11 Deletion
CNV	chr13q33.2-q33.3 Deletion
CNV	chr18p11.32-p11.21 & chrXp22.12 Duplication
CNV	chr7p21.1 Deletion
CNV	chr15q11.1-q13.1 Mosaic duplication
CNV	chr16p13.11-p12.3 Duplication
CNV	chr16p13.11 Deletion

Table S1: Genetic variants of IMAGINE-ID children with IDD

Wolstencroft J, *et al. BMJ Open* 2022; 11:e049386. doi: 10.1136/bmjopen-2021-049386
