# Supplementary material for: ‘We have been in lockdown since he was born’: a mixed methods exploration of the experiences of families caring for children with intellectual disability during the COVID-19 pandemic in the UK
Source: BMJ Open. 2021 Sep 30;11(9):e049386. doi: 10.1136/bmjopen-2021-049386 (PMC8487017; doi:10.1136/bmjopen-2021-049386)
Supplement: Author's manuscript [file bmjopen-2021-049386.draft_revisions.pdf]

BMJ Open

BMJ Open is committed to open peer review. As part of this commitment we make the peer review history of every article we publish publicly available.

When an article is published we post the peer reviewers' comments and the authors' responses online. We also post the versions of the paper that were used during peer review. These are the versions that the peer review comments apply to.

The versions of the paper that follow are the versions that were submitted during the peer review process. They are not the versions of record or the final published versions. They should not be cited or distributed as the published version of this manuscript.

BMJ Open is an open access journal and the full, final, typeset and author-corrected version of record of the manuscript is available on our site with no access controls, subscription charges or pay-per-view fees (http://bmjopen.bmj.com).

If you have any questions on BMJ Open's open peer review process please email info.bmjopen@bmj.com

BMJ Open

"We've been in lockdown since he was born": Experiences of families caring for children with intellectual disability during the Covid-19 pandemic

Journal:	*BMJ Open*
Manuscript ID	bmjopen-2021-049386
Article Type:	Original research
Date Submitted by the Author:	26-Jan-2021
Complete List of Authors:	Wolstencroft, Jeanne; University College London<br>Hull, Laura; University College London<br>Warner, Lauren; University College London<br>Akhtar, Tooba Nadeem; University College London<br>Mandy, W; University College London<br>IMAGINE, ID<br>Skuse, David ; University College London, Institute of Child Health
Keywords:	COVID-19, MENTAL HEALTH, PAEDIATRICS, PSYCHIATRY

SCHOLARONE™
Manuscripts

I, the Submitting Author has the right to grant and does grant on behalf of all authors of the Work (as defined in the below author licence), an exclusive licence and/or a non-exclusive licence for contributions from authors who are: i) UK Crown employees; ii) where BMJ has agreed a CC-BY licence shall apply, and/or iii) in accordance with the terms applicable for US Federal Government officers or employees acting as part of their official duties; on a worldwide, perpetual, irrevocable, royalty-free basis to BMJ Publishing Group Ltd ("BMJ") its licensees and where the relevant Journal is co-owned by BMJ to the co-owners of the Journal, to publish the Work in this journal and any other BMJ products and to exploit all rights, as set out in our licence.

The Submitting Author accepts and understands that any supply made under these terms is made by BMJ to the Submitting Author unless you are acting as an employee on behalf of your employer or a postgraduate student of an affiliated institution which is paying any applicable article publishing charge ("APC") for Open Access articles. Where the Submitting Author wishes to make the Work available on an Open Access basis (and intends to pay the relevant APC), the terms of reuse of such Open Access shall be governed by a Creative Commons licence – details of these licences and which Creative Commons licence will apply to this Work are set out in our licence referred to above.

Other than as permitted in any relevant BMJ Author's Self Archiving Policies, I confirm this Work has not been accepted for publication elsewhere, is not being considered for publication elsewhere and does not duplicate material already published. I confirm all authors consent to publication of this Work and authorise the granting of this licence.

For peer review only

"We've been in lockdown since he was born": Experiences of families caring for children with intellectual disability during the Covid-19 pandemic

Name of Authors
Jeanne Wolstencroft
Laura Hull
Lauren Warner
Tooba Akhtar
William Mandy
IMAGINE-ID consortium
David Skuse

Affiliations
Jeanne Wolstencroft (corresponding author), The Great Ormond Street Institute of Child Health, University College London, 30 Guilford Street, London, WC1N 1EH

Laura Hull, Division of Psychology and Language Sciences, Faculty of Brain Sciences, University College London, 26 Bedford Way, London, WC1H 0AP

Lauren Warner, The Great Ormond Street Institute of Child Health, University College London, 30 Guilford Street, London, WC1N 1EH

Tooba Akhtar, The Great Ormond Street Institute of Child Health, University College London, 30 Guilford Street, London, WC1N 1EH

William Mandy, Division of Psychology and Language Sciences, Faculty of Brain Sciences, University College London, 26 Bedford Way, London, WC1H 0AP

IMAGINE-ID consortium (see Imagine ID Consortium Membership list)

David Skuse, The Great Ormond Street Institute of Child Health, University College London, 30 Guilford Street, London, WC1N 1EH

Corresponding address and email
Jeanne Wolstencroft (corresponding author)
j.wolstencroft@ucl.ac.uk
The Great Ormond Street Institute of Child Health
University College London
30 Guilford Street
London, WC1N 1EH
0207 905 2168

For peer review only
For peer review only - http://bmjopen.bmj.com/site/about/guidelines.xhtml

Abstract

Objectives: This study aimed to explore the experiences of parents caring for children with intellectual and developmental disability during the UK national lockdown in spring 2020, resulting from the Covid-19 pandemic. Design: Participants were identified using opportunity sampling from the IMAGINE-ID national (UK) cohort, and completed an online survey followed by a semi-structured interview. Interviews were analysed using thematic analysis. Setting: Interviews were conducted over the telephone in July 2020 as the first UK lockdown was ending. Participants: 23 mothers of children with intellectual and developmental disabilities aged 5 to 15 were recruited. Results: Themes reported by parents included: managing pre-existing challenges during a time of extreme change, having mixed emotions about the benefits and difficulties that arose during the lockdown, and the need for appropriate, individualised support. Conclusions: Observations raised by parents suggested recommendations for policy in the event of future pandemic restrictions, namely: empowering parents as experts, providing tailored digital intervention, and supporting parents' mental health to support children.

Article Summary

Strengths and limitations of this study:

- This was the first known study to explore the experiences of parents who were caring for children with intellectual and developmental disabilities during the first UK national lockdown.
- 23 caregivers were interviewed in July 2020 as the first UK lockdown was ending
- Only mothers were interviewed and so results may not reflect the experiences of other caregivers of children with intellectual and developmental disabilities

Keywords

Intellectual disability, developmental disabilities, covid-19, pandemics, pandemics, mental health

For peer review only

Introduction

Intellectual and developmental disabilities (IDD) affect approximately 1.8% of children worldwide [1].They are associated with significant limitations in cognitive and adaptive skills [2]. Genetic variation can be identified in up to 60% of IDD cases [3]. These changes can be large chromosomal abnormalities (such as aneuploidies and translocations), sub-microscopic deletions or duplications (such as copy number variants; CNV) or small changes in DNA sequence (such as single nucleotide variants; SNV).

Children with IDD are at increased risk of behavioural difficulties compared to typically developing children [4–9]. Families of a child with IDD are more likely to live in deprived socio-economic circumstances [10–14], and experience increased parenting stress or parental psychological distress [15–21] than typically developing children.

The IMAGINE-ID consortium has recruited children with IDD of identified genetic aetiology from across the UK. The coronavirus (Covid-19) pandemic poses unique challenges to this cohort in two respects. Firstly, children with IDD have high levels of pre-existing health conditions and so may be considered especially vulnerable to the virus. As a result, families of these children are more likely than the general population to have been shielding, following UK government advice. Secondly, children with IDD are more likely to make use of services (such as specialist education, healthcare, and social care) which were suspended or diminished during the pandemic. Because of these challenges, families of children with IDD may have been especially impacted by the Covid-19 pandemic. Little is known about the impact of the pandemic on children's mental health and behavioural adjustment, although calls have been made to prioritise the collection of high-quality data on the mental health effects of the pandemic, particularly within vulnerable groups [22,23].

There has been some early examination of the impact of Covid-19 within the broader areas of developmental disorders (specifically autism), and intellectual disability. Interviews and surveys of caregivers have revealed that support and services for children with IDD and/or autism dramatically reduced, or stopped altogether during the pandemic [24,25]. Schools and local authorities in particular have not provided sufficient educational support for autistic children [26].

Surveys with adults and their caregivers suggest that where online services have been provided, these often require extensive adaptation to the individual's needs, which is not always possible [27,28]. Adults with IDD in particular reported finding it hard to understand and follow the frequently changing guidelines and restrictions [29,30].

Another consequence of the pandemic reported so far is the high levels of stress and anxiety experienced by caregivers [23,31–33]. However, some also noted the use of telemedicine, and families spending more time together, as unexpected positive consequences [33,24].

Recommendations have been developed from these and from expert opinion pieces, for the best ways to support people with IDD, and those who support them. Keeping schools and medical services open for children/adults who require particular support was suggested, as these individuals should be considered a priority for access to services [28,35]. Furthermore, concerns have been raised about how these individuals can adapt to changes in rules during de-confinement and possible future lockdowns, and special allowances and/or communications have been proposed [35,25].

To our knowledge there has not yet been any research specifically examining the experiences of caregivers of children with IDD, who have been caring for their children at home during the pandemic. These caregivers can give important insights into the impact of Covid-19 on their children, as well as on the family as a whole, and so provide empirical guidance for how best to support these families as the pandemic continues.

For peer review only - http://bmjopen.bmj.com/site/about/guidelines.xhtml

Aims and objectives

We sought to understand the experiences of families caring for a child with IDD during the Covid-19 pandemic in order to identify key targets for support. We interviewed 23 IMAGINE-ID families mid-July 2020. At this time, the lockdown restrictions were eased in parts of the UK, but the government advised vulnerable individuals to continue to shield until the 1st of August 2020. This meant that families were able to reflect on their experiences of the first national lockdown.

Our objectives were: (1) to identify the areas of difficulty and resilience during the pandemic and de-confinement; (2) to understand more about the experiences of this cohort in order to identify key targets for support; and (3) to provide recommendations for transitions in and out of periods of confinement.

Methods

Study Design

The IMAGINE-ID programme of research, funded by the UK Medical Research Council (MRC) from 2014 to 2024, recruited a cohort of 3,402 UK families whose child had IDD due to an identified genetic anomaly (www.imagine-id.org). IMAGINE-ID caregivers with children under the age of 16 were invited to take part through the study newsletter. Parents were enrolled in the current study on a first come, first served basis. A mixed-methods approach was used in order to capture the diversity of challenges encountered and resilience factors drawn upon by families. Data were collected by parent report using online questionnaires and a semi-structured telephone interview (n=23). Questionnaire data were collected and managed using REDCap electronic data capture tools hosted at University College London [37]. Telephone interviews were recorded and transcribed by the research team. Ethical approval for the study was obtained from UCL Research Ethics Committee (9553/003), and all participants consented to take part and for their interviews to be recorded.

Patient and Public Involvement

The research objectives were developed in response to conversations with collaborators at the UNIQUE rare chromosome disorder charity. A summary of the results will be disseminated to participants and the IMAGINE-ID cohort via the study newsletter, website and social media accounts.

Measures

Coronavirus Impact: An adapted version of the National Institute of Health CoRonavIruS Health Impact Survey (CRISIS) was used to assess the family's adaptations to the pandemic, including key domains relevant to mental distress and resilience [38].

Child Characteristics: Demographic details and genetic data about the child were extracted from the IMAGINE-ID database. The Wessex Scales were used to gain an updated measure of adaptive function and degree of IDD [39]. The measure assesses self-help skills, literacy, mobility and incontinence.

Wellbeing: The wellbeing of the child and the parent were assessed using the Strengths and Difficulties Questionnaire (SDQ) and the Hospital Anxiety and Depression scale (HADS) respectively The SDQ is a well-validated behavioural screening questionnaire for children and has been validated with the parents of children with disabilities [40]. The SDQ includes scales that measure emotional symptoms, conduct problems, hyperactivity/impulsivity and inattention difficulties, peer relationship problems and prosocial behaviour. The first four scales are combined to create a total difficulties score. On the total difficulties scale scores of 17-19 are indicative of "high" levels of behavioural difficulty and 20-40 are considered "very high" levels of behavioural difficulty compared to population norms. An additional impact scale measures the impact of this composite score on daily life. Scores of 3-10 on the impact scale are in the "very high" range compared to population samples.

For peer review only - http://bmjopen.bmj.com/site/about/guidelines.xhtml

For peer review only

The HADS was employed as a self-report measure of parental mental health [41]. It has been validated for use with the parents of children with disabilities [42]. It includes a total of fourteen items, with seven depression items (e.g., 'I feel as if I am slowed down') and seven anxiety items (e.g.,' I get sudden feelings of panic'). For the anxiety and depression scales scores in the 0-7 range are considered "normal", 8-10 "borderline and scores of 11-21 "abnormal"

Interview: The semi-structured interview schedule was developed to examine the experiences of parenting a child with additional needs during the pandemic. It aimed to identify the unique challenges experienced by families as well as resilience factors (S1 Supplemental materials).

Analysis

Questionnaire data were summarised using descriptive statistics. Interviews were analysed using reflective thematic analysis as described by Braun and Clarke [43–45]. Analysis was led by JW and LH. Adopting a critical realist framework, analyses involved identifying both semantic and latent meanings in the dataset following an inductive approach, whereby themes were generated in response to interview data, rather than trying to accommodate data within predefined themes or research questions [44]. JW and LH proceeded through the stages of data familiarisation, coding, theme development and review. Themes were reviewed following several detailed discussions with the other members of the research team DS and WM. Following these discussions, the final themes and sub-themes were confirmed. In a final stage, the full transcripts were coded by independent reviewers LW and TA following a codebook to ensure consistency.

Results

Situating the sample

23 parents took part. All the respondents were the mothers of IMAGINE-ID children; five were single parents. They reported on 14 boys and 9 girls aged 5 to 15 years old (M=9, SD=2.9). 78% of children received extra help at school or attended a special educational needs school. Most of the children were reported to be of white British ethnicity (n=19), other ethnicities included one mixed white and black child, one Irish child and one Asian child. The genetic make-up of the children was varied and included three children with sex chromosome aneuploidies, 16 with a CNV and four with an SNV (Table S1). All but three of the families included siblings. The Wessex scales indicated that the group included one non-verbal child. Two children were non-ambulant, 15 were partly mobile and the remainder were fully mobile. Seven children were not fully continent and only three children were reported to be literate. None had severe hearing or sight impairments.

On the SDQ 39% of children were rated to have behavioural difficulties in the "high -very high" severity bands. In 91% of children the behavioural difficulties were rated to have a "very high" impact on the family's day to day life. 35% percent of children had been diagnosed with an ASD and five had a diagnosis of Attention Deficit Disorder or Attention Deficit/Hyperactivity Disorder.

When rating themselves on the HADS for anxiety symptoms 17% of parents scored in the borderline range and 26% scored in the abnormal range. For depressive symptoms 22% scored in the borderline range and 4% in the abnormal range for anxiety symptoms.

Coronavirus impact: Two families suspected that their household had been exposed to someone likely to have Covid-19, but only one household reported having a member of the household test positive for Covid-19. Two families reported non-household family member deaths due to Covid-19. At the time of interviews none had a confirmed case in their immediate family.

Adapting to the restrictions: A quarter of families were shielding due to concerns about vulnerability to the virus. 83% reported that their child had had some difficulty in following the recommendations

For peer review only - http://bmjopen.bmj.com/site/about/guidelines.xhtml

for keeping away from close contact with people and 87% reported that the restrictions on leaving home had been stressful for their child. 61% of children's schools had been closed.

Financial stability: The pandemic had a financial impact on families, with 39% of respondents reporting that the pandemic had reduced their ability to earn money.

Emotional impact on child: 65% of children were reported to be worried about becoming infected and 35% were concerned about their physical health. Overall, the children were reported to be more worried about friends and family becoming infected by Covid-19 than themselves.

The pandemic led to a few unexpected positives, with 74% of families reporting that the pandemic had led to some positive changes in their child's life. These will be described in more detail in the qualitative results.

Thematic Analysis

Three main themes were identified, representing a range of experiences across different families. Overall, parents described experiencing similar difficulties to those they observed in other families during the lockdown, but with the additional challenge of their child's specific needs ("Managing pre-existing challenges"); they described unexpected benefits and unanticipated challenges ("Mixed emotions"), and they emphasised that personalised support was essential for their families to get through the lockdown ("Support matters"; Figure 1).

[Figure 1]

Managing pre-existing challenges, in a time of increased strain for everyone "you just feel like our life was so different to other people's"

Parents described the unique daily challenges they faced before the pandemic, emphasizing how different their lives were to those of families without children with special needs. Some families were surprised to find that some of the challenges brought on by the lockdown, were familiar.

"We've been in lockdown since he was born" Social distancing is the norm

Many families described that social distancing had been part of their daily life before the pandemic. Some aspects of social distancing, such as going to public places outside of peak hours, avoiding busy social settings and monitoring crowds, felt normal:

"We've been in lockdown since he was born. When we go on holiday we have to think about how busy it's going to be. We have to think about how many people are going to be there, if we're going to a restaurant; is it too noisy? Are there too many people? Are people too close to us? So we've always got social distancing in our heads anyway and we always have done. I wish other people could see (..) So that people with normal families could read it and think we're all going back to our normal life but these families aren't. It's definitely a different world that we live in."

For some, the social distancing measures did not have an impact on their social life, as they already had limited opportunities to socialise:

"To be honest for me it's not all that different than usual because I don't really see a lot of people anyway"

But for many it increased their sense of isolation:

"I think you just feel more isolated than you would normally, and it was always a bit like that on summer holiday. So if I go out and see all of the kids off and they're doing things that we could never really ever do, but it was just kind of that times 100, that feeling."

For peer review only - http://bmjopen.bmj.com/site/about/guidelines.xhtml

Planning for complex needs "there's a lot more things that I needed to do than the average sort of family"

Despite some aspects of lockdown being familiar, many felt the lockdown restrictions had created a new set of challenges to be navigated. Parents described having to manage an increase in behavioural problems, uncertainties in co-ordinating medical care, and new worries about explaining covid.

Fewer resources, but behavioural issues are the same

Managing behavioural difficulties and challenging behaviour was not uncommon for families prior to the pandemic. However, parents described the strain of managing these existing behavioural concerns with fewer resources, such as respite care, school, social care and their support networks. Some highlighted an increase in intensity and frequency of behavioural outbursts:

"Meltdowns, swearing, shouting, taking his frustrations out on us. On the furniture, chairs being knocked over, doors being slammed. So rather than being able to talk to us and say 'I don't like what's happening,' it came out in meltdowns and fairly intense."

In some cases, this contributed to the escalation of pre-existing behavioural outbursts and mental health problems, to the point of serious concern.

"I think the main thing that got me, and affected me the most was [child 1] saying, when he got so frustrated that he just wanted to kill himself."

Some parents noted the re-appearance of challenging behaviours that had previously been well managed:

"Yeah he gets very hyperactive, very loud we get a lot throwing. We were working with a child psychologist at school for about 5 years on a lot of his behaviours and a lot that we nipped in the bud and improved have come back definitely in the initial stage of the lockdown."

Changes in routine were the source of anxiety and behavioural outbursts for many children:

"His normal life completely changed. So the anxiety was more about 'I'm being told I can't do the things I would normally do.' And that then causes anxiety because he still hasn't got control over what he would normally do. (…) He needed to have some routine or some structure around his day."

***Medical* "All the appointments for the children have been cancelled"**

Most children had complex behavioural and medical needs, which require regular clinical monitoring. 91% of families described the cancellation or postponement of routine medical and social care appointments.

"It's been really bad. All the appointments for the children have been cancelled. I've literally got a list on my fridge of about 15 appointments that I have to chase up, and get re-booked in once they can do so"

Some parents expressed concerns about the impact of further delays to services for their children and were anxious about the impact of the disruptions to their child's care.

"From even like a medical point of view it's been really hard because you have had the meetings and like there's been delays and there's going to be delays like for my youngest daughter as well in terms of the under 5 system assessment waiting list and things like that"

Transition to telehealth "I don't think you can replace face to face with a telephone"

For peer review only - http://bmjopen.bmj.com/site/about/guidelines.xhtml

Most healthcare services started using tele-medicine during the pandemic. Parents described varied experiences of tele-health. Some enjoyed the experience and were grateful that their child's appointments could still go ahead:

"They were quite good, they said they're not seeing anybody but they did again do it virtually. So they did a phone call and then I emailed pictures and stuff, which is quite funny trying to do that! But at least kind of that was, they're still trying to do it."

Many parents described needing specialist face to face care due to the complex nature of their child's needs and abilities. In some cases it was challenging to communicate the complexity of their child's needs remotely.

"How can you explain to the physiotherapist how she walks over the phone? It's no good doing Zoom because obviously you still can't show, it's difficult so basically she's had to go off what I say and I've had to explain to her you know into detail and she done a report from there so that's difficult."

For others, the child would not have been able to communicate over the phone with professionals.

"Yeah well we had a CAMHS [Child and Adolescent Mental Health Service] meeting but it was over Zoom and I just phoned them and I cancelled it, I said there's not a hope in hell you're going to get him to talk to you over a phone, he won't speak to anybody on the phone he doesn't, he can't get that there's somebody on the other side of something"

Explaining Covid, changing rules and following rules: "How do you explain a pandemic?"

The level of understanding of the pandemic in the children varied substantially. Most had some awareness of the virus and the restriction measures, but some were unaware of the pandemic completely.

"I don't think he really understood at all, other than that he wasn't going to school and he wasn't doing what he normally does, but he really didn't know why. Even though on some levels his understanding is quite good and we can explain things to him. I mean how do you explain a pandemic?"

"I think he understands. I asked him yesterday how far two metres is: 'am I two metres away from you?' and he said 'no'. So he's got that sort of understanding."

Some families found using visual aids and social stories helpful to explain the effects of the pandemic to their child, but there were limits to how much could be conveyed in a simplified way:

"We asked for social stories, so he had a social story about washing his hands, about distancing, the fact that there was going to be a one-way system around the school. So by having that, we kind of prepared him for those things."

"Even if I would sign or show pictures, she wouldn't really understand it and I don't really know how she's going to understand like when the mask thing comes in, we've got to wear masks."

Many children had some difficulty applying the social distancing measures to friends and family. This meant that some parents modified their behaviour and avoided certain situations to keep their children safe.

"we've been doing shopping trips without him, but when we have sort of bumped into friends when we've been out and about he doesn't quite understand. We have to make sure to keep hold of him

For peer review only - http://bmjopen.bmj.com/site/about/guidelines.xhtml

because he wants to go and give auntie a cuddle and he doesn't quite understand that he can't do that"

Some children were reported to understand the rules and were keen to follow them. Some of these children found it difficult to understand why other people were not following the rules in public places, and at times attempted to police strangers about their rule-breaking behaviour:

"She's very good at rules. She's very interested in the Boris Johnson announcements and she takes them very literally. So if she sees somebody in the street not obeying social distancing law she won't think twice about telling them that: 'you're not social distancing and you need to be this far away'."

"Nice but hard": Mixed emotions around the challenges and unexpected benefits of lockdown

When reflecting on the overall experience of the pandemic, and particularly the lockdown, most parents had mixed emotions. Although there were many challenging moments, for many families there were also unexpected benefits which parents wanted to extend beyond the pandemic.

Desperate for hugs: "I don't like lockdown because I want snuggles with my nanny"

One common report from parents was that children were strongly impacted by not being able to see friends or extended family. Children missed both physical and emotional contact with others, and communicated that to their parents.

"He talks about the school and people he misses, so I know he does that when he misses friends or family – he mentions their name or with a question mark at the end quite clearly in his voice, that he's missing them and wants to see them."

Some children were also frustrated by not being able to take part in activities they normally enjoyed, whether because the activities were cancelled, or because the family was shielding.

"He's a nature boy he likes to go outside of the house, he loves going to places where there are animals or somewhere with a stream you know or that sort of thing, so he's sort of in his element there, so I think he's definitely missing out"

Happy at home: "Everyone's keeping distance from me and that's how I like it"

However, quite a few parents reported that their child with IDD was happy being at home during the lockdown. Many children felt that home was a safe and comforting place, where they could follow their own preferred routines and didn't have to worry about the anxieties of school or the outside world.

"She loved being in, she'd rather be at home any day. She'd shut herself away and she plays on her own a lot, so yeah it really was good for her. She loved it, she was well happy with it."

"It's been great, [child] doesn't like other people, he doesn't like crowds and he doesn't want to be interacting with other people so the government saying to other people you can't interact with people I think was great, we enjoyed it."

A few parents suggested that the lockdown had actually improved their child's mental health:

"He is much happier, much much happier during the pandemic. I wasn't worried about that side of things, he's happier in himself, he's more content, more confident, more self-belief in who he is as a person. He's got more resilient coming out of it."

Strained relationships: "Being constantly 24/7 together definitely did build up pressure"

For some families, staying physically safe had meant staying at home. Spending all their time together had put more pressure on relationships within and between parents and children:

For peer review only - http://bmjopen.bmj.com/site/about/guidelines.xhtml

"A lot of shouting, particularly with a hormonal 15-year-old and a dad who's quite stressed, even though he's working from home he's probably doing longer hours than he was in the office, so it's been sort of little things that've built up and then there's been explosions"

Strategies that parents would normally use to de-escalate conflict, such as giving children time by themselves, were no longer possible when families couldn't leave the house for weeks at a time.

"the relationship between him and his brother has been very difficult during lockdown because we've not been able to give them their space from each other, so it's not been easy"

As the lockdown continued for several months (and for longer than many initially expected), many parents felt like they were unable to have a break and it took its toll on the parents' wellbeing:

"Now we're just on survival"

Spending time together and slowing down: "It brought us a lot closer together"

The main positive experience of lockdown, which was reported by the majority of parents, was the opportunity to spend more time together as a family as everyday life became less busy. Parents described having more time to be able to cook, eat, and play together. Families felt closer, and enjoyed their stronger relationships – something they wanted to continue after the lockdown finished.

"in terms of our relationship, it's had a positive effect because we're eating more family meals together and we're just generally spending more time together"

"The boys have certainly enjoyed us all being together, we sit down every single evening together which we wouldn't normally and it would be nice if that continues"

Being able to take things more slowly had a positive impact on wellbeing for both parents and children, as they felt freed from the daily rush.

"Once we got in the swing of that, it was actually miles better because you didn't have to rush out for the bus, you know you're not working, you're not racing around trying to sort everything out. You can do everything at your own pace."

Some families said that because they were no longer commuting to and from school or work, they were able to get outside more, exercise, and take advantage of outdoor spaces away from other people.

"And then in the afternoon, I'm taking them out for a walk or a bike ride. So it's probably been more healthy. I'm fitter than I was. We've both got a better suntan that we didn't have. It's just slowed the pace of life down really. It's just been really nice"

A break from rushing between different medical or school appointments was appreciated, with some parents noting that it was worth the switch to online services as they no longer had to take the time and energy to travel often significant distances for their child's appointments.

"I think there's been less time pressure because you're not having to factor in travel, which may not seem like that much but when you add it up it ends up being quite a big chunk of time. So the fact that [child 1] has been doing her online English lessons, so previously I would have had to take her to a dyslexic centre which is 20 minutes there, 20 minutes back. So what is an hour's lesson turns into two hours, whereas now we just log on a minute before it starts and that's fine. And I think some of those things will carry on."

For peer review only - http://bmjopen.bmj.com/site/about/guidelines.xhtml

Support matters: Support tailored to the family's needs is helpful, but the format can make this feel less effective

One factor which strongly contributed to the family's overall experience during the lockdown was the support provided by external sources. This includes educational support from schools, either for home-schooling or providing in-person schooling where this took place, as well as support for their child's medical and psychological needs. The amount and type of support provided by these services varied significantly, with some parents reporting regular contact and others no contact at all.

Left behind: "I feel like they just left people who are vulnerable behind for two months."

Many parents described feeling left behind by a range of services and felt that their child's wellbeing was considered less important because of their additional needs:

"I just felt like he was forgotten and left to it, like his education doesn't matter because he has special needs".

"Like just the support and stuff, obviously for children with special needs hasn't been there."

Furthermore, many parents believed their needs had not been prioritised. They felt let down:

"I think there is definitely something to say for the fact that our community hasn't been mentioned and how have they been supported. Because I certainly haven't had much. We've had two emails from the local authority and the last email which I received a few weeks ago said we could use direct payments to buy equipment for our children or young people, should they need it. And I think that should've happened from day one, when there was a lockdown because lots of family can't just buy an iPad for their families. And I think if they told straight away, that would've helped a lot of families."

Checking in: "just being able to have that support bubble, rather than being locked in your own four walls"

In general, parents felt the most supported when any support was targeted to the child's individual needs, and when services were 'checking in' with parents, rather than waiting for problems to arise. Parents appreciated social and emotional support as much as, if not more than, practical support. This could come from individuals within services, or from friends and family, and was a way to let off steam when family life was difficult.

"I mean the other main thing to cope is – regularly calling up family or friends and having that contact. Because I'm with all the children I needed a break. So having that time to have a ten minute chat, to call, catch up. Tell someone how you're doing:' I'm having a stressful day mum, how are you?'and those kind of things. This just made things easier. I think just talking to someone on the phone, an adult, as opposed to children"

Parents appreciated getting support from SEN [Special Educational Needs] services in school, and felt better able to continue their child's education at home because they were able to ask questions and update the child's teachers on the amount of progress being made.

"We've had the SENCO [Special Educational Needs Co-ordinator] ringing us every three days checking in. She's also under the learning support service, that the school puts by for them and they support [child 1] for maths, but they've been ringing me every week. The head teacher has even phoned a couple of times. I know from speaking to people that that's not the norm, but we've felt very very much supported and I've found that I've been quite honest with them as well to be honest."

When parents did feel supported by medical and social services, this was usually the result of individuals reaching out, rather than a system-wide approach to support.

For peer review only - http://bmjopen.bmj.com/site/about/guidelines.xhtml

"'I've also got a caseworker. And she's been fantastic. She's been there if you needed her, she's dropped masks off to me. We've had hand sanitisers dropped off. So she's been an absolute gem. If I ever needed anything, again shopping, she would be more than happy to have done it. So I've had her support well so I've been quite lucky on that"

School is respite for everyone

Many parents struggled to support their child's home learning, despite their best efforts. Most felt that their child's SEN teachers had a level of expertise that couldn't be replaced:

"Home schooling has been really really [hard], I think that was the hardest bit for me definitely."

"The teacher dropped in some school work and he put it in the bin, I don't know how to teach him, he's severely learning disabled. (…) I mean he can just write his name he's nearly 10."

Approximately half of children's schools remained open throughout the lockdown. Most children had at least a few weeks of home-schooling, and while some returned to in-person schooling partially or fully, other children remained at home until the lockdown ended in July. Some parents explained that they sent their children back to school to diffuse tension in the home, or because they felt they were unable to provide a well-rounded education themselves.

"She had the opportunity to go back part time. Which we took up on because home schooling was just nearly impossible because of her needs."

"the school put out this maths curriculum and geography, history, lots of music, lots of art stuff we didn't touch any of that. [child 1] did a bit of music independently but we didn't do any, because I just didn't have the capacity to get through, we worked through the morning did English and maths and that was what we did"

In addition to the impact on academic subjects, some parents felt that their children's life skills and social skills had regressed due to the lack of contact with other children, and were concerned about the impact this was having.

"He's become a lot more withdrawn, I've seen quite a lot of regression in himself, his social skills. (…) We were quite worried about that because he was starting to make progress with it, he wouldn't start a conversation with a child, but if the child spoke to him he would interact sometimes, whereas before he was on his own, other kids didn't really exist."

1:1 Schooling is best: "I can't get [child] to pick up a pencil without some encouragement"

The lockdown offered parents a chance to reflect on their child's education provision. Most parents recognised that their child needed 1:1 support in order to engage with their education, whether at home or school. Sometimes this was already clear, and the child was already receiving support while at school; in other cases, it became apparent to the parent when they were trying to home-school the child themselves.

"Academically the lockdown helped him really if I'm honest just because you know he had that one-to-one and he's been given things a little bit more challenging you know, he's coped with it quite well"

"I think we give her more support with her learning when she's at home because everything is one to one. Whereas in school she's in a class, and I think she won't necessarily say she doesn't understand so she sometimes would just be there, and the whole lesson would go by and she won't actually have any idea what they're talking about but she won't have articulated that."

For peer review only - http://bmjopen.bmj.com/site/about/guidelines.xhtml

However, this level of support was not always possible during home education. Parents were often trying to juggle home-schooling for multiple children of various ages and abilities, sometimes while working from home themselves.

"They sent home some work, but none of it is work [Child] can do independently and they said they sent suggestions about activities, but we can't do the activities with [Child] and with [Child 2], cause we both have to work full-time from home"

Adaptations aren't always appropriate: "That is very difficult to do when you have special needs"

During the home-schooling period, schools made various attempts to adapt the educational experience to support parents and children, particularly supporting learning at home through online educational resources or teaching. However, many parents said that their child's level of intellectual disability, or other needs, meant that these resources were simply not accessible, or required constant supervision from a parent.

"They developed a really fantastic online programme, but for the kids who have special needs that's very difficult to access."

"He does virtually require one-to-one, he has that when he's at school so you can't really get much else done at home if you got to be keeping an eye on him"

Many parents felt that the level of work sent home was either too demanding, or not challenging enough for their child. Children with IDD who were in mainstream education were often sent the same work as their typically developing peers, and parents were offered little help in adapting this for their child's abilities, often having to rely on material that was inappropriate for their child's age.

"I think the biggest thing it would help it's special needs resources that aren't too babyish or patronising you know I'm getting work for him that meets his ability level academically but it's boring you know it's duckies on a pond and things like that"

"She's in year 7, and like for instance, her maths level is year 4, so the work that was sent to the year group that they were sending to everybody was like way beyond her level."

Parents reported feeling grateful when schools recognised that the targets or expectations for most children might not be achievable when home-schooling children with IDD.

"When I spoke to them they were okay, they said that 'it's okay, you don't have to complete it all, you do what you can do, we know your situation is different from other parents'. So, very understanding"

Access to technology: "I think the first thing to do is make sure everyone is able to access what they're providing"

A final factor contributing significantly to the success of home education and home care was families' access to resources and technology. Some parents reported that their school system provided them with resources to engage with online learning, which were appreciated.

"Anybody who was vulnerable or SEN got offered a tablet or a laptop. And most people took up on that. [Child] got his own laptop from school and that made a world of difference"

Parents appreciated being given the resources that they needed, or even better, given the financial support to get those resources themselves. This meant parents could identify what was needed and removed any redundancy in the support that was provided.

"We got a grant off the local carers centre, they phoned us and said [we] are giving out a grant to help you to stay at home if it's like for swings or patios or something like that"

For peer review only - http://bmjopen.bmj.com/site/about/guidelines.xhtml

Some families also received financial support and resources from local authorities or charities, who recognised that they had additional requirements compared to other families during the lockdown.

“Last week they sent an email saying that we’ve got a lockdown fund, it’s £100 per child and they get to choose stuff from Amazon that would directly benefit the child. Well they don’t do that funding when lockdown is off. So we’ve been really, I feel so I suppose really lucky with this stuff”

However, other families were not offered these same resources, or the resources they were given were not appropriate or accessible. This often made it harder to engage with online learning.

“But everyone is in the same boat, and well some children have to work on their phones because they didn’t have – because not everybody’s got laptops, have they? And even doing on a tablet is not easy. So doing like math is okay on a tablet because you’re just typing numbers but if you’re like writing a longer piece of writing or a history essay on a phone or a tablet, that’s a nightmare.”

For peer review only

Discussion

In addition to the challenges experienced by neurotypical families during the pandemic, parents of children with IDD have also had to manage the distinct challenges related to their children’s complex needs. Overall there was extensive variation in parents’ reported experiences; some of this variation is likely to reflect the wide range of needs of children in the IMAGINE-ID cohort. Although all children in the cohort have IDD, some children attended mainstream schools and had relatively low levels of support needs, whereas others had multiple physical and behavioural needs.

In line with previous research, we found that caregivers had experienced increased levels of distress [23,31–33] and the quantitative data supported an increase in anxiety (although not depression). However, most families were able to draw some positives from the pandemic, as they were able to enjoy spending more time together, as has been previously reported [33,24].

The findings also confirm reports that service provision for children with IDD had reduced or stopped during the pandemic [24,25]. The prevision of tailored support, specifically in regards to educational needs, varied substantially. Our findings provide more evidence to support the recommendations to keep schools and medical services open for children with special needs and highlight meaningful changes that can be implemented with few resources and at little financial cost. Our recommendation for future periods of confinement include:

1. ***Empower parents as experts***

Adapting existing coping strategies Parents were able to draw on their previous coping strategies, and their expertise on their own children, to meet their children’s needs as much as possible. They had a good sense of what helped their child to function in everyday life, and were able to adapt this to support them during the pandemic. For instance, many parents already used social stories to explain new events or situations, and made use of newly developing social stories to describe the pandemic and teach children to wash their hands. In some cases where these were not available, parents used pictures or tried to simplify explanations. Covid-relevant social stories and other resources have been developed since the start of the pandemic; we suggest that these are distributed freely and quickly to families of children with IDD to support parents.

Many children were reported to benefit from routines and consistency in their daily lives; these were often disrupted at the start of the lockdown as schools and services closed down and many families started shielding. However, where parents were able to introduce new routines, this seemed to benefit children, particularly when these routines allowed children more time at home doing activities they enjoyed.

For peer review only - http://bmjopen.bmj.com/site/about/guidelines.xhtml

Parents also responded to the restrictions around social gatherings, which might previously have been part of children's regular routines, by trying to avoid places or situations in which children might be used to interacting with others. For examples, avoiding parks and playgrounds where there were other children, as the children in this cohort might not be able to follow social distancing rules. However, it is important to emphasise that parents of children with IDD should not be expected to shut themselves off from the rest of society in the event of a pandemic – explicit guidance about the allowances for children with special needs, and communication of those allowances to the public, might help parents in maintaining their children's routines.

Guidance, with autonomy When describing their child's needs and how they tried to support them, parents often emphasised being the expert on their child. Specialised services and education were needed and often appreciated, but these only ever addressed one aspect of the child's daily needs. Parents can integrate multiple sources of support and ensure that it is targeted and relevant to their child. Supporting parents, and giving them the autonomy to make decisions with their children, therefore offers an effective way to support children. For instance, some parents described how useful it was being given grants to buy relevant equipment for their children's education and leisure, with the discretion to choose what they wanted to spend it on.

2. Promote tailored digital intervention

Hardware is a starting point, not a solution Access to technology is a starting point, but these resources need to be adapted in order to be inclusive. Without adaptations, digital homeschooling has the potential to increase inequalities. For example, some parents were given technology to support online schooling, without consideration of whether their child could actually access or benefit from those resources (although in many cases they are an important first step). Services wishing to support families in these conditions should therefore approach parents directly to identify where and how that support would be most appreciated. Effective support for some families will not necessarily be relevant for others, and an individualised approach is essential to understand what is needed, and the best ways to provide support.

Tele-medicine as complementary care delivery Tele-medicine is unlikely to be suitable for all IDD families. At this early stage of the pandemic, many medical and social services closed completely. However, by the time the interviews took place, some were starting to introduce tele-health services to keep in contact with the families in this study. Families enjoyed the flexibility that tele-medicine gave them, reducing the need to travel to appointments with children. However, there was a real sense that tele-health was not an appropriate substitute for in-person assessments, particular for children with IDD. Parents felt that they were not able to communicate their children's often complex needs over the phone or email, and the children themselves often struggled to engage with medical professionals who they couldn't see in front of them. While tele-medicine offers many opportunities to expand services, it is important to emphasise that this new mode of healthcare provision will not suit everyone, and families of children with IDD should be offered a range of options to ensure their child receives appropriate support. Reflecting a broader shift to digital services across healthcare provision, blended care will likely be the most appropriate approach going forward.

3. Support parent mental health to support the child

Fostering a sense of community Overall, the parents displayed great resilience, but this often came at the detriment of their own mental health and wellbeing. Where children needed high levels of personal care, parents often felt overwhelmed and unsupported, unable to access the respite care or specialised support they normally received. Many described high levels of distress and being close to burn-out. The questionnaire measures confirmed increased levels of anxiety.

For peer review only - http://bmjopen.bmj.com/site/about/guidelines.xhtml

Parents who reported feeling supported appeared to experience less distress. Social support for the parents in our study was often limited, particularly for lone parents. Many parents felt that online and telephone communication with friends and family did not provide enough support, and several felt isolated as their experience of parents of children with IDD was very different to that of other parents. Parents might benefit from the development of support networks with other families of children with IDD, to share the coping strategies and challenges that have been raised in this study. In the event of future lockdowns, a relaxation of the rules regarding household mixing might benefit lone parents so that they can identify their own 'support bubbles'. Similarly, working parents should be supported with more flexible work times and reduced work as necessary if they are also supporting their child's home learning. If this involves reducing or pausing work temporarily, financial assistance should be in place to ensure the family will not be disadvantaged.

Short but regular check-ins Regular check-ins with services and schools were described as very helpful. Parents who reported more positive experiences with online schooling tended to report frequent, individualised contact such as phone calls and emails with their children's teachers and support staff, which helped the parent adapt schoolwork for their child. A five-minute telephone call with each family once a week may be more effective than two hours working with the entire class, for children with special educational needs. Similarly, parents reported fewer concerns about their child's health when they were able to stay in contact with healthcare professionals who already knew the child. Even when medical appointments were cancelled or moved online, frequent communication with services meant parents felt supported during this time of uncertainty. Frequent, brief check-ins are a cheap and easy way to maintain the support available to families during times of crisis, and may reduce the risk of ongoing concerns developing into significant problems.

School as respite Schools provide much needed respite and specialist education for IDD families. Facilitating the re-opening of special schools and SEN units within mainstream schools could help mitigate some of the negative mental health impacts of future confinements on parents and IDD children. This could be achieved by prioritising the vaccination of school staff, in order to reassure parents that it is safe to send their child back to school.

Strengths, limitations and future directions

The study reflected the experiences of the mothers that were interviewed. Clearly, this small study cannot represent the full extent of the impact of the first UK lockdown on the IDD community. Concurrent large-scale questionnaire-based studies are needed to address this.

The interviews contained a few notable absences. We had anticipated reports of excessive hand-washing and cleanliness, however this was rarely mentioned in our interviews. We had also expected some families to express concern about being judged for needing allowances/exemptions, such as their child not wearing a mask when out in public, but this was rarely discussed. No one reported breaking the rules. Although this could be attributed to expectancy biases, we interpret this to be representative of the high national compliance during the first UK lockdown, particularly in individuals with pre-existing medical conditions [46].

The views presented here reflect families' experiences in the first few months of the pandemic and the first UK lockdown, a time of particularly heightened anxieties and stringent restrictions. At the time of writing the manuscript the government's response to the pandemic continues to evolve monthly. It is likely that parents' experiences and attitudes have changed over time. Therefore, we plan to conduct follow-up interviews in the spring of 2021, which may offer different insights as families have had more time to adapt to the 'new normal'. This will also allow us to explore concerns about how children with IDD have navigated the changes in rules [35-25] and explore the impact of a prolonged national crisis on the carers of children with IDD.

For peer review only - http://bmjopen.bmj.com/site/about/guidelines.xhtml

Conclusion

Parents of children with IDD experienced many challenges during the UK Covid-19 outbreak during the spring/summer of 2020. In addition to the demands of home-schooling, concern over infection, and lack of social connection that families across the country dealt with, families in this study had to meet the additional and often complex educational and medical needs of their children. Many parents felt let down and forgotten by medical and school services, and felt isolated from the rest of the country. However, parents also used their experience to develop coping strategies and found some unexpected benefits for their children, such as spending more time together at home. We can learn from these parents' experiences to provide guidelines for the future, to ensure these families are not forgotten.

Recommendations Summary:

Empower parents as experts
- Adapt existing coping strategies
- Guidance, but autonomy

Promote tailored digital intervention
  Hardware is a starting point, not a solution
  Tele-medicine as complementary care delivery

Support parent mental health to support the child
- Fostering a sense of community
- Short but regular check-ins
- School as respite

Funding statement: The IMAGINE-ID study is funded was funded by the Medical Research Council and Medical Research Foundation (MR/L011166/1 and MR/N022572/1). Research conducted at the CRF UCL GOS ICH is supported by the NIHR. The funders played no role in the design of the study and collection, analysis, interpretation of data or writing of the manuscript.

Competing interests statement: The authors declare that they have no competing interests.

Data availability statement: The datasets generated and/or analysed during the current study are not publicly available in order to protect the anonymity of those taking part, but are available from the corresponding author on reasonable request.

Author statement: JW, LH and DS designed and directed the study. JW and LH conducted the interviews and analysis (theme development and review). Themes were reviewed following several detailed discussions with the other members of the research team DS and WM. Transcripts were coded by independent reviewers LW and TA. The IMAGINE-ID consortium contributed to recruitment and manuscript revisions.

Acknowledgements: We would like to thank all the families who took part in this study and the IMAGINE-ID consortium for making this research possible.

For peer review only - http://bmjopen.bmj.com/site/about/guidelines.xhtml

Word count 8,606 (excluding boxes, tables, figures and references)

References

1 Maulik PK, Mascarenhas MN, Mathers CD, *et al.* Prevalence of intellectual disability: A meta-analysis of population-based studies. Res. Dev. Disabil. 2011;**32**:419–36. doi:10.1016/j.ridd.2010.12.018

2 American Association on Intellectual and Developmental Disabilities. *Intellectual disability : definition, classification, and systems of supports*. American Association on Intellectual and Developmental Disabilities 2010.

3 Gilissen C, Hehir-Kwa JY, Thung DT, *et al.* Genome sequencing identifies major causes of severe intellectual disability. *Nature* 2014;**511**:344–7. doi:10.1038/nature13394

4 Dekker MC, Koot HM, Van Der Ende J, *et al.* Emotional and behavioral problems in children and adolescents with and without intellectual disability. *J Child Psychol Psychiatry Allied Discip* 2002;**43**:1087–98. doi:10.1111/1469-7610.00235

5 Dykens EM. Annotation: Psychopathology in Children with Intellectual Disability. *J Child Psychol Psychiatry* 2000;**41**:407–17. doi:10.1111/1469-7610.00626

6 Einfeld S, Tonge B, Turner G, *et al.* Longitudinal course of behavioural and emotional problems of young persons with Prader-Willi, Fragile X, Williams and Down syndromes. *J Intellect Dev Disabil* 1999;**24**:349–54. doi:10.1080/13668259900034111

7 Totsika V, Hastings RP. Persistent challenging behaviour in people with an intellectual disability. *Curr Opin Psychiatry* 2009;**22**:437–41. doi:10.1097/YCO.0b013e32832cd9b8

8 Tonge B, Einfeld S. The trajectory of psychiatric disorders in young people with intellectual disabilities. *Aust N Z J Psychiatry* 2000;**34**:80–4. doi:10.1046/j.1440-1614.2000.00695.x

9 Tonge BJ, Einfeld SL. Psychopathology and intellectual disability: The Australian Child to Adult Longitudinal Study. In: Glidden LM, ed. . London: : Academic Press 2003. 61–91.

10 Emerson E, Hatton C. Mental health of children and adolescents with intellectual disabilities in Britain. *Br J Psychiatry* 2007;**191**:493–9. doi:10.1192/bjp.bp.107.038729

11 Emerson E, Einfeld S. Emotional and behavioural difficulties in young children with and without developmental delay: a bi-national perspective. *J Child Psychol Psychiatry* 2010;**51**:583–93. doi:10.1111/j.1469-7610.2009.02179.x

12 Emerson E, McCulloch A, Graham H, *et al.* Socioeconomic circumstances and risk of psychiatric disorders among parents of children with early cognitive delay. *Am J Intellect Dev Disabil* 2010;**115**:30–42. doi:10.1352/1944-7558-115.1.30

13 Emerson E, Shahtahmasebi S, Lancaster G, *et al.* Poverty transitions among families supporting a child with intellectual disability. *J Intellect Dev Disabil* 2010;**35**:224–34. doi:10.3109/13668250.2010.518562

14 Shahtahmasebi S, Emerson E, Berridge D, *et al.* Child disability and the dynamics of family poverty, hardship and financial strain: Evidence from the UK. *J Soc Policy* 2011;**40**:653–73. doi:10.1017/S0047279410000905

15 Mugno D, Ruta L, D'Arrigo VG, *et al.* Impairment of quality of life in parents of children and

For peer review only - http://bmjopen.bmj.com/site/about/guidelines.xhtml

adolescents with pervasive developmental disorder. *Health Qual Life Outcomes* 2007;**5**:22. doi:10.1186/1477-7525-5-22

16 Baker BL, McIntyre LL, Blacher J, *et al.* Pre-school children with and without developmental delay: Behaviour problems and parenting stress over time. *J Intellect Disabil Res* 2003;**47**:217–30. doi:10.1046/j.1365-2788.2003.00484.x

17 Gray KM, Piccinin AM, Hofer SM, *et al.* The longitudinal relationship between behavior and emotional disturbance in young people with intellectual disability and maternal mental health. *Res Dev Disabil* 2011;**32**:1194–204. doi:10.1016/j.ridd.2010.12.044

18 Emerson E, Robertson J, Wood J. Levels of Psychological Distress Experienced by Family Carers of Children and Adolescents with Intellectual Disabilities in an Urban Conurbation. *J Appl Res Intellect Disabil* 2004;**17**:77–84. doi:10.1111/j.1360-2322.2004.00183.x

19 Olsson MB, Hwang CP. Depression in mothers and fathers of children with intellectual disability. *J Intellect Disabil Res* 2001;**45**:535–43. doi:10.1046/j.1365-2788.2001.00372.x

20 White N, Hastings RP. Social and professional support for parents of adolescents with severe intellectual disabilities. *J Appl Res Intellect Disabil* 2004;**17**:181–90. doi:10.1111/j.1468-3148.2004.00197.x

21 Marquis S, Hayes M V., McGrail K. Factors Affecting the Health of Caregivers of Children Who Have an Intellectual/Developmental Disability. *J Policy Pract Intellect Disabil* 2019;**16**:201–16. doi:10.1111/jppi.12283

22 Holmes EA, O'Connor RC, Perry VH, *et al.* Multidisciplinary research priorities for the COVID-19 pandemic: a call for action for mental health science. The Lancet Psychiatry. 2020;**7**:547–60. doi:10.1016/S2215-0366(20)30168-1

23 Rose J, Willner P, Cooper V, *et al.* The effect on and experience of families with a member who has Intellectual and Developmental Disabilities of the COVID-19 pandemic in the UK: developing an investigation. *Int J Dev Disabil* 2020;:1–3. doi:10.1080/20473869.2020.1764257

24 Neece C, McIntyre LL, Fenning R. Examining the impact of COVID-19 in ethnically diverse families with young children with intellectual and developmental disabilities. *J Intellect Disabil Res* 2020;**64**:739–49. doi:10.1111/jir.12769

25 Eshraghi AA, Li C, Alessandri M, *et al.* COVID-19: overcoming the challenges faced by individuals with autism and their families. *The Lancet Psychiatry* 2020;**7**:481–3. doi:https://doi.org/10.1016/S2215-0366(20)30197-8

26 National Autistic Society. Left Stranded: The impact of coronavirus on autistic people and their families in the UK. 2020. https://www.autism.org.uk/what-we-do/news/coronavirus-report

27 Ameis SH, Lai M-C, Mulsant BH, *et al.* Coping, fostering resilience, and driving care innovation for autistic people and their families during the COVID-19 pandemic and beyond. *Mol Autism* 2020;**11**:61. doi:10.1186/s13229-020-00365-y

28 Jeste S, Hyde C, Distefano C, *et al.* Changes in access to educational and healthcare services for individuals with intellectual and developmental disabilities during COVID-19 restrictions. *J Intellect Disabil Res* 2020;**64**:825–33. doi:10.1111/jir.12776

29 Embregts PJCM, van den Bogaard KJHM, Frielink N, *et al.* A thematic analysis into the experiences of people with a mild intellectual disability during the COVID-19 lockdown

For peer review only - http://bmjopen.bmj.com/site/about/guidelines.xhtml

period. *Int J Dev Disabil* 2020;:1–5. doi:10.1080/20473869.2020.1827214

30 Courtenay K, Perera B. COVID-19 and people with intellectual disability: impacts of a pandemic. *Ir J Psychol Med* 2020;**37**:231–6. doi:10.1017/ipm.2020.45

31 Bellomo TR, Prasad S, Munzer T, *et al.* The impact of the COVID-19 pandemic on children with autism spectrum disorders. *J Pediatr Rehabil Med* 2020;**Preprint**:1–6. doi:10.3233/PRM-200740

32 Colizzi M, Sironi E, Antonini F, *et al.* Psychosocial and behavioral impact of COVID-19 in autism spectrum disorder: An online parent survey. *Brain Sci* 2020;**10**. doi:10.3390/brainsci10060341

33 Willner P, Rose J, Stenfert Kroese B, *et al.* Effect of the COVID-19 pandemic on the mental health of carers of people with intellectual disabilities. *J Appl Res Intellect Disabil* 2020;**n/a**. doi:10.1111/jar.12811

34 Cassidy S, Nicolaidis C, Davies B, *et al.* An Expert Discussion on Autism in the COVID-19 Pandemic. *Autism in Adulthood* 2020;**2**:106–17.

35 Lee J. Mental health effects of school closures during COVID-19. *Lancet Child Adolesc Heal* 2020;**4**:421. doi:10.1016/S2352-4642(20)30109-7

36 Pellicano L, Brett S, Den Houting J, *et al.* 'I want to see my friends': the everyday experiences of autistic people and thier familes during COVID-19. 2020.

37 Harris PA, Taylor R, Thielke R, *et al.* Research electronic data capture (REDCap)-A metadata-driven methodology and workflow process for providing translational research informatics support. *J Biomed Inform* 2009;**42**:377–81. doi:10.1016/j.jbi.2008.08.010

38 Nikolaidis A, Paksarian D, Alexander L, *et al.* The Coronavirus Health and Impact Survey (CRISIS) reveals reproducible correlates of pandemic-related mood states across the Atlantic. *medRxiv Prepr Serv Heal Sci* Published Online First: 27 August 2020. doi:10.1101/2020.08.24.20181123

39 Kushllck A, Blunden R, Cox G. A method of rating behaviour characteristics for use in large scale surveys of mental handicap. *Psychol Med* 1973;**3**:466–78. doi:10.1017/S0033291700054271

40 Goodman A, Lamping DL, Ploubidis GB. When to Use Broader Internalising and Externalising Subscales Instead of the Hypothesised Five Subscales on the Strengths and Difficulties Questionnaire (SDQ): Data from British Parents, Teachers and Children. *J Abnorm Child Psychol* 2010;**38**:1179–91. doi:10.1007/s10802-010-9434-x

41 Zigmond AS, Snaith RP. The Hospital Anxiety and Depression Scale. *Acta Psychiatr Scand* 1983;**67**:361–70. doi:10.1111/j.1600-0447.1983.tb09716.x

42 Hastings RP. Parental stress and behaviour problems of children with developmental disability. J. Intellect. Dev. Disabil. 2002;**27**:149–60. doi:10.1080/1366825021000008657

43 Braun V, Clarke V. Using thematic analysis in psychology. *Qual Res Psychol* 2006;**3**:77–101. doi:10.1191/1478088706qp063oa

44 Braun V, Clarke V. *Successful Qualitative Research: A practical Guide for Beginners*. SAGE Publications 2013.

45 Braun V, Clarke V. Reflecting on reflexive thematic analysis. *Qual Res Sport Exerc Heal* 2019;**11**:589–97. doi:10.1080/2159676X.2019.1628806

For peer review only - http://bmjopen.bmj.com/site/about/guidelines.xhtml

46 Krekel C, Swanke S, De Neve J-E, *et al.* Are Happier People More Compliant? Global Evidence From Three Large-Scale Surveys During Covid-19 Lockdowns. 2020. www.iza.org (accessed 5 Jan 2021).

For peer review only

For peer review only - http://bmjopen.bmj.com/site/about/guidelines.xhtml

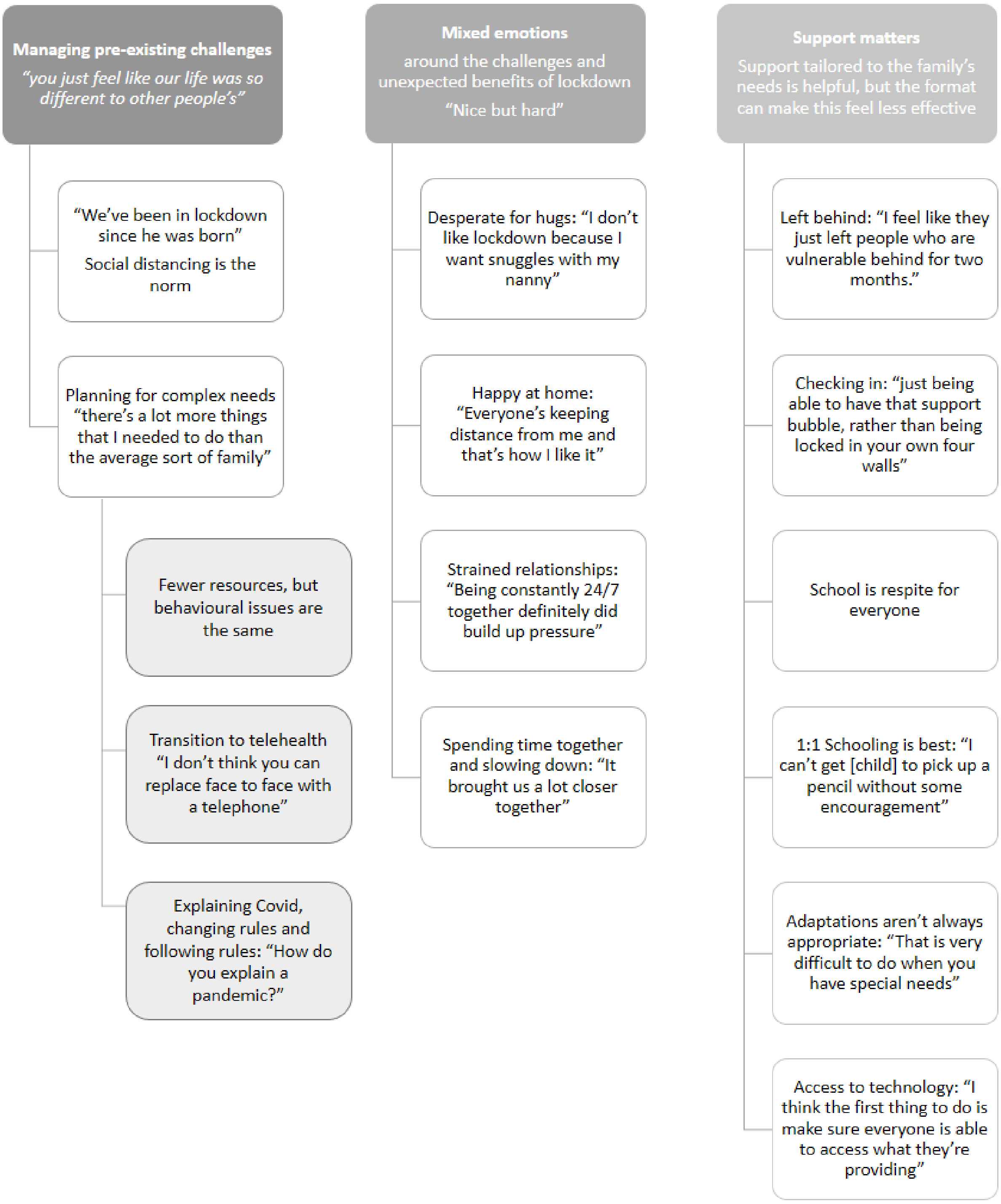

Figure 1: Thematic map of findings

Supplementary Materials

IMAGINE-ID Consortium Membership

[1] School of Clinical Medicine, University of Cambridge, Cambridge Biomedical Campus, Cambridge, UK

[2] Medical Research Council Centre for Neuropsychiatric Genetics and Genomics, Division of Psychological Medicine and Clinical Neurosciences, and Neuroscience and Mental Health Research Institute, Cardiff University, Cardiff, UK.

[3] NIHR BRC Great Ormond Street Institute of Child Health, University College London, London, UK

[4] Unique – The Rare Chromosome Disorder Support Group, London, UK.

Title (if applicable)	First Name	Surname	Institution
Dr	Kate	Baker	1
	Eleanor	Dewhurst	1
	Amy	Lafont	1
Professor	F Lucy	Raymond	1
	Terry	Shirley	1
	Hayley	Tilley	1
	Husne	Timur	1
	Catherine	Titterton	1
	Neil	Walker	1
	Sarah	Wallwork	1
	Francesca	Wicks	1
Dr	Zheng	Ye	1
	Marie	Erwood	1
	Sophie	Andrews	2
	Philippa	Birch	2
	Samantha	Bowen	2
	Karen	Bradley	2
	Aimee	Challenger	2
Dr	Samuel	Chawner	2
Dr	Andrew	Cuthbert	2
Professor	Jeremy	Hall	2
Professor	Peter	Holmans	2
	Sarah	Law	2
	Nicola	Lewis	2
	Sinead	Morrison	2
	Hayley	Moss	2
Professor Sir	Michael	Owen	2
	Sinead	Ray	2
	Matthew	Sopp	2
	Molly	Tong	2
Professor	Marianne	van den Bree	2
	Nadia	Coscini	3

For peer review only

	Sarah	Davies	3
	Spiros	Denaxas	3
	Hayley	Denyer	3
	Nasrtullah	Fatih	3
	Manoj	Juj	3
	Ellie	Kerry	3
	Anna	Lucock	3
Dr	William	Mandy	3
	Frida	Printzlau	3
Professor	David	Skuse	3
Dr	Ramya	Srinivasan	3
Dr	Susan	Walker	3
	Alice	Watkins	3
Dr	Jeanne	Wolstencroft	3
Dr	Beverly	Searle	4
Dr	Anna	Pelling	4

IMAGINE-ID Clinical Collaborators

**Title (if applicable)**	**First Name**	**Surname**	**Shorthand Institution**	**Genetics service**	**Hospital Trust**
Dr	John	Dean	Aberdeen	Aberdeen Royal Infirmary Genetics Service	NHS GRAMPIAN
Dr	Lisa	Robertson	Aberdeen	Aberdeen Royal Infirmary Genetics Service	NHS GRAMPIAN
Dr	Denise	Williams	Birmingham	West Midlands Regional Genetics Service	BIRMINGHAM WOMEN'S NHS FOUNDATION TRUST
Dr	Alan	Donaldson	Bristol	Bristol Clinical Genetics Service	UNIVERSITY HOSPITALS BRISTOL NHS FOUNDATION TRUST
Professor	Lucy	Raymond	Cambridge	East Anglian Medical Genetics Service	CAMBRIDGE UNIVERSITY HOSPITALS NHS FOUNDATION TRUST
Dr	Annie	Procter	Cardiff	All Wales Regional Genetics Service	CARDIFF AND VALE UNIVERSITY LHB
Dr	Jonathan	Berg	Dundee	Ninewells Hospital Dundee Genetics Service	NHS TAYSIDE

For peer review only

	Yanick	Crow	Edinburgh	Western General Hospital Edinburgh Genetics Service	NHS LOTHIAN
Professor	Anne	Lampe	Edinburgh	Western General Hospital Edinburgh Genetics Service	NHS LOTHIAN
Dr	Julia	Rankin	Exeter	Peninsula Genetics Service	ROYAL DEVON AND EXETER NHS FOUNDATION TRUST
Dr	Shelagh	Joss	Glasgow	Glasgow Genetics Centre	NHS GREATER GLASGOW & CLYDE
Professor	Lyn	Chitty	GOSH	London North East Thames Regional Genetics Service - Clinical Genetics	GREAT ORMOND STREET HOSPITAL FOR CHILDREN NHS FOUNDATION TRUST
Professor	Frances	Flinter	Guy's	London Guy's Hospital Genetic Centre	GUY'S AND ST THOMAS' NHS FOUNDATION TRUST
Dr	Muriel	Holder	Guy's	London Guy's Hospital Genetic Centre	GUY'S AND ST THOMAS' NHS FOUNDATION TRUST
Dr	Alison	Kraus	Leeds	Yorkshire Regional Genetics Service - Clinical Genetics	LEEDS TEACHING HOSPITALS NHS TRUST
Dr	Julian	Barwell	Leicester	Leicestershire Genetics Centre	UNIVERSITY HOSPITALS OF LEICESTER NHS TRUST
Dr	Pradeep	Vasudevan	Leicester	Leicestershire Genetics Centre	UNIVERSITY HOSPITALS OF LEICESTER NHS TRUST
Dr	Astrid	Weber	Liverpool	Cheshire & Merseyside Regional Genetic Service	LIVERPOOL WOMEN'S NHS FOUNDATION TRUST

For peer review only

Dr	William	Newman	Manchester	Manchester Centre for Genomic Medicine	CENTRAL MANCHESTER UNIVERSITY HOSPITALS NHS FOUNDATION TRUST
Dr	Miranda	Splitt	Newcastle	Northern Genetics Service	THE NEWCASTLE UPON TYNE HOSPITALS NHS FOUNDATION TRUST
Dr	Virginia	Clowes	North West Thames	London North West Thames Regional Genetics Service	NORTH WEST LONDON HOSPITALS NHS TRUST
Dr	Fleur	van Dijk	North West Thames	London North West Thames Regional Genetics Service	NORTH WEST LONDON HOSPITALS NHS TRUST
Dr	Rachel	Harrison	Nottingham	Nottingham Regional Genetics Service	NOTTINGHAM UNIVERSITY HOSPITALS NHS TRUST
Dr	Usha	Kini	Oxford	Oxford Genetics Service	OXFORD UNIVERSITY HOSPITALS NHS TRUST
Dr	Oliver	Quarrell	Sheffield	Sheffield Genetic Services	SHEFFIELD CHILDREN'S NHS FOUNDATION TRUST
Dr	Diana	Baralle	Southampton	Wessex Clinical Genetics Service	UNIVERSITY HOSPITAL SOUTHAMPTON NHS FOUNDATION TRUST
Dr	Sahar	Mansour	St George's	London South West Thames Regional Genetics Service	ST GEORGE'S HEALTHCARE NHS FOUNDATION TRUST

For peer review only

Supplemental materials

S1 Interview schedule

1. How do you feel the coronavirus pandemic is affecting you and your family, if at all? It may be helpful to consider your response in terms of routine, appointments, respite care, support networks, visiting family, childcare arrangements, work and related thoughts and feelings.

Prompts:

   a. Can you tell me a bit more about the coronavirus pandemic has changed your and your child's:
      i. Day to day life?
      ii. Childcare?
      iii. Access to services? Have any appointments been re-arranged, taken place online or been cancelled/postponed? How easy have you found accessing online/telephone appointments?
      iv. Seeing friends and family?
      v. Access to support networks?

2. How do you feel the coronavirus pandemic is affecting your ability to cope?

3. How do you feel the coronavirus pandemic is affecting your parenting skills?

4. How are you getting along as a family? / How is everyone getting along together?

5. How do you feel the physical (social) distancing and lockdown/isolation measures have affected you and your child/children during the coronavirus pandemic, if at all?
If this is affecting you, can you explain or list in what ways?
For example, consider the impact on your support network, social activities, social media and online socialising, and general contact with family, friends and services etc.

6. How do you feel that the easing of lockdown measures/restrictions are affecting you and your child/children, if at all? If this is affecting you, can you explain or list in what ways? For example, consider the impact on your support network, social activities, social media and online socialising, and general contact with family, friends and services etc.

7. In an ideal world, what resources or allowances would you need to manage lockdown/ to help your child?

8. Are there any coping strategies that you are using during this coronavirus pandemic? If so, what are these coping strategies and how have they helped you?

9. Have there been any unexpected positives during the pandemic? Is there anything from the lockdown that you would like to carry forward into everyday life for you/your child?

10. Is there anything else about your experiences during this pandemic that you would like to mention?

S2 Genetic variants of participants

For peer review only - http://bmjopen.bmj.com/site/about/guidelines.xhtml

Variant Type	Variants
Aneuploidy	48,XXYY
Aneuploidy	45,X
CNV	chr17p11.2 Duplication
CNV	chr18q12.3 Triplication
CNV	chr3q26.33-q27.1 Deletion
Aneuploidy	48,XXYY
CNV	chr7q11.23 Deletion
CNV	chr10q22.3-q23.2 Deletion
CNV	chr5q31.3 Duplication & chrXp22.1 Deletion
CNV	chr1q21.1-q21.2 Deletion
SNV	chr20p13 Missense & chrXq25 Missense
SNV	chrXp22.12 Splice site
CNV	chr16p13.11-p12.3 Duplication & chr7p21.1 Deletion
SNV	chr2q24.3 Missense
CNV	chr5q14.3 Deletion
SNV	chrXq26.1 Missense
CNV	chr16p13.11 Deletion
CNV	chr13q33.2-q33.3 Deletion
CNV	chr18p11.32-p11.21 & chrXp22.12 Duplication
CNV	chr7p21.1 Deletion
CNV	chr15q11.1-q13.1 Mosaic duplication
CNV	chr16p13.11-p12.3 Duplication
CNV	chr16p13.11 Deletion

S2 Table: Genetic variants of IMAGINE-ID children with IDD

For peer review only - http://bmjopen.bmj.com/site/about/guidelines.xhtml

BMJ Open

"We've been in lockdown since he was born": A mixed-methods exploration of the experiences of families caring for children with intellectual disability during the Covid-19 pandemic in the UK

Journal:	*BMJ Open*
Manuscript ID	bmjopen-2021-049386.R1
Article Type:	Original research
Date Submitted by the Author:	25-May-2021
Complete List of Authors:	Wolstencroft, Jeanne; University College London<br>Hull, Laura; University College London<br>Warner, Lauren; University College London<br>Akhtar, Tooba Nadeem; University College London<br>Mandy, W; University College London<br>IMAGINE, ID<br>Skuse, David ; University College London, Institute of Child Health
**Primary Subject Heading**:	Mental health
Secondary Subject Heading:	Paediatrics
Keywords:	COVID-19, MENTAL HEALTH, PAEDIATRICS, PSYCHIATRY

SCHOLARONE™
Manuscripts

I, the Submitting Author has the right to grant and does grant on behalf of all authors of the Work (as defined in the below author licence), an exclusive licence and/or a non-exclusive licence for contributions from authors who are: i) UK Crown employees; ii) where BMJ has agreed a CC-BY licence shall apply, and/or iii) in accordance with the terms applicable for US Federal Government officers or employees acting as part of their official duties; on a worldwide, perpetual, irrevocable, royalty-free basis to BMJ Publishing Group Ltd ("BMJ") its licensees and where the relevant Journal is co-owned by BMJ to the co-owners of the Journal, to publish the Work in this journal and any other BMJ products and to exploit all rights, as set out in our licence.

The Submitting Author accepts and understands that any supply made under these terms is made by BMJ to the Submitting Author unless you are acting as an employee on behalf of your employer or a postgraduate student of an affiliated institution which is paying any applicable article publishing charge ("APC") for Open Access articles. Where the Submitting Author wishes to make the Work available on an Open Access basis (and intends to pay the relevant APC), the terms of reuse of such Open Access shall be governed by a Creative Commons licence – details of these licences and which Creative Commons licence will apply to this Work are set out in our licence referred to above.

Other than as permitted in any relevant BMJ Author's Self Archiving Policies, I confirm this Work has not been accepted for publication elsewhere, is not being considered for publication elsewhere and does not duplicate material already published. I confirm all authors consent to publication of this Work and authorise the granting of this licence.

For peer review only

"We've been in lockdown since he was born": A mixed-methods exploration of the experiences of families caring for children with intellectual disability during the Covid-19 pandemic in the UK

Name of Authors
Jeanne Wolstencroft
Laura Hull
Lauren Warner
Tooba Akhtar
William Mandy
IMAGINE-ID consortium
David Skuse

Affiliations
Jeanne Wolstencroft (corresponding author), The Great Ormond Street Institute of Child Health, University College London, 30 Guilford Street, London, WC1N 1EH

Laura Hull, Division of Psychology and Language Sciences, Faculty of Brain Sciences, University College London, 26 Bedford Way, London, WC1H 0AP

Lauren Warner, The Great Ormond Street Institute of Child Health, University College London, 30 Guilford Street, London, WC1N 1EH

Tooba Akhtar, The Great Ormond Street Institute of Child Health, University College London, 30 Guilford Street, London, WC1N 1EH

William Mandy, Division of Psychology and Language Sciences, Faculty of Brain Sciences, University College London, 26 Bedford Way, London, WC1H 0AP

IMAGINE-ID consortium (see Imagine ID Consortium Membership list)

David Skuse, The Great Ormond Street Institute of Child Health, University College London, 30 Guilford Street, London, WC1N 1EH

Corresponding address and email
Jeanne Wolstencroft (corresponding author)
j.wolstencroft@ucl.ac.uk
The Great Ormond Street Institute of Child Health
University College London
30 Guilford Street
London, WC1N 1EH
0207 905 2168

Click here to enter text.

For peer review only

Abstract

Objectives: This study aimed to explore the experiences of parents caring for children with intellectual and developmental disability during the UK national lockdown in spring 2020, resulting from the Covid-19 pandemic. Design: Participants were identified using opportunity sampling from the IMAGINE-ID national (UK) cohort, and completed an online survey followed by a semi-structured interview. Interviews were analysed using thematic analysis. Setting: Interviews were conducted over the telephone in July 2020 as the first UK lockdown was ending. Participants: 23 mothers of children with intellectual and developmental disabilities aged 5 to 15 were recruited. Results: Themes reported by parents included: managing pre-existing challenges during a time of extreme change, having mixed emotions about the benefits and difficulties that arose during the lockdown, and the need for appropriate, individualised support. Conclusions: Our findings confirm observations previously found in UK parents of children with IDD and provide new insights on the use of technology during the pandemic for schooling and healthcare, as well as the need for regular check-ins.

Article Summary

Strengths and limitations of this study:

- This study recorded the experiences of parents who were caring for children with intellectual and developmental disabilities during the first UK national lockdown.
- Short questionnaires assessed the emotional wellbeing of parents and their children quantitatively.
- 23 caregivers were interviewed in July 2020 as the first UK lockdown was ending, and were asked to reflect on their experiences across the entire lockdown.
- Interviews were conducted over the telephone and transcripts were analysed using reflective thematic analysis.
- Only mothers were interviewed and so results may not reflect the experiences of other caregivers of children with intellectual and developmental disabilities.

Keywords

Intellectual disability, developmental disabilities, covid-19, pandemics, pandemics, mental health

For peer review only

Click here to enter text.

Introduction

Intellectual and developmental disabilities (IDD) affect approximately 1.8% of children worldwide [1].They are associated with significant limitations in cognitive and adaptive skills [2]. Genetic variation can be identified in up to 60% of IDD cases [3]. These changes can be large chromosomal abnormalities (such as aneuploidies and translocations), sub-microscopic deletions or duplications (such as copy number variants; CNV) or small changes in DNA sequence (such as single nucleotide variants; SNV).

The IMAGINE-ID consortium has recruited children with IDD of identified genetic aetiology from across the UK. The coronavirus (Covid-19) pandemic poses unique challenges to this cohort in two respects. Firstly, children with IDD have high levels of pre-existing health conditions and so may be considered especially vulnerable to the virus. As a result, families of these children are more likely than the general population to have been shielding, following UK government advice. Secondly, children with IDD are more likely to make use of services (such as specialist education, healthcare, and social care) which were suspended or diminished during the pandemic [4–8]. Calls have been made to prioritise the collection of high-quality data on the mental health effects of the pandemic, particularly within vulnerable groups [9,10].

In "normal times" children with IDD are at increased risk of behavioural difficulties compared to typically developing children [11–16]. Families of a child with IDD are more likely to live in deprived socio-economic circumstances [17–21], and experience increased parenting stress or parental psychological distress [22–28] than typically developing children. Early reports of the impact of the pandemic on children with IDD and their parents suggest that many were feeling overwhelmed, forgotten and had experienced high levels of anxiety and depression [9,29–35].

International surveys and interviews with families of children with IDD or physical disabilities suggest that Covid-19 national lockdowns have led to increased behavioural problems for children [36], as well as reduced opportunities to socialise [4]. Parents have also reported concerns about their child's safety, and that children may fall behind developmentally because of reduced access to services [37].

The nature and extent of Covid-19 restrictions has varied across different countries, with the UK being particularly severely impacted by infections. It is therefore important to assess the experiences of families in the UK in addition to the emerging international literature. One previous qualitative study has examined the experiences of 8 mothers of children with IDD in the UK [33]. These authors found that mothers felt under pressure to provide their child with educational and therapeutic provisions, which had been removed or reduced because of the pandemic. Mothers reported a lack of support from additional services, leading to increased stress and uncertainty, but also enjoyed the freedom from routine with several noting the positive impact it had for their child's wellbeing.

We interviewed the caregivers of children with IDD, who have been caring for their children at home during the pandemic. The present study was carried out concurrently with those described above, and we seek to extend their useful findings in the IMAGINE-ID cohort to identify consistencies across research as well as any additional insights from the parents in our cohort.

Aims and objectives

We sought to understand the experiences of families caring for a child with IDD during the Covid-19 pandemic in order to identify key targets for support. We interviewed 23 IMAGINE-ID families mid-July 2020. At this time, the lockdown restrictions were eased in parts of the UK, but the government advised vulnerable individuals to continue to shield until the 1st of August 2020. We asked families to reflect on their experiences across the entirety of the first national lockdown. Our objectives

Click here to enter text.

For peer review only - http://bmjopen.bmj.com/site/about/guidelines.xhtml

were: (1) to identify the areas of difficulty and resilience during the pandemic and de-confinement; (2) to identify key targets for support.

Methods

Study Design

The IMAGINE-ID programme of research, funded by the UK Medical Research Council (MRC) from 2014 to 2024, recruited a cohort of 3,402 UK families whose child had IDD due to an identified genetic anomaly (www.imagine-id.org). IMAGINE-ID caregivers with children under the age of 16 were invited to take part through the study newsletter. Parents were enrolled in the current study through consecutive sampling due to the time restrictions, so that all participants were interviewed prior to end of shielding in the first UK lockdown. A mixed-methods approach was used to capture the diversity of challenges encountered and resilience factors drawn upon by families. Data were collected by parent report using online questionnaires and a semi-structured telephone interview (n=23). Questionnaire data were collected and managed using REDCap electronic data capture tools hosted at University College London [38]. Telephone interviews were recorded and transcribed by the research team. Ethical approval for the study was obtained from UCL Research Ethics Committee (9553/003), and all participants consented to take part and for their interviews to be recorded.

Patient and Public Involvement

The research objectives were developed in response to conversations with collaborators at the UNIQUE rare chromosome disorder charity. A summary of the results will be disseminated to participants and the IMAGINE-ID cohort via the study newsletter, website and social media accounts.

Measures

Coronavirus Impact: An adapted version of the National Institute of Health CoRonavIruS Health Impact Survey (CRISIS) was used to assess the family's adaptations to the pandemic, including key domains relevant to mental distress and resilience [39].

Child Characteristics: Demographic details and genetic data about the child were extracted from the IMAGINE-ID database. The Wessex Scales were used to gain an updated measure of adaptive function and degree of IDD [40]. The measure assesses self-help skills, literacy, mobility and incontinence.

Wellbeing: The wellbeing of the child and the parent were assessed using the Strengths and Difficulties Questionnaire (SDQ) and the Hospital Anxiety and Depression scale (HADS) respectively The SDQ is a well-validated behavioural screening questionnaire for children and has been validated with the parents of children with disabilities [41]. The SDQ includes scales that measure emotional symptoms, conduct problems, hyperactivity/impulsivity and inattention difficulties, peer relationship problems and prosocial behaviour. The first four scales are combined to create a total difficulties score. On the total difficulties scale scores of 17-19 are indicative of "high" levels of behavioural difficulty and 20-40 are considered "very high" levels of behavioural difficulty compared to population norms. An additional impact scale measures the impact of this composite score on daily life. Scores of 3-10 on the impact scale are in the "very high" range compared to population samples. The HADS was employed as a self-report measure of parental mental health [42]. It has been validated for use with the parents of children with disabilities [43]. It includes a total of fourteen items, with seven depression items (e.g., 'I feel as if I am slowed down') and seven anxiety items (e.g.,' I get sudden feelings of panic'). For the anxiety and depression scales scores in the 0-7 range are considered "normal", 8-10 "borderline and scores of 11-21 "abnormal"

Interview: The semi-structured interview schedule was developed to examine the experiences of parenting a child with additional needs during the pandemic. It aimed to identify the unique

Click here to enter text.

For peer review only - http://bmjopen.bmj.com/site/about/guidelines.xhtml

challenges experienced by families as well as resilience factors. Questions invited caregivers to reflect on their experiences of the lockdown, including how their day to day life had changed, how they'd coped and identifying coping strategies they had implemented. Parents were asked about their experiences across the UK lockdown, including reflecting back on their experiences during the period of stricter restrictions, as well as the easing of restrictions taking place at the time of the interview (see Supplemental materials for the exact questions asked). Interviews ranged from 13 minutes to 1 hour and 25 minutes, with a mean length of 44 minutes, and all interviews were conducted by JW and LH.

Analysis

Questionnaire data were summarised using descriptive statistics. De-identified transcripts of interviews were analysed using reflective thematic analysis as described by Braun and Clarke [44–46]. Analysis was led by JW and LH. Adopting a critical realist framework, analyses involved identifying both semantic and latent meanings in the dataset following an inductive approach, whereby themes were generated in response to interview data, rather than trying to accommodate data within predefined themes or research questions [45]. The lead authors (JW and LH) are research psychologists, whose theoretical stance is grounded in a social model of disability. JW's research background is focused on understanding the impact of rare genetic disorders on the neurodevelopment of children with rare genetic disorders. LH's expertise is in camouflaging in autism, using qualitative and quantitative methods to explore characteristics related to identity, mental health, and support. None of the authors have IDD themselves, but there is personal and professional experience of IDD amongst authors.

JW and LH proceeded through the stages of data familiarisation, coding, theme development and review. Themes were reviewed following several detailed discussions with the other members of the research team DS and WM. Following these discussions, the final themes and sub-themes were confirmed. In a final stage, the full transcripts were coded by independent reviewers LW and TA following a codebook to ensure consistency.

Results

Situating the sample

23 parents took part in interviews. All the respondents were the mothers of IMAGINE-ID children; five were single parents. They reported on 14 boys and 9 girls aged 5 to 15 years old (M=9, SD=2.9). 78% of children received extra help at school or attended a special educational needs school. Most of the children were reported to be of white British ethnicity (n=19), other ethnicities included one mixed white and black child, one Irish child and one Asian child. The genetic make-up of the children was varied and included three children with sex chromosome aneuploidies, 16 with a CNV and four with an SNV (Table S1). All but three of the families included siblings. The Wessex scales indicated that the group included one non-verbal child. Two children were non-ambulant, 15 were partly mobile and the remainder were fully mobile. Seven children were not fully continent and only three children were reported to be literate. None had severe hearing or sight impairments (Table 1).

On the SDQ 39% of children were rated to have behavioural difficulties in the "high -very high" severity bands. In 91% of children the behavioural difficulties were rated to have a "very high" impact on the family's day to day life. 35% percent of children had been diagnosed with an ASD and five had a diagnosis of Attention Deficit Disorder or Attention Deficit/Hyperactivity Disorder (Table 1).

For peer review only - http://bmjopen.bmj.com/site/about/guidelines.xhtml

When rating themselves on the HADS for anxiety symptoms 17% of parents scored in the borderline range and 26% scored in the abnormal range. For depressive symptoms 22% scored in the borderline range and 4% in the abnormal range (Table 1).

Coronavirus impact: Two families suspected that their household had been exposed to someone likely to have Covid-19, but only one household reported having a member of the household test positive for Covid-19. Two families reported non-household family member deaths due to Covid-19. At the time of interviews none had a confirmed case in their immediate family.

Adapting to the restrictions: A quarter of families were shielding due to concerns about vulnerability to the virus. 83% reported that their child had had some difficulty in following the recommendations for keeping away from close contact with people and 87% reported that the restrictions on leaving home had been stressful for their child. 61% of children's schools had been closed.

Financial stability: The pandemic had a financial impact on families, with 39% of respondents reporting that the pandemic had reduced their ability to earn money.

Emotional impact on child: 65% of children were reported to be worried about becoming infected and 35% were concerned about their physical health. Overall, the children were reported to be more worried about friends and family becoming infected by Covid-19 than themselves.

The pandemic led to a few unexpected positives, with 74% of families reporting that the pandemic had led to some positive changes in their child's life. These will be described in more detail in the qualitative results.

[Table 1]

Thematic Analysis

Three main themes were identified, representing a range of experiences across different families. Overall, parents described the difficulty of adjusting to the pandemic with the additional challenge of their child's specific needs ("Managing pre-existing challenges"); they described unexpected benefits and unanticipated challenges ("Mixed emotions"), and they emphasised that personalised support was essential for their families to get through the lockdown ("Support matters"; Table 2).

[Table 2]

Theme 1. Managing pre-existing challenges, in a time of increased strain for everyone "you just feel like our life was so different to other people's"

Parents described the unique daily challenges they faced before the pandemic, emphasizing how different their lives were to those of families without children with special needs. Some families were surprised to find that some of the challenges brought on by the lockdown, were familiar.

Sub-theme 1.1 "We've been in lockdown since he was born" Social distancing is the norm

Many families described that social distancing had been part of their daily life before the pandemic. Some aspects of social distancing, such as going to public places outside of peak hours, avoiding busy social settings and monitoring crowds, felt normal:

"We've been in lockdown since he was born. When we go on holiday we have to think about how busy it's going to be. We have to think about how many people are going to be there, if we're going to a restaurant; is it too noisy? Are there too many people? Are people too close to us? So we've always got social distancing in our heads anyway and we always have done. I wish other people could see (..) So that people with normal families could read it and think we're all going back to our normal life but these families aren't. It's definitely a different world that we live in." (P23)

For peer review only - http://bmjopen.bmj.com/site/about/guidelines.xhtml

For some, the social distancing measures did not have an impact on their social life, as they already had limited opportunities to socialise:

"To be honest for me it's not all that different than usual because I don't really see a lot of people anyway" (P4)

But for many it increased their sense of isolation:

"I think you just feel more isolated than you would normally, and it was always a bit like that on summer holiday. So if I go out and see all of the kids off and they're doing things that we could never really ever do, but it was just kind of that times 100, that feeling." (P12)

Sub-theme 1.2 Left behind: "I feel like they just left people who are vulnerable behind for two months."

Many parents described feeling left behind by a range of services and felt that their child's wellbeing was considered less important because of their additional needs:

"I just felt like he was forgotten and left to it, like his education doesn't matter because he has special needs". (P12)

"Like just the support and stuff, obviously for children with special needs hasn't been there." (P14)

Furthermore, many parents believed their needs had not been prioritised. They felt let down in comparison to the support that had been offered to other families:

"I think there is definitely something to say for the fact that our community hasn't been mentioned and how have they been supported. Because I certainly haven't had much." (P20)

Sub-theme 1.3 Planning for complex needs "there's a lot more things that I needed to do than the average sort of family"

Despite some aspects of lockdown being familiar, many felt the lockdown restrictions had created a new set of challenges to be navigated. Parents described having to manage an increase in behavioural problems, uncertainties in co-ordinating medical care, and new worries about explaining covid.

Fewer resources, but behavioural issues are the same

Managing behavioural difficulties and challenging behaviour was not uncommon for families prior to the pandemic. However, parents described the strain of managing these existing behavioural concerns with fewer resources, such as respite care, school, social care and their support networks. Some highlighted an increase in intensity and frequency of behavioural outbursts:

"Meltdowns, swearing, shouting, taking his frustrations out on us. On the furniture, chairs being knocked over, doors being slammed. So rather than being able to talk to us and say 'I don't like what's happening,' it came out in meltdowns and fairly intense." (P18)

In some cases, this contributed to the escalation of pre-existing behavioural outbursts and mental health problems, to the point of serious concern.

"I think the main thing that got me, and affected me the most was [child 1] saying, when he got so frustrated that he just wanted to kill himself." (P14)

Some parents noted the re-appearance of challenging behaviours that had previously been well managed:

For peer review only - http://bmjopen.bmj.com/site/about/guidelines.xhtml

"Yeah he gets very hyperactive, very loud we get a lot throwing. We were working with a child psychologist at school for about 5 years on a lot of his behaviours and a lot that we nipped in the bud and improved have come back definitely in the initial stage of the lockdown." (P10)

Changes in routine were the source of anxiety and behavioural outbursts for many children:

"His normal life completely changed. So the anxiety was more about 'I'm being told I can't do the things I would normally do.' And that then causes anxiety because he still hasn't got control over what he would normally do. (…) He needed to have some routine or some structure around his day." (P18)

Explaining Covid, changing rules and following rules: "How do you explain a pandemic?"

The level of understanding of the pandemic in the children varied substantially. Most had some awareness of the virus and the restriction measures, but some were unaware of the pandemic completely.

"I don't think he really understood at all, other than that he wasn't going to school and he wasn't doing what he normally does, but he really didn't know why. Even though on some levels his understanding is quite good and we can explain things to him. I mean how do you explain a pandemic?" (P10)

"I think he understands. I asked him yesterday how far two metres is: 'am I two metres away from you?' and he said 'no'. So he's got that sort of understanding." (P17)

Some families found using visual aids and social stories helpful to explain the effects of the pandemic to their child, but there were limits to how much could be conveyed in a simplified way:

"We asked for social stories, so he had a social story about washing his hands, about distancing, the fact that there was going to be a one-way system around the school. So by having that, we kind of prepared him for those things." (P18)

Many children had some difficulty applying the social distancing measures to friends and family. This meant that some parents modified their behaviour and avoided certain situations to keep their children safe.

"We've been doing shopping trips without him, but when we have sort of bumped into friends when we've been out and about he doesn't quite understand. We have to make sure to keep hold of him because he wants to go and give auntie a cuddle and he doesn't quite understand that he can't do that" (P10)

Some children were reported to understand the rules and were keen to follow them. Some of these children found it difficult to understand why other people were not following the rules in public places, and at times attempted to police strangers about their rule-breaking behaviour:

"She's very good at rules. She's very interested in the Boris Johnson announcements and she takes them very literally. So if she sees somebody in the street not obeying social distancing law she won't think twice about telling them that: 'you're not social distancing and you need to be this far away'." (P22)

Theme 2. Mixed emotions around the challenges and unexpected benefits of lockdown: "The pandemic was nice but really hard"

When reflecting on the overall experience of the pandemic, and particularly the lockdown, most parents had mixed emotions. Although there were many challenging moments, for many families there were also unexpected benefits which parents wanted to extend beyond the pandemic.

For peer review only - http://bmjopen.bmj.com/site/about/guidelines.xhtml

Sub-theme 2.1 Desperate for hugs: "I don't like lockdown because I want snuggles with my nanny"

One common report from parents was that children were strongly impacted by not being able to see friends or extended family. Children missed both physical and emotional contact with others, and communicated that to their parents.

"He talks about the school and people he misses, so I know he does that when he misses friends or family – he mentions their name or with a question mark at the end quite clearly in his voice, that he's missing them and wants to see them." (P20)

Some children were also frustrated by not being able to take part in activities they normally enjoyed, whether because the activities were cancelled, or because the family was shielding.

"He's a nature boy he likes to go outside of the house, he loves going to places where there are animals or somewhere with a stream you know or that sort of thing, so he's sort of in his element there, so I think he's definitely missing out" (P2)

Sub-theme 2.2 Happy at home: "Everyone's keeping distance from me and that's how I like it"

However, quite a few parents reported that their child with IDD was happy being at home during the lockdown. Many children felt that home was a safe and comforting place, where they could follow their own preferred routines and didn't have to worry about the anxieties of school or the outside world.

"She loved being in, she'd rather be at home any day. She'd shut herself away and she plays on her own a lot, so yeah it really was good for her. She loved it, she was well happy with it." (P7)

"It's been great, [child] doesn't like other people, he doesn't like crowds and he doesn't want to be interacting with other people so the government saying to other people you can't interact with people I think was great, we enjoyed it." (P23)

A few parents suggested that the lockdown had actually improved their child's mental health:

"He is much happier, much much happier during the pandemic. I wasn't worried about that side of things, he's happier in himself, he's more content, more confident, more self-belief in who he is as a person. He's got more resilient coming out of it." (P1)

However, some parents felt that their children's life skills and social skills had regressed due to the lack of contact with other children, and were concerned about the impact this was having.

"He's become a lot more withdrawn, I've seen quite a lot of regression in himself, his social skills." (P2)

Sub-theme 2.3 Strained relationships: "Being constantly 24/7 together definitely did build up pressure"

For some families, staying physically safe had meant staying at home. Spending all their time together had put more pressure on relationships within and between parents and children:

"A lot of shouting, particularly with a hormonal 15-year-old and a dad who's quite stressed, even though he's working from home he's probably doing longer hours than he was in the office, so it's been sort of little things that've built up and then there's been explosions" (P10)

Strategies that parents would normally use to de-escalate conflict, such as giving children time by themselves, were no longer possible when families couldn't leave the house for weeks at a time.

Click here to enter text.

For peer review only - http://bmjopen.bmj.com/site/about/guidelines.xhtml

"The relationship between him and his brother has been very difficult during lockdown because we've not been able to give them their space from each other, so it's not been easy" (P19)

As the lockdown continued for several months (and for longer than many initially expected), many parents felt like they were unable to have a break and it took its toll on the parents' wellbeing:

"Now we're just on survival" (P19)

Sub-theme 2.4 Spending time together and slowing down: "It brought us a lot closer together"

The main positive experience of lockdown, which was reported by the majority of parents, was the opportunity to spend more time together as a family as everyday life became less busy. Parents described having more time to be able to cook, eat, and play together. Families felt closer, and enjoyed their stronger relationships – something they wanted to continue after the lockdown finished.

"In terms of our relationship, it's had a positive effect because we're eating more family meals together and we're just generally spending more time together" (P13)

Being able to take things more slowly had a positive impact on wellbeing for both parents and children, as they felt freed from the daily rush. A break from rushing between different medical or school appointments was appreciated, with some parents noting that it was worth the switch to online services as they no longer had to take the time and energy to travel often significant distances for their child's appointments.

"Once we got in the swing of that, it was actually miles better because you didn't have to rush out for the bus, you know you're not working, you're not racing around trying to sort everything out. You can do everything at your own pace." (P9)

Theme 3. Support matters

One factor which strongly contributed to the family's overall experience during the lockdown was the support provided by external sources. This includes educational support from schools, either for home-schooling or providing in-person schooling where this took place, as well as support for their child's medical and psychological needs. The amount and type of support provided by these services varied significantly, with some parents reporting regular contact and others no contact at all.

Sub-theme 3.1 Transition to telehealth "I don't think you can replace face to face with a telephone"

Most children had complex behavioural and medical needs, which require regular clinical monitoring. 91% of families described the cancellation or postponement of routine medical and social care appointments.

"It's been really bad. All the appointments for the children have been cancelled. I've literally got a list on my fridge of about 15 appointments that I have to chase up, and get re-booked in once they can do so" (P14)

Most healthcare services started using tele-medicine during the pandemic. Parents described varied experiences of tele-health. Some enjoyed the experience and were grateful that their child's appointments could still go ahead:

"They were quite good, they said they're not seeing anybody but they did again do it virtually. So they did a phone call and then I emailed pictures and stuff, which is quite funny trying to do that! But at least kind of that was, they're still trying to do it." (P12)

For peer review only - http://bmjopen.bmj.com/site/about/guidelines.xhtml

Many parents described needing specialist face to face care due to the complex nature of their child's needs and abilities. In some cases it was challenging to communicate the complexity of their child's needs remotely.

"How can you explain to the physiotherapist how she walks over the phone? It's no good doing Zoom because obviously you still can't show, it's difficult so basically she's had to go off what I say and I've had to explain to her you know into detail and she done a report from there so that's difficult." (P16)

For others, the child was not able to communicate over the phone with professionals, and so was not able to access the support that was offered.

"Yeah well we had a CAMHS [Child and Adolescent Mental Health Service] meeting but it was over Zoom and I just phoned them and I cancelled it, I said there's not a hope in hell you're going to get him to talk to you over a phone, he won't speak to anybody on the phone he doesn't, he can't get that there's somebody on the other side of something" (P23)

Sub-theme 3.2 Considering equality and equity in remote schooling

Equality through access to technology: "I think the first thing to do is make sure everyone is able to access what they're providing"

Approximately half of children's schools remained open throughout the lockdown. Most children had at least a few weeks of home-schooling, and while some returned to in-person schooling partially or fully, other children remained at home until the lockdown ended in July. During the home-schooling period, schools made various attempts to adapt the educational experience to support parents and children, particularly supporting learning at home through online educational resources or teaching. Some parents reported that their school system provided them with resources to engage with online learning, which were appreciated.

"Anybody who was vulnerable or SEN got offered a tablet or a laptop. And most people took up on that. [Child] got his own laptop from school and that made a world of difference" (P8)

However, other families were not offered these same resources, or the resources they were given were not appropriate or accessible. This often made it harder to engage with online learning.

"But everyone is in the same boat, and some children have to work on their phones because not everybody's got laptops, have they? And even doing on a tablet is not easy. So doing like math is okay on a tablet because you're just typing numbers but if you're like writing a longer piece of writing or a history essay on a phone or a tablet, that's a nightmare." (P9)

Equity of access lagging behind as adaptations aren't always appropriate

However, many parents said that their child's level of intellectual disability, or other needs, meant that these resources were simply not accessible, or required constant supervision from a parent.

"They developed a really fantastic online programme, but for the kids who have special needs that's very difficult to access." (P11)

Many parents felt that the level of work sent home was either too demanding, or not challenging enough for their child. Children with IDD who were in mainstream education were often sent the same work as their typically developing peers, and parents were offered little help in adapting this for their child's abilities, often having to rely on material that was inappropriate for their child's age.

For peer review only - http://bmjopen.bmj.com/site/about/guidelines.xhtml

"I think the biggest thing it would help it's special needs resources that aren't too babyish or patronising you know I'm getting work for him that meets his ability level academically but it's boring you know it's duckies on a pond and things like that" (P17)

Sub-theme 3 Checking in: "just being able to have that support bubble, rather than being locked in your own four walls"

In general, parents felt the most supported when any support was targeted to the child's individual needs, and when services were 'checking in' with parents, rather than waiting for problems to arise. Parents appreciated social and emotional support as much as, if not more than, practical support. This could come from individuals within services, or from friends and family, and was a way to let off steam when family life was difficult.

"I mean the other main thing to cope is – regularly calling up family or friends and having that contact. Because I'm with all the children I needed a break. So having that time to have a ten minute chat, to call, catch up. Tell someone how you're doing: 'I'm having a stressful day mum, how are you?' and those kind of things. This just made things easier. I think just talking to someone on the phone, an adult, as opposed to children" (P21)

Parents appreciated getting support from Special Educational Needs services in school, and felt better able to continue their child's education at home because they were able to ask questions and update the child's teachers on the amount of progress being made.

"We've had the SENCO [Special Educational Needs Co-ordinator] ringing us every three days checking in. She's also under the learning support service, that the school puts by for them and they support [child 1] for maths, but they've been ringing me every week. The head teacher has even phoned a couple of times. I know from speaking to people that that's not the norm, but we've felt very very much supported and I've found that I've been quite honest with them as well to be honest." (P22)

When parents did feel supported by medical and social services, this was usually the result of individuals reaching out, rather than a system-wide approach to support.

"'I've also got a caseworker. And she's been fantastic. She's been there if you needed her, she's dropped masks off to me. We've had hand sanitisers dropped off. So she's been an absolute gem. If I ever needed anything, again shopping, she would be more than happy to have done it. So I've had her support well so I've been quite lucky on that" (P6)

Discussion

In addition to the challenges experienced by neurotypical families during the pandemic, parents of children with IDD have also had to manage the distinct challenges related to their children's complex needs. Overall there was extensive variation in parents' reported experiences; some of this variation is likely to reflect the wide range of needs of children in the IMAGINE-ID cohort. Although all children in the cohort have IDD, some children attended mainstream schools and had relatively low levels of support needs, whereas others had multiple physical and behavioural needs.

Parental distress Overall, the parents displayed great resilience, but this often came at the detriment of their own mental health and wellbeing. In line with previous research, we found that caregivers had experienced increased levels of distress [9,29–31,33]. Where children needed high levels of personal care, parents often felt overwhelmed and unsupported, unable to access the respite care or specialised support they normally received. Many described high levels of distress and being close to burn-out, as confirmed by high levels of anxiety in the questionnaire. Evidence from other research also suggests that during the pandemic the parents of children with IDD

For peer review only - http://bmjopen.bmj.com/site/about/guidelines.xhtml

reported higher levels of burnout than the parents of children without disabilities [47] and that the burden of care demands placed carers under increased strain [48].

The findings also confirm previous reports that service provision for children with IDD had reduced or stopped during the pandemic [4,5]. Similar findings were observed in our study, with most parents reporting at least some cancelled medical or social appointments. Social support for the parents in our study was often limited, particularly for lone parents. Many parents felt that online and telephone communication with friends and family did not provide enough support, and several felt isolated as their experience of parents of children with IDD was very different to that of other parents. Parents who reported feeling supported appeared to experience less distress. In a larger quantitative study, Willner and colleagues [31] reported that the parents of people with an IDD received less support than parents of those without IDD, with those caring for people with severe challenging behaviour receiving the least support. This suggests that families who might have benefited most from support were the least likely to receive it.

Short but regular check-ins Regular check-ins with services and schools were described as very helpful, a novel insight that has not, to our knowledge, been reported in previous research. Parents who reported more positive experiences with online schooling tended to report frequent, individualised contact such as phone calls and emails with their children's teachers and support staff, which helped the parent adapt schoolwork for their child. A five-minute telephone call with each family once a week may be more effective than two hours working with the entire class, for children with special educational needs. Even when appointments were cancelled or moved online, frequent communication with services meant parents felt supported during this time of uncertainty. Frequent, brief check-ins are a cheap and easy way to maintain the support available to families during times of crisis, and may reduce the risk of ongoing concerns developing into significant problems.

Unexpected positives Most families were able to draw some positives from the pandemic, as they were able to enjoy spending more time together, as has been previously reported [4,9,33]. Parents also appreciated stepping back from the rush of meetings and appointments, again suggesting that regular check-ins (which might be remote) might suit some families more than having to attend appointments in person.

Impact on children The impact of the pandemic on children's behaviour varied substantially. Some parents reported an increase in behavioural outbursts, while others explained that spending more time at home had led to a decrease in stress due to less demands on social interaction. A study by Neece and colleagues [4] found that "the parents of children with mental and physical disabilities were more likely to report changes in their child's behaviour, such as distractibility, inability to concentrate, irritability and general discomfort." Similar findings were observed in the present study, with several parents reporting that behavioural challenges their children had previously outgrown re-emerged because of changes in routines and lack of support.

Many children were reported to benefit from routines and consistency in their daily lives; these were often disrupted at the start of the lockdown as schools and services closed down and many families started shielding. Where parents were able to introduce new routines, this seemed to benefit children, particularly when these routines allowed children more time at home doing activities they enjoyed. Establishing new routines may be a predictor of positive adjustment in this group. The need for routine has been identified in other research into the impact of the pandemic; lack of routine and or structured support has been associated with frustration for children, as reported by parents [4,33].

Click here to enter text.

For peer review only - http://bmjopen.bmj.com/site/about/guidelines.xhtml

Hardware is a starting point, not a solution Parents highlighted an important difference between the equality and equity of access to digital services. They were often given equality of access to technology, as school provided most families with laptops during the homeschooling period. However, the equity of access often lagged behind, as the resources made available to them in a digital format were not sufficiently tailored to allow their children to engage with them. This compliments the observation by Neece and colleagues [4], that some parents felt ill-equipped to support their child's online learning. Without adaptations, digital homeschooling has the potential to increase inequalities. An individualised approach is essential to understand what is needed, and the best ways to provide support.

Tele-medicine as complementary care delivery Tele-medicine is unlikely to be suitable for all IDD families. In the early stages of the pandemic, many medical and social services closed completely. However, by the time the interviews took place, some were starting to introduce tele-health services to keep in contact with the families in this study. Families enjoyed the flexibility that tele-medicine gave them, reducing the need to travel to appointments with children. However, there was a real sense that tele-health was not an appropriate substitute for in-person assessments, particular for children with IDD. Parents felt that they were not able to communicate their children's often complex needs over the phone or email, and the children themselves often struggled to engage with medical professionals who they couldn't see in front of them. These issues have been previously raised with regards to telehealth services provided during the pandemic, with the need for adaptation highlighted [49,50]. While tele-medicine offers many opportunities to expand services, it is important to emphasise that this new mode of healthcare provision will not suit everyone, and families of children with IDD should be offered a range of options to ensure their child receives appropriate support. Reflecting a broader shift to digital services across healthcare provision, blended care will likely be the most appropriate approach going forward.

Strengths, limitations and future directions

The study reflected the experiences of the mothers that were interviewed. Clearly, this small study cannot represent the full extent of the impact of the first UK lockdown on the IDD community. Our use of consecutive sampling was necessitated by the approaching end of shielding in the UK in August 2020; we felt it was important to interview all participants under the same level of restrictions. However, this also means that we were not able to selectively recruit participants, and so it is unclear to what extent variations in experience are related to variations in child's level of need, or additional factors. Our interviewees were all female, and were mostly white and in co-parenting relationships; experiences are likely to differ for non-white, non-female, single parents.

Concurrent large-scale questionnaire-based studies, such as those conducted by Willner and colleagues [31], are needed to address this. Small-scale qualitative studies can identify broader experiences than might be reported in large-scale quantitative studies, and can produce novel hypotheses for further examination. However, a key limitation of our methodology is its lack of generalisability outside of the first UK lockdown, to other countries or timepoints with different levels of infection and restrictions. We also did not interview any children or young people with IDD, and therefore our findings are reliant on parents' perceptions of their child's experience, rather than first-hand accounts. Future research should seek to explore the experiences of children with IDD through their own communication, as there are undoubtedly features which have been missed in the present study.

The interviews contained a few notable absences. We had anticipated reports of excessive hand-washing and cleanliness, however this was rarely mentioned in our interviews. We had also expected some families to express concern about being judged for needing allowances/exemptions, such as their child not wearing a mask when out in public, but this was rarely discussed. No one reported breaking the rules. Although this could be attributed to expectancy biases, we interpret

For peer review only - http://bmjopen.bmj.com/site/about/guidelines.xhtml

this to be representative of the high national compliance during the first UK lockdown, particularly in individuals with pre-existing medical conditions [51]. Research by Rogers et al. [33] with the mothers of IDD children highlighted themes of stigma and powerlessness, which were not as prominent in our interviews.

The views presented here reflect families' experiences in the first few months of the pandemic and the first UK lockdown, a time of particularly heightened anxieties and stringent restrictions. At the time of writing the manuscript the government's response to the pandemic continues to evolve monthly. It is likely that parents' experiences and attitudes have changed over time. Therefore, we plan to conduct follow-up interviews in the spring of 2021, which may offer different insights as families have had more time to adapt to the 'new normal'. This will also allow us to explore concerns about how children with IDD have navigated the changes in rules [35-25] and explore the impact of a prolonged national crisis on the carers of children with IDD.

Conclusion

Our findings confirm observations previously found in UK parents of children with IDD [33] and provide new insights on the use of technology during the pandemic for schooling and healthcare, as well as the need for regular check-ins. Parents of children with IDD experienced many challenges during the UK Covid-19 outbreak during the spring/summer of 2020. In addition to the demands of home-schooling, concern over infection, and lack of social connection that families across the country dealt with, families in this study had to meet the additional and often complex educational and medical needs of their children. Many parents felt let down and forgotten by medical and school services, and felt isolated from the rest of the country. However, parents also used their experience to develop coping strategies and found some unexpected benefits for their children, such as spending more time together at home. We can learn from these parents' experiences to provide guidelines for the future, to ensure these families are not forgotten.

Funding statement: The IMAGINE-ID study is funded was funded by the Medical Research Council and Medical Research Foundation (MR/L011166/1 and MR/N022572/1). Research conducted at the CRF UCL GOS ICH is supported by the NIHR. The funders played no role in the design of the study and collection, analysis, interpretation of data or writing of the manuscript.

Competing interests statement: The authors declare that they have no competing interests.

Data availability statement: The datasets generated and/or analysed during the current study are not publicly available in order to protect the anonymity of those taking part, but are available from the corresponding author on reasonable request.

Author statement: JW, LH and DS designed and directed the study. JW and LH conducted the interviews and analysis (theme development and review). Themes were reviewed following several detailed discussions with the other members of the research team DS and WM. Transcripts were coded by independent reviewers LW and TA. The IMAGINE-ID consortium contributed to recruitment and manuscript revisions (see Supplemental materials).

Acknowledgements: We would like to thank all the families who took part in this study and the IMAGINE-ID consortium for making this research possible.

Word count 7,479 (excluding boxes, tables, figures and references)

For peer review only - http://bmjopen.bmj.com/site/about/guidelines.xhtml

References

1 Maulik PK, Mascarenhas MN, Mathers CD, *et al.* Prevalence of intellectual disability: A meta-analysis of population-based studies. Res. Dev. Disabil. 2011;**32**:419–36. doi:10.1016/j.ridd.2010.12.018

2 American Association on Intellectual and Developmental Disabilities. *Intellectual disability : definition, classification, and systems of supports*. American Association on Intellectual and Developmental Disabilities 2010.

3 Gilissen C, Hehir-Kwa JY, Thung DT, *et al.* Genome sequencing identifies major causes of severe intellectual disability. *Nature* 2014;**511**:344–7. doi:10.1038/nature13394

4 Neece C, McIntyre LL, Fenning R. Examining the impact of COVID-19 in ethnically diverse families with young children with intellectual and developmental disabilities. *J Intellect Disabil Res* 2020;**64**:739–49. doi:10.1111/jir.12769

5 Eshraghi AA, Li C, Alessandri M, *et al.* COVID-19: overcoming the challenges faced by individuals with autism and their families. *The Lancet Psychiatry* 2020;**7**:481–3. doi:https://doi.org/10.1016/S2215-0366(20)30197-8

6 National Autistic Society. The impact of coronavirus on autistic people and their families in the UK. 2020.

7 Cullen W, Gulati G, Kelly BD. Mental health in the COVID-19 pandemic. *QJM An Int J Med* 2020;**113**:311–2. doi:10.1093/QJMED/HCAA110

8 Gulati G, Fistein E, Dunne CP, *et al.* People with Intellectual Disabilities and the COVID-19 Pandemic. *Ir J Psychol Med* 2020;:1–2. doi:10.1017/ipm.2020.66

9 Rose J, Willner P, Cooper V, *et al.* The effect on and experience of families with a member who has Intellectual and Developmental Disabilities of the COVID-19 pandemic in the UK: developing an investigation. *Int J Dev Disabil* 2020;:1–3. doi:10.1080/20473869.2020.1764257

10 Holmes EA, O'Connor RC, Perry VH, *et al.* Multidisciplinary research priorities for the COVID-19 pandemic: a call for action for mental health science. The Lancet Psychiatry. 2020;**7**:547–60. doi:10.1016/S2215-0366(20)30168-1

11 Dekker MC, Koot HM, Van Der Ende J, *et al.* Emotional and behavioral problems in children and adolescents with and without intellectual disability. *J Child Psychol Psychiatry Allied Discip* 2002;**43**:1087–98. doi:10.1111/1469-7610.00235

12 Dykens EM. Annotation: Psychopathology in Children with Intellectual Disability. *J Child Psychol Psychiatry* 2000;**41**:407–17. doi:10.1111/1469-7610.00626

13 Einfeld S, Tonge B, Turner G, *et al.* Longitudinal course of behavioural and emotional problems of young persons with Prader-Willi, Fragile X, Williams and Down syndromes. *J Intellect Dev Disabil* 1999;**24**:349–54. doi:10.1080/13668259900034111

14 Totsika V, Hastings RP. Persistent challenging behaviour in people with an intellectual disability. *Curr Opin Psychiatry* 2009;**22**:437–41. doi:10.1097/YCO.0b013e32832cd9b8

15 Tonge B, Einfeld S. The trajectory of psychiatric disorders in young people with intellectual disabilities. *Aust N Z J Psychiatry* 2000;**34**:80–4. doi:10.1046/j.1440-1614.2000.00695.x

Click here to enter text.

For peer review only

16 Tonge BJ, Einfeld SL. Psychopathology and intellectual disability: The Australian Child to Adult Longitudinal Study. In: Glidden LM, ed. . London: : Academic Press 2003. 61–91.

17 Emerson E, Hatton C. Mental health of children and adolescents with intellectual disabilities in Britain. *Br J Psychiatry* 2007;**191**:493–9. doi:10.1192/bjp.bp.107.038729

18 Emerson E, Einfeld S. Emotional and behavioural difficulties in young children with and without developmental delay: a bi-national perspective. *J Child Psychol Psychiatry* 2010;**51**:583–93. doi:10.1111/j.1469-7610.2009.02179.x

19 Emerson E, McCulloch A, Graham H, *et al.* Socioeconomic circumstances and risk of psychiatric disorders among parents of children with early cognitive delay. *Am J Intellect Dev Disabil* 2010;**115**:30–42. doi:10.1352/1944-7558-115.1.30

20 Emerson E, Shahtahmasebi S, Lancaster G, *et al.* Poverty transitions among families supporting a child with intellectual disability. *J Intellect Dev Disabil* 2010;**35**:224–34. doi:10.3109/13668250.2010.518562

21 Shahtahmasebi S, Emerson E, Berridge D, *et al.* Child disability and the dynamics of family poverty, hardship and financial strain: Evidence from the UK. *J Soc Policy* 2011;**40**:653–73. doi:10.1017/S0047279410000905

22 Mugno D, Ruta L, D'Arrigo VG, *et al.* Impairment of quality of life in parents of children and adolescents with pervasive developmental disorder. *Health Qual Life Outcomes* 2007;**5**:22. doi:10.1186/1477-7525-5-22

23 Baker BL, McIntyre LL, Blacher J, *et al.* Pre-school children with and without developmental delay: Behaviour problems and parenting stress over time. *J Intellect Disabil Res* 2003;**47**:217–30. doi:10.1046/j.1365-2788.2003.00484.x

24 Gray KM, Piccinin AM, Hofer SM, *et al.* The longitudinal relationship between behavior and emotional disturbance in young people with intellectual disability and maternal mental health. *Res Dev Disabil* 2011;**32**:1194–204. doi:10.1016/j.ridd.2010.12.044

25 Emerson E, Robertson J, Wood J. Levels of Psychological Distress Experienced by Family Carers of Children and Adolescents with Intellectual Disabilities in an Urban Conurbation. *J Appl Res Intellect Disabil* 2004;**17**:77–84. doi:10.1111/j.1360-2322.2004.00183.x

26 Olsson MB, Hwang CP. Depression in mothers and fathers of children with intellectual disability. *J Intellect Disabil Res* 2001;**45**:535–43. doi:10.1046/j.1365-2788.2001.00372.x

27 White N, Hastings RP. Social and professional support for parents of adolescents with severe intellectual disabilities. *J Appl Res Intellect Disabil* 2004;**17**:181–90. doi:10.1111/j.1468-3148.2004.00197.x

28 Marquis S, Hayes M V., McGrail K. Factors Affecting the Health of Caregivers of Children Who Have an Intellectual/Developmental Disability. *J Policy Pract Intellect Disabil* 2019;**16**:201–16. doi:10.1111/jppi.12283

29 Bellomo TR, Prasad S, Munzer T, *et al.* The impact of the COVID-19 pandemic on children with autism spectrum disorders. *J Pediatr Rehabil Med* 2020;**Preprint**:1–6. doi:10.3233/PRM-200740

30 Colizzi M, Sironi E, Antonini F, *et al.* Psychosocial and behavioral impact of COVID-19 in autism spectrum disorder: An online parent survey. *Brain Sci* 2020;**10**. doi:10.3390/brainsci10060341

31 Willner P, Rose J, Stenfert Kroese B, *et al.* Effect of the COVID-19 pandemic on the mental

For peer review only

For peer review only - http://bmjopen.bmj.com/site/about/guidelines.xhtml

health of carers of people with intellectual disabilities. *J Appl Res Intellect Disabil* 2020;**n/a**. doi:10.1111/jar.12811

32 Couglan S. Coronavirus: Parents of disabled children ‘cut off and ignored’. BBC News. 2020.www.bbc.co.uk/news/education-52806105. Published May 27.

33 Rogers G, Perez-Olivas G, Stenfert Kroese B, *et al.* The experiences of mothers of children and young people with intellectual disabilities during the first COVID-19 lockdown period. *J Appl Res Intellect Disabil* 2021;:1–10. doi:10.1111/jar.12884

34 Westrupp EM, Stokes MA, Fuller-Tyszkiewicz M, *et al.* Subjective wellbeing in parents during the COVID-19 pandemic in Australia. *J Psychosom Res* 2021;**145**:110482. doi:10.1016/j.jpsychores.2021.110482

35 Asbury K, Fox L, Deniz E, *et al.* How is COVID-19 Affecting the Mental Health of Children with Special Educational Needs and Disabilities and Their Families? *J Autism Dev Disord* 2020;**51**:1772–80. doi:10.1007/s10803-020-04577-2

36 Cacioppo M, Bouvier S, Bailly R, *et al.* Emerging health challenges for children with physical disabilities and their parents during the COVID-19 pandemic : The ECHO French survey. *Ann Phys Rehabil Med* 2020.

37 Grumi S, Provenzi L, Gardani A, *et al.* Rehabilitation services lockdown during the COVID-19 emergency: the mental health response of caregivers of children with neurodevelopmental disabilities. *Disabil Rehabil* 2020;**43**:27–32. doi:10.1080/09638288.2020.1842520

38 Harris PA, Taylor R, Thielke R, *et al.* Research electronic data capture (REDCap)-A metadata-driven methodology and workflow process for providing translational research informatics support. *J Biomed Inform* 2009;**42**:377–81. doi:10.1016/j.jbi.2008.08.010

39 Nikolaidis A, Paksarian D, Alexander L, *et al.* The Coronavirus Health and Impact Survey (CRISIS) reveals reproducible correlates of pandemic-related mood states across the Atlantic. *medRxiv Prepr Serv Heal Sci* Published Online First: 27 August 2020. doi:10.1101/2020.08.24.20181123

40 Kushllck A, Blunden R, Cox G. A method of rating behaviour characteristics for use in large scale surveys of mental handicap. *Psychol Med* 1973;**3**:466–78. doi:10.1017/S0033291700054271

41 Goodman A, Lamping DL, Ploubidis GB. When to Use Broader Internalising and Externalising Subscales Instead of the Hypothesised Five Subscales on the Strengths and Difficulties Questionnaire (SDQ): Data from British Parents, Teachers and Children. *J Abnorm Child Psychol* 2010;**38**:1179–91. doi:10.1007/s10802-010-9434-x

42 Zigmond AS, Snaith RP. The Hospital Anxiety and Depression Scale. *Acta Psychiatr Scand* 1983;**67**:361–70. doi:10.1111/j.1600-0447.1983.tb09716.x

43 Hastings RP. Parental stress and behaviour problems of children with developmental disability. J. Intellect. Dev. Disabil. 2002;**27**:149–60. doi:10.1080/1366825021000008657

44 Braun V, Clarke V. Using thematic analysis in psychology. *Qual Res Psychol* 2006;**3**:77–101.

45 Braun V, Clarke V. *Successful Qualitative Research: A practical Guide for Beginners*. SAGE Publications 2013.

46 Braun V, Clarke V. Reflecting on reflexive thematic analysis. *Qual Res Sport Exerc Heal* 2019;**11**:589–97. doi:10.1080/2159676X.2019.1628806

Click here to enter text.

For peer review only - http://bmjopen.bmj.com/site/about/guidelines.xhtml

47 Fontanesi L, Marchetti D, Mazza C, *et al.* The Effect of the COVID-19 Lockdown on Parents: A Call to Adopt Urgent Measures. *Psychol Trauma Theory, Res Pract Policy* 2020;**12**:79–81. doi:10.1037/tra0000672

48 Alexander R, Ravi A, Barclay H, *et al.* Guidance for the Treatment and Management of COVID-19 Among People with Intellectual Disabilities. *J Policy Pract Intellect Disabil* 2020;**17**:256–69. doi:10.1111/jppi.12352

49 Jeste S, Hyde C, Distefano C, *et al.* Changes in access to educational and healthcare services for individuals with intellectual and developmental disabilities during COVID-19 restrictions. *J Intellect Disabil Res* 2020;**64**:825–33. doi:10.1111/jir.12776

50 Provenzi L, Grumi S, Borgatti R. Alone With the Kids: Tele-Medicine for Children With Special Healthcare Needs During COVID-19 Emergency. *Front Psychol* 2020;**11**:1–6. doi:10.3389/fpsyg.2020.02193

51 Krekel C, Swanke S, De Neve J-E, *et al.* Are Happier People More Compliant? Global Evidence From Three Large-Scale Surveys During Covid-19 Lockdowns. 2020. www.iza.org (accessed 5 Jan 2021).

For peer review only

Table 1: Participant characteristics

Click here to enter text.

SNV: Single Nucleotide variant; CNV: Copy Number Variant; SCA: Sex Chromosome Aneuploidy; M: Male; F: Female; ASD: Autism Spectrum Disorder; ADHD: Attention deficit Hyperactivity Disorder

**Theme**	**Sub-theme**
Managing pre-existing challenges, in a time of increased strain for everyone “you just feel like our life was so different to other people’s”	“We’ve been in lockdown since he was born” Social distancing is the norm
	Left behind: “I feel like they just left people who are vulnerable behind for two months.”
	Planning for complex needs “there’s a lot more things that I needed to do than the average sort of family”<br>• Fewer resources, but behavioural issues are the same<br>• Explaining covid
Mixed emotions around the challenges and unexpected benefits of lockdown: “The pandemic was nice but really hard”	Desperate for hugs: “I don’t like lockdown because I want snuggles with my nanny”
	Happy at home: “Everyone’s keeping distance from me and that’s how I like it”
	Strained relationships: “Being constantly 24/7 together definitely did build up pressure”
	Spending time together and slowing down: “It brought us a lot closer together”
Support matters	Transition to telehealth “I don’t think you can replace face to face with a telephone”<br>Considering equality and equity in remote schooling<br>• Equality through access to technology: “I think the first thing to do is make sure everyone is able to access what they’re providing”<br>• Equity of access lagging behind as adaptations aren’t always appropriate
	Considering equality and equity in remote schooling<br>• Equality through access to technology: “I think the first thing to do is make sure everyone is able to access what they’re providing”<br>• Equity of access lagging behind as adaptations aren’t always appropriate
	Checking in: “just being able to have that support bubble, rather than being locked in your own four walls”

Table 2: Summary of themes and sub-themes

Click here to enter text.

For peer review only - http://bmjopen.bmj.com/site/about/guidelines.xhtml

Supplemental materials

Interview schedule

1. How do you feel the coronavirus pandemic is affecting you and your family, if at all? It may be helpful to consider your response in terms of routine, appointments, respite care, support networks, visiting family, childcare arrangements, work and related thoughts and feelings.

Prompts:

   a. Can you tell me a bit more about the coronavirus pandemic has changed your and your child's:
      i. Day to day life?
      ii. Childcare?
      iii. Access to services? Have any appointments been re-arranged, taken place online or been cancelled/postponed? How easy have you found accessing online/telephone appointments?
      iv. Seeing friends and family?
      v. Access to support networks?

2. How do you feel the coronavirus pandemic is affecting your ability to cope?

3. How do you feel the coronavirus pandemic is affecting your parenting skills?

4. How are you getting along as a family? / How is everyone getting along together?

5. How do you feel the physical (social) distancing and lockdown/isolation measures have affected you and your child/children during the coronavirus pandemic, if at all?
If this is affecting you, can you explain or list in what ways?
For example, consider the impact on your support network, social activities, social media and online socialising, and general contact with family, friends and services etc.

6. How do you feel that the easing of lockdown measures/restrictions are affecting you and your child/children, if at all? If this is affecting you, can you explain or list in what ways? For example, consider the impact on your support network, social activities, social media and online socialising, and general contact with family, friends and services etc.

7. In an ideal world, what resources or allowances would you need to manage lockdown/ to help your child?

8. Are there any coping strategies that you are using during this coronavirus pandemic? If so, what are these coping strategies and how have they helped you?

9. Have there been any unexpected positives during the pandemic? Is there anything from the lockdown that you would like to carry forward into everyday life for you/your child?

10. Is there anything else about your experiences during this pandemic that you would like to mention?

S2 Genetic variants of participants

For peer review only - http://bmjopen.bmj.com/site/about/guidelines.xhtml

Variant Type	Variants
Aneuploidy	48,XXYY
Aneuploidy	45,X
CNV	chr17p11.2 Duplication
CNV	chr18q12.3 Triplication
CNV	chr3q26.33-q27.1 Deletion
Aneuploidy	48,XXYY
CNV	chr7q11.23 Deletion
CNV	chr10q22.3-q23.2 Deletion
CNV	chr5q31.3 Duplication & chrXp22.1 Deletion
CNV	chr1q21.1-q21.2 Deletion
SNV	chr20p13 Missense & chrXq25 Missense
SNV	chrXp22.12 Splice site
CNV	chr16p13.11-p12.3 Duplication & chr7p21.1 Deletion
SNV	chr2q24.3 Missense
CNV	chr5q14.3 Deletion
SNV	chrXq26.1 Missense
CNV	chr16p13.11 Deletion
CNV	chr13q33.2-q33.3 Deletion
CNV	chr18p11.32-p11.21 & chrXp22.12 Duplication
CNV	chr7p21.1 Deletion
CNV	chr15q11.1-q13.1 Mosaic duplication
CNV	chr16p13.11-p12.3 Duplication
CNV	chr16p13.11 Deletion

Table S1: Genetic variants of IMAGINE-ID children with IDD

For peer review only - http://bmjopen.bmj.com/site/about/guidelines.xhtml

Supplementary Materials

IMAGINE-ID Consortium Membership

[1] School of Clinical Medicine, University of Cambridge, Cambridge Biomedical Campus, Cambridge, UK

[2] Medical Research Council Centre for Neuropsychiatric Genetics and Genomics, Division of Psychological Medicine and Clinical Neurosciences, and Neuroscience and Mental Health Research Institute, Cardiff University, Cardiff, UK.

[3] NIHR BRC Great Ormond Street Institute of Child Health, University College London, London, UK

[4] Unique – The Rare Chromosome Disorder Support Group, London, UK.

Title (if applicable)	First Name	Surname	Institution
Dr	Kate	Baker	1
	Eleanor	Dewhurst	1
	Amy	Lafont	1
Professor	F Lucy	Raymond	1
	Terry	Shirley	1
	Hayley	Tilley	1
	Husne	Timur	1
	Catherine	Titterton	1
	Neil	Walker	1
	Sarah	Wallwork	1
	Francesca	Wicks	1
Dr	Zheng	Ye	1
	Marie	Erwood	1
	Sophie	Andrews	2
	Philippa	Birch	2
	Samantha	Bowen	2
	Karen	Bradley	2
	Aimee	Challenger	2
Dr	Samuel	Chawner	2
Dr	Andrew	Cuthbert	2
Professor	Jeremy	Hall	2
Professor	Peter	Holmans	2
	Sarah	Law	2
	Nicola	Lewis	2
	Sinead	Morrison	2
	Hayley	Moss	2
Professor Sir	Michael	Owen	2
	Sinead	Ray	2
	Matthew	Sopp	2
	Molly	Tong	2
Professor	Marianne	van den Bree	2
	Nadia	Coscini	3

For peer review only

	Sarah	Davies	3
	Spiros	Denaxas	3
	Hayley	Denyer	3
	Nasrtullah	Fatih	3
	Manoj	Juj	3
	Ellie	Kerry	3
	Anna	Lucock	3
Dr	William	Mandy	3
	Frida	Printzlau	3
Professor	David	Skuse	3
Dr	Ramya	Srinivasan	3
Dr	Susan	Walker	3
	Alice	Watkins	3
Dr	Jeanne	Wolstencroft	3
Dr	Beverly	Searle	4
Dr	Anna	Pelling	4

IMAGINE-ID Clinical Collaborators

**Title (if applicable)**	**First Name**	**Surname**	**Shorthand Institution**	**Genetics service**	**Hospital Trust**
Dr	John	Dean	Aberdeen	Aberdeen Royal Infirmary Genetics Service	NHS GRAMPIAN
Dr	Lisa	Robertson	Aberdeen	Aberdeen Royal Infirmary Genetics Service	NHS GRAMPIAN
Dr	Denise	Williams	Birmingham	West Midlands Regional Genetics Service	BIRMINGHAM WOMEN'S NHS FOUNDATION TRUST
Dr	Alan	Donaldson	Bristol	Bristol Clinical Genetics Service	UNIVERSITY HOSPITALS BRISTOL NHS FOUNDATION TRUST
Professor	Lucy	Raymond	Cambridge	East Anglian Medical Genetics Service	CAMBRIDGE UNIVERSITY HOSPITALS NHS FOUNDATION TRUST
Dr	Annie	Procter	Cardiff	All Wales Regional Genetics Service	CARDIFF AND VALE UNIVERSITY LHB
Dr	Jonathan	Berg	Dundee	Ninewells Hospital Dundee Genetics Service	NHS TAYSIDE

For peer review only

	Yanick	Crow	Edinburgh	Western General Hospital Edinburgh Genetics Service	NHS LOTHIAN
Professor	Anne	Lampe	Edinburgh	Western General Hospital Edinburgh Genetics Service	NHS LOTHIAN
Dr	Julia	Rankin	Exeter	Peninsula Genetics Service	ROYAL DEVON AND EXETER NHS FOUNDATION TRUST
Dr	Shelagh	Joss	Glasgow	Glasgow Genetics Centre	NHS GREATER GLASGOW & CLYDE
Professor	Lyn	Chitty	GOSH	London North East Thames Regional Genetics Service - Clinical Genetics	GREAT ORMOND STREET HOSPITAL FOR CHILDREN NHS FOUNDATION TRUST
Professor	Frances	Flinter	Guy's	London Guy's Hospital Genetic Centre	GUY'S AND ST THOMAS' NHS FOUNDATION TRUST
Dr	Muriel	Holder	Guy's	London Guy's Hospital Genetic Centre	GUY'S AND ST THOMAS' NHS FOUNDATION TRUST
Dr	Alison	Kraus	Leeds	Yorkshire Regional Genetics Service - Clinical Genetics	LEEDS TEACHING HOSPITALS NHS TRUST
Dr	Julian	Barwell	Leicester	Leicestershire Genetics Centre	UNIVERSITY HOSPITALS OF LEICESTER NHS TRUST
Dr	Pradeep	Vasudevan	Leicester	Leicestershire Genetics Centre	UNIVERSITY HOSPITALS OF LEICESTER NHS TRUST
Dr	Astrid	Weber	Liverpool	Cheshire & Merseyside Regional Genetic Service	LIVERPOOL WOMEN'S NHS FOUNDATION TRUST

For peer review only

Dr	William	Newman	Manchester	Manchester Centre for Genomic Medicine	CENTRAL MANCHESTER UNIVERSITY HOSPITALS NHS FOUNDATION TRUST
Dr	Miranda	Splitt	Newcastle	Northern Genetics Service	THE NEWCASTLE UPON TYNE HOSPITALS NHS FOUNDATION TRUST
Dr	Virginia	Clowes	North West Thames	London North West Thames Regional Genetics Service	NORTH WEST LONDON HOSPITALS NHS TRUST
Dr	Fleur	van Dijk	North West Thames	London North West Thames Regional Genetics Service	NORTH WEST LONDON HOSPITALS NHS TRUST
Dr	Rachel	Harrison	Nottingham	Nottingham Regional Genetics Service	NOTTINGHAM UNIVERSITY HOSPITALS NHS TRUST
Dr	Usha	Kini	Oxford	Oxford Genetics Service	OXFORD UNIVERSITY HOSPITALS NHS TRUST
Dr	Oliver	Quarrell	Sheffield	Sheffield Genetic Services	SHEFFIELD CHILDREN'S NHS FOUNDATION TRUST
Dr	Diana	Baralle	Southampton	Wessex Clinical Genetics Service	UNIVERSITY HOSPITAL SOUTHAMPTON NHS FOUNDATION TRUST
Dr	Sahar	Mansour	St George's	London South West Thames Regional Genetics Service	ST GEORGE'S HEALTHCARE NHS FOUNDATION TRUST

For peer review only

STROBE Statement—checklist of items that should be included in reports of observational studies

	Item No	Recommendation	Page No
**Title and abstract**	1	(*a*) Indicate the study's design with a commonly used term in the title or the abstract	1
		(*b*) Provide in the abstract an informative and balanced summary of what was done and what was found	2
**Introduction**			
Background/rationale	2	Explain the scientific background and rationale for the investigation being reported	3
Objectives	3	State specific objectives, including any prespecified hypotheses	3-4
**Methods**			
Study design	4	Present key elements of study design early in the paper	4
Setting	5	Describe the setting, locations, and relevant dates, including periods of recruitment, exposure, follow-up, and data collection	4
Participants	6	(*a*) *Cohort study*—Give the eligibility criteria, and the sources and methods of selection of participants. Describe methods of follow-up<br>*Case-control study*—Give the eligibility criteria, and the sources and methods of case ascertainment and control selection. Give the rationale for the choice of cases and controls<br>*Cross-sectional study*—Give the eligibility criteria, and the sources and methods of selection of participants	4
		(*b*) *Cohort study*—For matched studies, give matching criteria and number of exposed and unexposed<br>*Case-control study*—For matched studies, give matching criteria and the number of controls per case	N/A
Variables	7	Clearly define all outcomes, exposures, predictors, potential confounders, and effect modifiers. Give diagnostic criteria, if applicable	4-5
Data sources/ measurement	8*	For each variable of interest, give sources of data and details of methods of assessment (measurement). Describe comparability of assessment methods if there is more than one group	*4*
Bias	9	Describe any efforts to address potential sources of bias	5
Study size	10	Explain how the study size was arrived at	4
Quantitative variables	11	Explain how quantitative variables were handled in the analyses. If applicable, describe which groupings were chosen and why	N/A Descriptive statistics
Statistical methods	12	(*a*) Describe all statistical methods, including those used to control for confounding	N/A
		(*b*) Describe any methods used to examine subgroups and interactions	N/A
		(*c*) Explain how missing data were addressed	N/A
		(*d*) *Cohort study*—If applicable, explain how loss to follow-up was addressed<br>*Case-control study*—If applicable, explain how matching of cases and controls was addressed<br>*Cross-sectional study*—If applicable, describe analytical methods taking account of sampling strategy	N/A
		(*e*) Describe any sensitivity analyses	N/A

Continued on next page

For peer review only - http://bmjopen.bmj.com/site/about/guidelines.xhtml

**Results**			
Participants	13*	(a) Report numbers of individuals at each stage of study—eg numbers potentially eligible, examined for eligibility, confirmed eligible, included in the study, completing follow-up, and analysed	5
		(b) Give reasons for non-participation at each stage	N/A
		(c) Consider use of a flow diagram	N/A
Descriptive data	14*	(a) Give characteristics of study participants (eg demographic, clinical, social) and information on exposures and potential confounders	5
		(b) Indicate number of participants with missing data for each variable of interest	5
		(c) *Cohort study*—Summarise follow-up time (eg, average and total amount)	N/A
Outcome data	15*	*Cohort study*—Report numbers of outcome events or summary measures over time	*5-6*
		Case-control study—Report numbers in each exposure category, or summary measures of exposure	N/A
		Cross-sectional study—Report numbers of outcome events or summary measures	N/A
Main results	16	(*a*) Give unadjusted estimates and, if applicable, confounder-adjusted estimates and their precision (eg, 95% confidence interval). Make clear which confounders were adjusted for and why they were included	N/A
		(*b*) Report category boundaries when continuous variables were categorized	N/A
		(*c*) If relevant, consider translating estimates of relative risk into absolute risk for a meaningful time period	N/A
Other analyses	17	Report other analyses done—eg analyses of subgroups and interactions, and sensitivity analyses	N/A
**Discussion**			
Key results	18	Summarise key results with reference to study objectives	12
Limitations	19	Discuss limitations of the study, taking into account sources of potential bias or imprecision. Discuss both direction and magnitude of any potential bias	14-15
Interpretation	20	Give a cautious overall interpretation of results considering objectives, limitations, multiplicity of analyses, results from similar studies, and other relevant evidence	12-15
Generalisability	21	Discuss the generalisability (external validity) of the study results	14
**Other information**			
Funding	22	Give the source of funding and the role of the funders for the present study and, if applicable, for the original study on which the present article is based	15

*Give information separately for cases and controls in case-control studies and, if applicable, for exposed and unexposed groups in cohort and cross-sectional studies.

Note: An Explanation and Elaboration article discusses each checklist item and gives methodological background and published examples of transparent reporting. The STROBE checklist is best used in conjunction with this article (freely available on the Web sites of PLoS Medicine at http://www.plosmedicine.org/, Annals of Internal Medicine at http://www.annals.org/, and Epidemiology at http://www.epidem.com/). Information on the STROBE Initiative is available at www.strobe-statement.org.

For peer review only

For peer review only - http://bmjopen.bmj.com/site/about/guidelines.xhtml

Standards for Reporting Qualitative Research (SRQR) Table

No.	Topic	Page/line reference
S1	Title	Page 1, Line 1
S2	Abstract	Page 2, Lines 2-14
S3	Problem formulation	See entire Introduction section (Pages 3 – 4)
S4	Research question	Page 3, Line 42 – Page 4, Line 2
S5	Qualitative approach and research paradigm	Page 5, Lines 10-26
S6	Researcher characteristics and reflexivity	Page 5, Lines 15-21
S7	Context	Page 4, Lines 5-16
S8	Sampling strategy	Page 4, Lines 9-10
S9	Ethical issues	Page 4, Lines 15-16
S10	Data collection methods	See entire Methods section (pages 4 – 5)
S11	Instruments	Page 4, Line 22 – Page 5, Line 8
S12	Units of study	Page 5, Lines 30 – 19
S13	Data processing	Page 5, Lines 10 – 26
S14	Data analysis	Page 5, Lines 10 – 26
S15	Techniques to enhance trustworthiness	Page 5, Lines 10 – 26
S16	Synthesis and interpretation	See entire Results section (pages 5 – 12)
S17	Links to empirical data	See entire Results section (Pages 5 – 12)
S18	Integration with prior work, implications, transferability, and contributions to the field	See entire Discussion section (Pages 12 – 15)
S19	Limitations	Page 14, Line 26 – Page 5, Line 12
S20	Conflicts of interest	Page 15, Line 31
S21	Funding	Page 15, Lines 27 - 30

For peer review only

"We've been in lockdown since he was born": A mixed-methods exploration of the experiences of families caring for children with intellectual disability during the Covid-19 pandemic in the UK

Journal:	*BMJ Open*
Manuscript ID	bmjopen-2021-049386.R2
Article Type:	Original research
Date Submitted by the Author:	20-Aug-2021
Complete List of Authors:	Wolstencroft, Jeanne; University College London<br>Hull, Laura; University College London<br>Warner, Lauren; University College London<br>Akhtar, Tooba Nadeem; University College London<br>Mandy, W; University College London<br>IMAGINE, ID<br>Skuse, David ; University College London, Institute of Child Health
**Primary Subject Heading**:	Mental health
Secondary Subject Heading:	Paediatrics
Keywords:	COVID-19, MENTAL HEALTH, PAEDIATRICS, PSYCHIATRY

I, the Submitting Author has the right to grant and does grant on behalf of all authors of the Work (as defined in the below author licence), an exclusive licence and/or a non-exclusive licence for contributions from authors who are: i) UK Crown employees; ii) where BMJ has agreed a CC-BY licence shall apply, and/or iii) in accordance with the terms applicable for US Federal Government officers or employees acting as part of their official duties; on a worldwide, perpetual, irrevocable, royalty-free basis to BMJ Publishing Group Ltd ("BMJ") its licensees and where the relevant Journal is co-owned by BMJ to the co-owners of the Journal, to publish the Work in this journal and any other BMJ products and to exploit all rights, as set out in our licence.

The Submitting Author accepts and understands that any supply made under these terms is made by BMJ to the Submitting Author unless you are acting as an employee on behalf of your employer or a postgraduate student of an affiliated institution which is paying any applicable article publishing charge ("APC") for Open Access articles. Where the Submitting Author wishes to make the Work available on an Open Access basis (and intends to pay the relevant APC), the terms of reuse of such Open Access shall be governed by a Creative Commons licence – details of these licences and which Creative Commons licence will apply to this Work are set out in our licence referred to above.

Other than as permitted in any relevant BMJ Author's Self Archiving Policies, I confirm this Work has not been accepted for publication elsewhere, is not being considered for publication elsewhere and does not duplicate material already published. I confirm all authors consent to publication of this Work and authorise the granting of this licence.

For peer review only

"We've been in lockdown since he was born": A mixed-methods exploration of the experiences of families caring for children with intellectual disability during the Covid-19 pandemic in the UK

Name of Authors
Jeanne Wolstencroft
Laura Hull
Lauren Warner
Tooba Akhtar
William Mandy
IMAGINE-ID consortium
David Skuse

Affiliations
Jeanne Wolstencroft (corresponding author), The Great Ormond Street Institute of Child Health, University College London, 30 Guilford Street, London, WC1N 1EH

Laura Hull, Division of Psychology and Language Sciences, Faculty of Brain Sciences, University College London, 26 Bedford Way, London, WC1H 0AP

Lauren Warner, The Great Ormond Street Institute of Child Health, University College London, 30 Guilford Street, London, WC1N 1EH

Tooba Akhtar, The Great Ormond Street Institute of Child Health, University College London, 30 Guilford Street, London, WC1N 1EH

William Mandy, Division of Psychology and Language Sciences, Faculty of Brain Sciences, University College London, 26 Bedford Way, London, WC1H 0AP

IMAGINE-ID consortium (see Imagine ID Consortium Membership list)

David Skuse, The Great Ormond Street Institute of Child Health, University College London, 30 Guilford Street, London, WC1N 1EH

Corresponding address and email
Jeanne Wolstencroft (corresponding author)
j.wolstencroft@ucl.ac.uk
The Great Ormond Street Institute of Child Health
University College London
30 Guilford Street
London, WC1N 1EH
0207 905 2168

Click here to enter text.

For peer review only - http://bmjopen.bmj.com/site/about/guidelines.xhtml

Abstract

Objectives: This study aimed to explore the experiences of parents caring for children with intellectual and developmental disability (IDD) during the UK national lockdown in spring 2020, resulting from the Covid-19 pandemic. Design: Participants were identified using opportunity sampling from the IMAGINE-ID national (UK) cohort, and completed an online survey followed by a semi-structured interview. Interviews were analysed using thematic analysis. Setting: Interviews were conducted over the telephone in July 2020 as the first UK lockdown was ending. Participants: 23 mothers of children with intellectual and developmental disabilities aged 5 to 15 were recruited. Results: Themes reported by parents included: managing pre-existing challenges during a time of extreme change, having mixed emotions about the benefits and difficulties that arose during the lockdown, and the need for appropriate, individualised support. Conclusions: Our findings confirm observations previously found in UK parents of children with IDD and provide new insights on the use of technology during the pandemic for schooling and healthcare, as well as the need for regular check-ins.

Article Summary

Strengths and limitations of this study:

- This study recorded the experiences of parents who were caring for children with intellectual and developmental disabilities during the first UK national lockdown.
- Short questionnaires assessed the emotional wellbeing of parents and their children quantitatively.
- 23 caregivers were interviewed in July 2020 as the first UK lockdown was ending, and were asked to reflect on their experiences across the entire lockdown.
- Interviews were conducted over the telephone and transcripts were analysed using reflective thematic analysis.
- Only mothers were interviewed and so results may not reflect the experiences of other caregivers of children with intellectual and developmental disabilities.

Keywords

Intellectual disability, developmental disabilities, covid-19, pandemics, pandemics, mental health

Click here to enter text.

For peer review only

Introduction

Intellectual and developmental disabilities (IDD) affect approximately 1.8% of children worldwide [1].They are associated with significant limitations in cognitive and adaptive skills [2]. Genetic variation can be identified in up to 60% of IDD cases [3]. These changes can be large chromosomal abnormalities (such as aneuploidies and translocations), sub-microscopic deletions or duplications (such as copy number variants; CNV) or small changes in DNA sequence (such as single nucleotide variants; SNV).

The IMAGINE-ID consortium has recruited children with IDD of identified genetic aetiology from across the UK. The coronavirus (Covid-19) pandemic poses unique challenges to this cohort in two respects. Firstly, children with IDD have high levels of pre-existing health conditions and so may be considered especially vulnerable to the virus. As a result, families of these children are more likely than the general population to have been shielding, following UK government advice. Secondly, children with IDD are more likely to make use of services (such as specialist education, healthcare, and social care) which were suspended or diminished during the pandemic [4–8]. Calls have been made to prioritise the collection of high-quality data on the mental health effects of the pandemic, particularly within vulnerable groups [9,10].

In “normal times” children with IDD are at increased risk of behavioural difficulties compared to typically developing children [11–16]. Families of a child with IDD are more likely to live in deprived socio-economic circumstances [17–21], and experience increased parenting stress or parental psychological distress [22–28] than typically developing children. Early reports of the impact of the pandemic on children with IDD and their parents suggest that many were feeling overwhelmed, forgotten and had experienced high levels of anxiety and depression [9,29–35].

International surveys and interviews with families of children with IDD or physical disabilities suggest that Covid-19 national lockdowns have led to increased behavioural problems for children [36], as well as reduced opportunities to socialise [4]. Parents have also reported concerns about their child’s safety, and that children may fall behind developmentally because of reduced access to services [37].

The nature and extent of Covid-19 restrictions has varied across different countries, with the UK being particularly severely impacted by infections. It is therefore important to assess the experiences of families in the UK in addition to the emerging international literature. One previous qualitative study has examined the experiences of 8 mothers of children with IDD in the UK [33]. These authors found that mothers felt under pressure to provide their child with educational and therapeutic provisions, which had been removed or reduced because of the pandemic. Mothers reported a lack of support from additional services, leading to increased stress and uncertainty, but also enjoyed the freedom from routine with several noting the positive impact it had for their child’s wellbeing.

We interviewed the caregivers of children with IDD, who have been caring for their children at home during the pandemic. The present study was carried out concurrently with those described above, and we seek to extend their useful findings in the IMAGINE-ID cohort to identify consistencies across research as well as any additional insights from the parents in our cohort.

Aims and objectives

We sought to understand the experiences of families caring for a child with IDD during the Covid-19 pandemic in order to identify key targets for support. We interviewed 23 IMAGINE-ID families mid-July 2020. At this time, the lockdown restrictions were eased in parts of the UK, but the government advised vulnerable individuals to continue to shield until the 1st of August 2020. We asked families to reflect on their experiences across the entirety of the first national lockdown. Our objectives

For peer review only - http://bmjopen.bmj.com/site/about/guidelines.xhtml

were: (1) to identify the areas of difficulty and resilience during the pandemic and de-confinement; (2) to identify key targets for support.

Methods

Study Design

The IMAGINE-ID programme of research, funded by the UK Medical Research Council (MRC) from 2014 to 2024, recruited a cohort of 3,402 UK families whose child had IDD due to an identified genetic anomaly (www.imagine-id.org). IMAGINE-ID caregivers with children under the age of 16 were invited to take part through the study newsletter. Parents were enrolled in the current study through consecutive sampling due to the time restrictions, so that all participants were interviewed prior to end of shielding in the first UK lockdown. A mixed-methods approach was used to capture the diversity of challenges encountered and resilience factors drawn upon by families. Data were collected by parent report using online questionnaires and a semi-structured telephone interview (n=23). Questionnaire data were collected and managed using REDCap electronic data capture tools hosted at University College London [38]. Telephone interviews were recorded and transcribed by the research team. Ethical approval for the study was obtained from UCL Research Ethics Committee (9553/003), and all participants consented to take part and for their interviews to be recorded.

Patient and Public Involvement

The research objectives were developed in response to conversations with collaborators at the UNIQUE rare chromosome disorder charity. A summary of the results will be disseminated to participants and the IMAGINE-ID cohort via the study newsletter, website and social media accounts.

Measures

Coronavirus Impact: An adapted version of the National Institute of Health CoRonavIruS Health Impact Survey (CRISIS) was used to assess the family's adaptations to the pandemic, including key domains relevant to mental distress and resilience [39].

Child Characteristics: Demographic details and genetic data about the child were extracted from the IMAGINE-ID database. The Wessex Scales were used to gain an updated measure of adaptive function and degree of IDD [40]. The measure assesses self-help skills, literacy, mobility and incontinence.

Wellbeing: The wellbeing of the child and the parent were assessed using the Strengths and Difficulties Questionnaire (SDQ) and the Hospital Anxiety and Depression scale (HADS) respectively The SDQ is a well-validated behavioural screening questionnaire for children and has been validated with the parents of children with disabilities [41]. The SDQ includes scales that measure emotional symptoms, conduct problems, hyperactivity/impulsivity and inattention difficulties, peer relationship problems and prosocial behaviour. The first four scales are combined to create a total difficulties score. On the total difficulties scale scores of 17-19 are indicative of "high" levels of behavioural difficulty and 20-40 are considered "very high" levels of behavioural difficulty compared to population norms. An additional impact scale measures the impact of this composite score on daily life. Scores of 3-10 on the impact scale are in the "very high" range compared to population samples. The HADS was employed as a self-report measure of parental mental health [42]. It has been validated for use with the parents of children with disabilities [43]. It includes a total of fourteen items, with seven depression items (e.g., 'I feel as if I am slowed down') and seven anxiety items (e.g.,' I get sudden feelings of panic'). For the anxiety and depression scales scores in the 0-7 range are considered "normal", 8-10 "borderline and scores of 11-21 "abnormal"

Interview: The semi-structured interview schedule was developed to examine the experiences of parenting a child with additional needs during the pandemic. It aimed to identify the unique

For peer review only - http://bmjopen.bmj.com/site/about/guidelines.xhtml

challenges experienced by families as well as resilience factors. Questions invited caregivers to reflect on their experiences of the lockdown, including how their day to day life had changed, how they'd coped and identifying coping strategies they had implemented. Parents were asked about their experiences across the UK lockdown, including reflecting back on their experiences during the period of stricter restrictions, as well as the easing of restrictions taking place at the time of the interview (see Supplemental materials for the exact questions asked). Interviews ranged from 13 minutes to 1 hour and 25 minutes, with a mean length of 44 minutes, and all interviews were conducted by JW and LH.

Analysis

Questionnaire data were summarised using descriptive statistics. De-identified transcripts of interviews were analysed using reflective thematic analysis as described by Braun and Clarke [44–46]. Analysis was led by JW and LH. Adopting a critical realist framework, analyses involved identifying both semantic and latent meanings in the dataset following an inductive approach, whereby themes were generated in response to interview data, rather than trying to accommodate data within predefined themes or research questions [45]. The lead authors (JW and LH) are research psychologists, whose theoretical stance is grounded in a social model of disability. JW's research background is focused on understanding the impact of rare genetic disorders on the neurodevelopment of children with rare genetic disorders. LH's expertise is in camouflaging in autism, using qualitative and quantitative methods to explore characteristics related to identity, mental health, and support. None of the authors have IDD themselves, but there is personal and professional experience of IDD amongst authors.

JW and LH proceeded through the stages of data familiarisation, coding, theme development and review. Themes were reviewed following several detailed discussions with the other members of the research team DS and WM. Following these discussions, the final themes and sub-themes were confirmed. In a final stage, the full transcripts were coded by independent reviewers LW and TA following a codebook to ensure consistency.

Results

Situating the sample

23 parents took part in interviews. All the respondents were the mothers of IMAGINE-ID children; five were single parents. They reported on 14 boys and 9 girls aged 5 to 15 years old (M=9, SD=2.9, Table 1). 78% of children received extra help at school or attended a special educational needs school. Most of the children were reported to be of white British ethnicity (n=19), other ethnicities included one mixed white and black child, one Irish child and one Asian child. The genetic make-up of the children was varied and included three children with sex chromosome aneuploidies, 16 with a CNV and four with an SNV (Table S1). All but three of the families included siblings. The Wessex scales indicated that the group included one non-verbal child. Two children were non-ambulant, 15 were partly mobile and the remainder were fully mobile. Seven children were not fully continent and only three children were reported to be literate. None had severe hearing or sight impairments.

On the SDQ 39% of children were rated to have behavioural difficulties in the "high -very high" severity bands. In 91% of children the behavioural difficulties were rated to have a "very high" impact on the family's day to day life. 35% percent of children had been diagnosed with an ASD and five had a diagnosis of Attention Deficit Disorder or Attention Deficit/Hyperactivity Disorder.

When rating themselves on the HADS for anxiety symptoms 17% of parents scored in the borderline range and 26% scored in the abnormal range. For depressive symptoms 22% scored in the borderline range and 4% in the abnormal range.

For peer review only - http://bmjopen.bmj.com/site/about/guidelines.xhtml

Coronavirus impact: Two families suspected that their household had been exposed to someone likely to have Covid-19, but only one household reported having a member of the household test positive for Covid-19. Two families reported non-household family member deaths due to Covid-19. At the time of interviews none had a confirmed case in their immediate family.

Adapting to the restrictions: A quarter of families were shielding due to concerns about vulnerability to the virus. 83% reported that their child had had some difficulty in following the recommendations for keeping away from close contact with people and 87% reported that the restrictions on leaving home had been stressful for their child. 61% of children's schools had been closed.

Financial stability: The pandemic had a financial impact on families, with 39% of respondents reporting that the pandemic had reduced their ability to earn money.

Emotional impact on child: 65% of children were reported to be worried about becoming infected and 35% were concerned about their physical health. Overall, the children were reported to be more worried about friends and family becoming infected by Covid-19 than themselves.

The pandemic led to a few unexpected positives, with 74% of families reporting that the pandemic had led to some positive changes in their child's life. These will be described in more detail in the qualitative results.

[Table 1]

Thematic Analysis

Three main themes were identified, representing a range of experiences across different families. Overall, parents described the difficulty of adjusting to the pandemic with the additional challenge of their child's specific needs ("Managing pre-existing challenges"); they described unexpected benefits and unanticipated challenges ("Mixed emotions"), and they emphasised that personalised support was essential for their families to get through the lockdown ("Support matters"; Table 2).

[Table 2]

Theme 1. Managing pre-existing challenges, in a time of increased strain for everyone "you just feel like our life was so different to other people's"

Parents described the unique daily challenges they faced before the pandemic, emphasizing how different their lives were to those of families without children with special needs. Some families were surprised to find that some of the challenges brought on by the lockdown, were familiar.

Sub-theme 1.1 "We've been in lockdown since he was born" Social distancing is the norm

Many families described that social distancing had been part of their daily life before the pandemic. Some aspects of social distancing, such as going to public places outside of peak hours, avoiding busy social settings and monitoring crowds, felt normal:

"We've been in lockdown since he was born. When we go on holiday we have to think about how busy it's going to be. We have to think about how many people are going to be there, if we're going to a restaurant; is it too noisy? Are there too many people? Are people too close to us? So we've always got social distancing in our heads anyway and we always have done. I wish other people could see (..) So that people with normal families could read it and think we're all going back to our normal life but these families aren't. It's definitely a different world that we live in." (P23)

For some, the social distancing measures did not have an impact on their social life, as they already had limited opportunities to socialise:

Click here to enter text.

For peer review only - http://bmjopen.bmj.com/site/about/guidelines.xhtml

"To be honest for me it's not all that different than usual because I don't really see a lot of people anyway" (P4)

But for many it increased their sense of isolation:

"I think you just feel more isolated than you would normally, and it was always a bit like that on summer holiday. So if I go out and see all of the kids off and they're doing things that we could never really ever do, but it was just kind of that times 100, that feeling." (P12)

Sub-theme 1.2 Left behind: "I feel like they just left people who are vulnerable behind for two months."

Many parents described feeling left behind by a range of services and felt that their child's wellbeing was considered less important because of their additional needs:

"I just felt like he was forgotten and left to it, like his education doesn't matter because he has special needs". (P12)

"Like just the support and stuff, obviously for children with special needs hasn't been there." (P14)

Furthermore, many parents believed their needs had not been prioritised. They felt let down in comparison to the support that had been offered to other families:

"I think there is definitely something to say for the fact that our community hasn't been mentioned and how have they been supported. Because I certainly haven't had much." (P20)

Sub-theme 1.3 Planning for complex needs "there's a lot more things that I needed to do than the average sort of family"

Despite some aspects of lockdown being familiar, many felt the lockdown restrictions had created a new set of challenges to be navigated. Parents described having to manage an increase in behavioural problems, uncertainties in co-ordinating medical care, and new worries about explaining covid.

Fewer resources, but behavioural issues are the same

Managing behavioural difficulties and challenging behaviour was not uncommon for families prior to the pandemic. However, parents described the strain of managing these existing behavioural concerns with fewer resources, such as respite care, school, social care and their support networks. Some highlighted an increase in intensity and frequency of behavioural outbursts:

"Meltdowns, swearing, shouting, taking his frustrations out on us. On the furniture, chairs being knocked over, doors being slammed. So rather than being able to talk to us and say 'I don't like what's happening,' it came out in meltdowns and fairly intense." (P18)

In some cases, this contributed to the escalation of pre-existing behavioural outbursts and mental health problems, to the point of serious concern.

"I think the main thing that got me, and affected me the most was [child 1] saying, when he got so frustrated that he just wanted to kill himself." (P14)

Some parents noted the re-appearance of challenging behaviours that had previously been well managed:

"Yeah he gets very hyperactive, very loud we get a lot throwing. We were working with a child psychologist at school for about 5 years on a lot of his behaviours and a lot that we nipped in the bud and improved have come back definitely in the initial stage of the lockdown." (P10)

For peer review only - http://bmjopen.bmj.com/site/about/guidelines.xhtml

Changes in routine were the source of anxiety and behavioural outbursts for many children:

"His normal life completely changed. So the anxiety was more about 'I'm being told I can't do the things I would normally do.' And that then causes anxiety because he still hasn't got control over what he would normally do. (…) He needed to have some routine or some structure around his day." (P18)

Explaining Covid, changing rules and following rules: "How do you explain a pandemic?"

The level of understanding of the pandemic in the children varied substantially. Most had some awareness of the virus and the restriction measures, but some were unaware of the pandemic completely.

"I don't think he really understood at all, other than that he wasn't going to school and he wasn't doing what he normally does, but he really didn't know why. Even though on some levels his understanding is quite good and we can explain things to him. I mean how do you explain a pandemic?" (P10)

"I think he understands. I asked him yesterday how far two metres is: 'am I two metres away from you?' and he said 'no'. So he's got that sort of understanding." (P17)

Some families found using visual aids and social stories helpful to explain the effects of the pandemic to their child, but there were limits to how much could be conveyed in a simplified way:

"We asked for social stories, so he had a social story about washing his hands, about distancing, the fact that there was going to be a one-way system around the school. So by having that, we kind of prepared him for those things." (P18)

Many children had some difficulty applying the social distancing measures to friends and family. This meant that some parents modified their behaviour and avoided certain situations to keep their children safe.

"We've been doing shopping trips without him, but when we have sort of bumped into friends when we've been out and about he doesn't quite understand. We have to make sure to keep hold of him because he wants to go and give auntie a cuddle and he doesn't quite understand that he can't do that" (P10)

Some children were reported to understand the rules and were keen to follow them. Some of these children found it difficult to understand why other people were not following the rules in public places, and at times attempted to police strangers about their rule-breaking behaviour:

"She's very good at rules. She's very interested in the Boris Johnson announcements and she takes them very literally. So if she sees somebody in the street not obeying social distancing law she won't think twice about telling them that: 'you're not social distancing and you need to be this far away'." (P22)

Theme 2. Mixed emotions around the challenges and unexpected benefits of lockdown: "The pandemic was nice but really hard"

When reflecting on the overall experience of the pandemic, and particularly the lockdown, most parents had mixed emotions. Although there were many challenging moments, for many families there were also unexpected benefits which parents wanted to extend beyond the pandemic.

Sub-theme 2.1 Desperate for hugs: "I don't like lockdown because I want snuggles with my nanny"

For peer review only - http://bmjopen.bmj.com/site/about/guidelines.xhtml

For peer review only

One common report from parents was that children were strongly impacted by not being able to see friends or extended family. Children missed both physical and emotional contact with others, and communicated that to their parents.

"He talks about the school and people he misses, so I know he does that when he misses friends or family – he mentions their name or with a question mark at the end quite clearly in his voice, that he's missing them and wants to see them." (P20)

Some children were also frustrated by not being able to take part in activities they normally enjoyed, whether because the activities were cancelled, or because the family was shielding.

"He's a nature boy he likes to go outside of the house, he loves going to places where there are animals or somewhere with a stream you know or that sort of thing, so he's sort of in his element there, so I think he's definitely missing out" (P2)

Sub-theme 2.2 Happy at home: "Everyone's keeping distance from me and that's how I like it"

However, quite a few parents reported that their child with IDD was happy being at home during the lockdown. Many children felt that home was a safe and comforting place, where they could follow their own preferred routines and didn't have to worry about the anxieties of school or the outside world.

"She loved being in, she'd rather be at home any day. She'd shut herself away and she plays on her own a lot, so yeah it really was good for her. She loved it, she was well happy with it." (P7)

"It's been great, [child] doesn't like other people, he doesn't like crowds and he doesn't want to be interacting with other people so the government saying to other people you can't interact with people I think was great, we enjoyed it." (P23)

A few parents suggested that the lockdown had actually improved their child's mental health:

"He is much happier, much much happier during the pandemic. I wasn't worried about that side of things, he's happier in himself, he's more content, more confident, more self-belief in who he is as a person. He's got more resilient coming out of it." (P1)

However, some parents felt that their children's life skills and social skills had regressed due to the lack of contact with other children, and were concerned about the impact this was having.

"He's become a lot more withdrawn, I've seen quite a lot of regression in himself, his social skills." (P2)

Sub-theme 2.3 Strained relationships: "Being constantly 24/7 together definitely did build up pressure"

For some families, staying physically safe had meant staying at home. Spending all their time together had put more pressure on relationships within and between parents and children:

"A lot of shouting, particularly with a hormonal 15-year-old and a dad who's quite stressed, even though he's working from home he's probably doing longer hours than he was in the office, so it's been sort of little things that've built up and then there's been explosions" (P10)

Strategies that parents would normally use to de-escalate conflict, such as giving children time by themselves, were no longer possible when families couldn't leave the house for weeks at a time.

"The relationship between him and his brother has been very difficult during lockdown because we've not been able to give them their space from each other, so it's not been easy" (P19)

For peer review only - http://bmjopen.bmj.com/site/about/guidelines.xhtml

As the lockdown continued for several months (and for longer than many initially expected), many parents felt like they were unable to have a break and it took its toll on the parents' wellbeing:

"Now we're just on survival" (P19)

Sub-theme 2.4 Spending time together and slowing down: "It brought us a lot closer together"

The main positive experience of lockdown, which was reported by the majority of parents, was the opportunity to spend more time together as a family as everyday life became less busy. Parents described having more time to be able to cook, eat, and play together. Families felt closer, and enjoyed their stronger relationships – something they wanted to continue after the lockdown finished.

"In terms of our relationship, it's had a positive effect because we're eating more family meals together and we're just generally spending more time together" (P13)

Being able to take things more slowly had a positive impact on wellbeing for both parents and children, as they felt freed from the daily rush. A break from rushing between different medical or school appointments was appreciated, with some parents noting that it was worth the switch to online services as they no longer had to take the time and energy to travel often significant distances for their child's appointments.

"Once we got in the swing of that, it was actually miles better because you didn't have to rush out for the bus, you know you're not working, you're not racing around trying to sort everything out. You can do everything at your own pace." (P9)

Theme 3. Support matters

One factor which strongly contributed to the family's overall experience during the lockdown was the support provided by external sources. This includes educational support from schools, either for home-schooling or providing in-person schooling where this took place, as well as support for their child's medical and psychological needs. The amount and type of support provided by these services varied significantly, with some parents reporting regular contact and others no contact at all.

Sub-theme 3.1 Transition to telehealth "I don't think you can replace face to face with a telephone"

Most children had complex behavioural and medical needs, which require regular clinical monitoring. 91% of families described the cancellation or postponement of routine medical and social care appointments.

"It's been really bad. All the appointments for the children have been cancelled. I've literally got a list on my fridge of about 15 appointments that I have to chase up, and get re-booked in once they can do so" (P14)

Most healthcare services started using tele-medicine during the pandemic. Parents described varied experiences of tele-health. Some enjoyed the experience and were grateful that their child's appointments could still go ahead:

"They were quite good, they said they're not seeing anybody but they did again do it virtually. So they did a phone call and then I emailed pictures and stuff, which is quite funny trying to do that! But at least kind of that was, they're still trying to do it." (P12)

Click here to enter text.

For peer review only - http://bmjopen.bmj.com/site/about/guidelines.xhtml

Many parents described needing specialist face to face care due to the complex nature of their child's needs and abilities. In some cases it was challenging to communicate the complexity of their child's needs remotely.

"How can you explain to the physiotherapist how she walks over the phone? It's no good doing Zoom because obviously you still can't show, it's difficult so basically she's had to go off what I say and I've had to explain to her you know into detail and she done a report from there so that's difficult." (P16)

For others, the child was not able to communicate over the phone with professionals, and so was not able to access the support that was offered.

"Yeah well we had a CAMHS [Child and Adolescent Mental Health Service] meeting but it was over Zoom and I just phoned them and I cancelled it, I said there's not a hope in hell you're going to get him to talk to you over a phone, he won't speak to anybody on the phone he doesn't, he can't get that there's somebody on the other side of something" (P23)

Sub-theme 3.2 Considering equality and equity in remote schooling

Equality through access to technology: "I think the first thing to do is make sure everyone is able to access what they're providing"

Approximately half of children's schools remained open throughout the lockdown. Most children had at least a few weeks of home-schooling, and while some returned to in-person schooling partially or fully, other children remained at home until the lockdown ended in July. During the home-schooling period, schools made various attempts to adapt the educational experience to support parents and children, particularly supporting learning at home through online educational resources or teaching. Some parents reported that their school system provided them with resources to engage with online learning, which were appreciated.

"Anybody who was vulnerable or SEN got offered a tablet or a laptop. And most people took up on that. [Child] got his own laptop from school and that made a world of difference" (P8)

However, other families were not offered these same resources, or the resources they were given were not appropriate or accessible. This often made it harder to engage with online learning.

"But everyone is in the same boat, and some children have to work on their phones because not everybody's got laptops, have they? And even doing on a tablet is not easy. So doing like math is okay on a tablet because you're just typing numbers but if you're like writing a longer piece of writing or a history essay on a phone or a tablet, that's a nightmare." (P9)

Equity of access lagging behind as adaptations aren't always appropriate

However, many parents said that their child's level of intellectual disability, or other needs, meant that these resources were simply not accessible, or required constant supervision from a parent.

"They developed a really fantastic online programme, but for the kids who have special needs that's very difficult to access." (P11)

Many parents felt that the level of work sent home was either too demanding, or not challenging enough for their child. Children with IDD who were in mainstream education were often sent the same work as their typically developing peers, and parents were offered little help in adapting this for their child's abilities, often having to rely on material that was inappropriate for their child's age.

For peer review only - http://bmjopen.bmj.com/site/about/guidelines.xhtml

"I think the biggest thing it would help it's special needs resources that aren't too babyish or patronising you know I'm getting work for him that meets his ability level academically but it's boring you know it's duckies on a pond and things like that" (P17)

Sub-theme 3 Checking in: "just being able to have that support bubble, rather than being locked in your own four walls"

In general, parents felt the most supported when any support was targeted to the child's individual needs, and when services were 'checking in' with parents, rather than waiting for problems to arise. Parents appreciated social and emotional support as much as, if not more than, practical support. This could come from individuals within services, or from friends and family, and was a way to let off steam when family life was difficult.

"I mean the other main thing to cope is – regularly calling up family or friends and having that contact. Because I'm with all the children I needed a break. So having that time to have a ten minute chat, to call, catch up. Tell someone how you're doing: 'I'm having a stressful day mum, how are you?' and those kind of things. This just made things easier. I think just talking to someone on the phone, an adult, as opposed to children" (P21)

Parents appreciated getting support from Special Educational Needs services in school, and felt better able to continue their child's education at home because they were able to ask questions and update the child's teachers on the amount of progress being made.

"We've had the SENCO [Special Educational Needs Co-ordinator] ringing us every three days checking in. She's also under the learning support service, that the school puts by for them and they support [child 1] for maths, but they've been ringing me every week. The head teacher has even phoned a couple of times. I know from speaking to people that that's not the norm, but we've felt very very much supported and I've found that I've been quite honest with them as well to be honest." (P22)

When parents did feel supported by medical and social services, this was usually the result of individuals reaching out, rather than a system-wide approach to support.

"'I've also got a caseworker. And she's been fantastic. She's been there if you needed her, she's dropped masks off to me. We've had hand sanitisers dropped off. So she's been an absolute gem. If I ever needed anything, again shopping, she would be more than happy to have done it. So I've had her support well so I've been quite lucky on that" (P6)

Discussion

In addition to the challenges experienced by neurotypical families during the pandemic, parents of children with IDD have also had to manage the distinct challenges related to their children's complex needs. Overall there was extensive variation in parents' reported experiences; some of this variation is likely to reflect the wide range of needs of children in the IMAGINE-ID cohort. Although all children in the cohort have IDD, some children attended mainstream schools and had relatively low levels of support needs, whereas others had multiple physical and behavioural needs.

Parental distress Overall, the parents displayed great resilience, but this often came at the detriment of their own mental health and wellbeing. In line with previous research, we found that caregivers had experienced increased levels of distress [9,29–31,33]. Where children needed high levels of personal care, parents often felt overwhelmed and unsupported, unable to access the respite care or specialised support they normally received. Many described high levels of distress and being close to burn-out, as confirmed by high levels of anxiety in the questionnaire. Evidence from other research also suggests that during the pandemic the parents of children with IDD

Click here to enter text.

reported higher levels of burnout than the parents of children without disabilities [47] and that the burden of care demands placed carers under increased strain [48].

The findings also confirm previous reports that service provision for children with IDD had reduced or stopped during the pandemic [4,5]. Similar findings were observed in our study, with most parents reporting at least some cancelled medical or social appointments. Social support for the parents in our study was often limited, particularly for lone parents. Many parents felt that online and telephone communication with friends and family did not provide enough support, and several felt isolated as their experience of parents of children with IDD was very different to that of other parents. Parents who reported feeling supported appeared to experience less distress. In a larger quantitative study, Willner and colleagues [31] reported that the parents of people with an IDD received less support than parents of those without IDD, with those caring for people with severe challenging behaviour receiving the least support. This suggests that families who might have benefited most from support were the least likely to receive it.

Short but regular check-ins Regular check-ins with services and schools were described as very helpful, a novel insight that has not, to our knowledge, been reported in previous research. Parents who reported more positive experiences with online schooling tended to report frequent, individualised contact such as phone calls and emails with their children's teachers and support staff, which helped the parent adapt schoolwork for their child. A five-minute telephone call with each family once a week may be more effective than two hours working with the entire class, for children with special educational needs. Even when appointments were cancelled or moved online, frequent communication with services meant parents felt supported during this time of uncertainty. Frequent, brief check-ins are a cheap and easy way to maintain the support available to families during times of crisis, and may reduce the risk of ongoing concerns developing into significant problems.

Unexpected positives Most families were able to draw some positives from the pandemic, as they were able to enjoy spending more time together, as has been previously reported [4,9,33]. Parents also appreciated stepping back from the rush of meetings and appointments, again suggesting that regular check-ins (which might be remote) might suit some families more than having to attend appointments in person.

Impact on children The impact of the pandemic on children's behaviour varied substantially. Some parents reported an increase in behavioural outbursts, while others explained that spending more time at home had led to a decrease in stress due to less demands on social interaction. A study by Neece and colleagues [4] found that "the parents of children with mental and physical disabilities were more likely to report changes in their child's behaviour, such as distractibility, inability to concentrate, irritability and general discomfort." Similar findings were observed in the present study, with several parents reporting that behavioural challenges their children had previously outgrown re-emerged because of changes in routines and lack of support.

Many children were reported to benefit from routines and consistency in their daily lives; these were often disrupted at the start of the lockdown as schools and services closed down and many families started shielding. Where parents were able to introduce new routines, this seemed to benefit children, particularly when these routines allowed children more time at home doing activities they enjoyed. Establishing new routines may be a predictor of positive adjustment in this group. The need for routine has been identified in other research into the impact of the pandemic; lack of routine and or structured support has been associated with frustration for children, as reported by parents [4,33].

For peer review only - http://bmjopen.bmj.com/site/about/guidelines.xhtml

Hardware is a starting point, not a solution Parents highlighted an important difference between the equality and equity of access to digital services. They were often given equality of access to technology, as school provided most families with laptops during the homeschooling period. However, the equity of access often lagged behind, as the resources made available to them in a digital format were not sufficiently tailored to allow their children to engage with them. This compliments the observation by Neece and colleagues [4], that some parents felt ill-equipped to support their child's online learning. Without adaptations, digital homeschooling has the potential to increase inequalities. An individualised approach is essential to understand what is needed, and the best ways to provide support.

Tele-medicine as complementary care delivery Tele-medicine is unlikely to be suitable for all IDD families. In the early stages of the pandemic, many medical and social services closed completely. However, by the time the interviews took place, some were starting to introduce tele-health services to keep in contact with the families in this study. Families enjoyed the flexibility that tele-medicine gave them, reducing the need to travel to appointments with children. However, there was a real sense that tele-health was not an appropriate substitute for in-person assessments, particular for children with IDD. Parents felt that they were not able to communicate their children's often complex needs over the phone or email, and the children themselves often struggled to engage with medical professionals who they couldn't see in front of them. These issues have been previously raised with regards to telehealth services provided during the pandemic, with the need for adaptation highlighted [49,50]. While tele-medicine offers many opportunities to expand services, it is important to emphasise that this new mode of healthcare provision will not suit everyone, and families of children with IDD should be offered a range of options to ensure their child receives appropriate support. Reflecting a broader shift to digital services across healthcare provision, blended care will likely be the most appropriate approach going forward.

Strengths, limitations and future directions

The study reflected the experiences of the mothers that were interviewed. Clearly, this small study cannot represent the full extent of the impact of the first UK lockdown on the IDD community. Our use of consecutive sampling was necessitated by the approaching end of shielding in the UK in August 2020; we felt it was important to interview all participants under the same level of restrictions. However, this also means that we were not able to selectively recruit participants, and so it is unclear to what extent variations in experience are related to variations in child's level of need, or additional factors. Our interviewees were all female, and were mostly white and in co-parenting relationships; experiences are likely to differ for non-white, non-female, single parents.

Concurrent large-scale questionnaire-based studies, such as those conducted by Willner and colleagues [31], are needed to address this. Small-scale qualitative studies can identify broader experiences than might be reported in large-scale quantitative studies, and can produce novel hypotheses for further examination. However, a key limitation of our methodology is its lack of generalisability outside of the first UK lockdown, to other countries or timepoints with different levels of infection and restrictions. We also did not interview any children or young people with IDD, and therefore our findings are reliant on parents' perceptions of their child's experience, rather than first-hand accounts. Future research should seek to explore the experiences of children with IDD through their own communication, as there are undoubtedly features which have been missed in the present study.

The interviews contained a few notable absences. We had anticipated reports of excessive hand-washing and cleanliness, however this was rarely mentioned in our interviews. We had also expected some families to express concern about being judged for needing allowances/exemptions, such as their child not wearing a mask when out in public, but this was rarely discussed. No one reported breaking the rules. Although this could be attributed to expectancy biases, we interpret

Click here to enter text.

For peer review only - http://bmjopen.bmj.com/site/about/guidelines.xhtml

this to be representative of the high national compliance during the first UK lockdown, particularly in individuals with pre-existing medical conditions [51]. Research by Rogers et al. [33] with the mothers of IDD children highlighted themes of stigma and powerlessness, which were not as prominent in our interviews.

The views presented here reflect families' experiences in the first few months of the pandemic and the first UK lockdown, a time of particularly heightened anxieties and stringent restrictions. At the time of writing the manuscript the government's response to the pandemic continues to evolve monthly. It is likely that parents' experiences and attitudes have changed over time. Therefore, we plan to conduct follow-up interviews in the spring of 2021, which may offer different insights as families have had more time to adapt to the 'new normal'. This will also allow us to explore concerns about how children with IDD have navigated the changes in rules [35-25] and explore the impact of a prolonged national crisis on the carers of children with IDD.

Conclusion

Our findings confirm observations previously found in UK parents of children with IDD [33] and provide new insights on the use of technology during the pandemic for schooling and healthcare, as well as the need for regular check-ins. Parents of children with IDD experienced many challenges during the UK Covid-19 outbreak during the spring/summer of 2020. In addition to the demands of home-schooling, concern over infection, and lack of social connection that families across the country dealt with, families in this study had to meet the additional and often complex educational and medical needs of their children. Many parents felt let down and forgotten by medical and school services, and felt isolated from the rest of the country. However, parents also used their experience to develop coping strategies and found some unexpected benefits for their children, such as spending more time together at home. We can learn from these parents' experiences to provide guidelines for the future, to ensure these families are not forgotten.

Funding statement: The IMAGINE-ID study is funded was funded by the Medical Research Council and Medical Research Foundation (MR/L011166/1 and MR/N022572/1). Research conducted at the CRF UCL GOS ICH is supported by the NIHR. The funders played no role in the design of the study and collection, analysis, interpretation of data or writing of the manuscript.

Competing interests statement: The authors declare that they have no competing interests.

Data availability statement: The datasets generated and/or analysed during the current study are not publicly available in order to protect the anonymity of those taking part, but are available from the corresponding author on reasonable request.

Author statement: JW, LH and DS designed and directed the study. JW and LH conducted the interviews and analysis (theme development and review). Themes were reviewed following several detailed discussions with the other members of the research team DS and WM. Transcripts were coded by independent reviewers LW and TA. The IMAGINE-ID consortium contributed to recruitment and manuscript revisions (see Supplemental materials).

Acknowledgements: We would like to thank all the families who took part in this study and the IMAGINE-ID consortium for making this research possible.

Word count 7,477 (excluding boxes, tables, figures and references)

For peer review only - http://bmjopen.bmj.com/site/about/guidelines.xhtml

For peer review only

References

1 Maulik PK, Mascarenhas MN, Mathers CD, *et al.* Prevalence of intellectual disability: A meta-analysis of population-based studies. Res. Dev. Disabil. 2011;**32**:419–36. doi:10.1016/j.ridd.2010.12.018

2 American Association on Intellectual and Developmental Disabilities. *Intellectual disability : definition, classification, and systems of supports*. American Association on Intellectual and Developmental Disabilities 2010.

3 Gilissen C, Hehir-Kwa JY, Thung DT, *et al.* Genome sequencing identifies major causes of severe intellectual disability. *Nature* 2014;**511**:344–7. doi:10.1038/nature13394

4 Neece C, McIntyre LL, Fenning R. Examining the impact of COVID-19 in ethnically diverse families with young children with intellectual and developmental disabilities. *J Intellect Disabil Res* 2020;**64**:739–49. doi:10.1111/jir.12769

5 Eshraghi AA, Li C, Alessandri M, *et al.* COVID-19: overcoming the challenges faced by individuals with autism and their families. *The Lancet Psychiatry* 2020;**7**:481–3. doi:https://doi.org/10.1016/S2215-0366(20)30197-8

6 National Autistic Society. The impact of coronavirus on autistic people and their families in the UK. 2020.

7 Cullen W, Gulati G, Kelly BD. Mental health in the COVID-19 pandemic. *QJM An Int J Med* 2020;**113**:311–2. doi:10.1093/QJMED/HCAA110

8 Gulati G, Fistein E, Dunne CP, *et al.* People with Intellectual Disabilities and the COVID-19 Pandemic. *Ir J Psychol Med* 2020;:1–2. doi:10.1017/ipm.2020.66

9 Rose J, Willner P, Cooper V, *et al.* The effect on and experience of families with a member who has Intellectual and Developmental Disabilities of the COVID-19 pandemic in the UK: developing an investigation. *Int J Dev Disabil* 2020;:1–3. doi:10.1080/20473869.2020.1764257

10 Holmes EA, O'Connor RC, Perry VH, *et al.* Multidisciplinary research priorities for the COVID-19 pandemic: a call for action for mental health science. The Lancet Psychiatry. 2020;**7**:547–60. doi:10.1016/S2215-0366(20)30168-1

11 Dekker MC, Koot HM, Van Der Ende J, *et al.* Emotional and behavioral problems in children and adolescents with and without intellectual disability. *J Child Psychol Psychiatry Allied Discip* 2002;**43**:1087–98. doi:10.1111/1469-7610.00235

12 Dykens EM. Annotation: Psychopathology in Children with Intellectual Disability. *J Child Psychol Psychiatry* 2000;**41**:407–17. doi:10.1111/1469-7610.00626

13 Einfeld S, Tonge B, Turner G, *et al.* Longitudinal course of behavioural and emotional problems of young persons with Prader-Willi, Fragile X, Williams and Down syndromes. *J Intellect Dev Disabil* 1999;**24**:349–54. doi:10.1080/13668259900034111

14 Totsika V, Hastings RP. Persistent challenging behaviour in people with an intellectual disability. *Curr Opin Psychiatry* 2009;**22**:437–41. doi:10.1097/YCO.0b013e32832cd9b8

15 Tonge B, Einfeld S. The trajectory of psychiatric disorders in young people with intellectual disabilities. *Aust N Z J Psychiatry* 2000;**34**:80–4. doi:10.1046/j.1440-1614.2000.00695.x

Click here to enter text.

For peer review only

16 Tonge BJ, Einfeld SL. Psychopathology and intellectual disability: The Australian Child to Adult Longitudinal Study. In: Glidden LM, ed. . London: : Academic Press 2003. 61–91.

17 Emerson E, Hatton C. Mental health of children and adolescents with intellectual disabilities in Britain. *Br J Psychiatry* 2007;**191**:493–9. doi:10.1192/bjp.bp.107.038729

18 Emerson E, Einfeld S. Emotional and behavioural difficulties in young children with and without developmental delay: a bi-national perspective. *J Child Psychol Psychiatry* 2010;**51**:583–93. doi:10.1111/j.1469-7610.2009.02179.x

19 Emerson E, McCulloch A, Graham H, *et al.* Socioeconomic circumstances and risk of psychiatric disorders among parents of children with early cognitive delay. *Am J Intellect Dev Disabil* 2010;**115**:30–42. doi:10.1352/1944-7558-115.1.30

20 Emerson E, Shahtahmasebi S, Lancaster G, *et al.* Poverty transitions among families supporting a child with intellectual disability. *J Intellect Dev Disabil* 2010;**35**:224–34. doi:10.3109/13668250.2010.518562

21 Shahtahmasebi S, Emerson E, Berridge D, *et al.* Child disability and the dynamics of family poverty, hardship and financial strain: Evidence from the UK. *J Soc Policy* 2011;**40**:653–73. doi:10.1017/S0047279410000905

22 Mugno D, Ruta L, D'Arrigo VG, *et al.* Impairment of quality of life in parents of children and adolescents with pervasive developmental disorder. *Health Qual Life Outcomes* 2007;**5**:22. doi:10.1186/1477-7525-5-22

23 Baker BL, McIntyre LL, Blacher J, *et al.* Pre-school children with and without developmental delay: Behaviour problems and parenting stress over time. *J Intellect Disabil Res* 2003;**47**:217–30. doi:10.1046/j.1365-2788.2003.00484.x

24 Gray KM, Piccinin AM, Hofer SM, *et al.* The longitudinal relationship between behavior and emotional disturbance in young people with intellectual disability and maternal mental health. *Res Dev Disabil* 2011;**32**:1194–204. doi:10.1016/j.ridd.2010.12.044

25 Emerson E, Robertson J, Wood J. Levels of Psychological Distress Experienced by Family Carers of Children and Adolescents with Intellectual Disabilities in an Urban Conurbation. *J Appl Res Intellect Disabil* 2004;**17**:77–84. doi:10.1111/j.1360-2322.2004.00183.x

26 Olsson MB, Hwang CP. Depression in mothers and fathers of children with intellectual disability. *J Intellect Disabil Res* 2001;**45**:535–43. doi:10.1046/j.1365-2788.2001.00372.x

27 White N, Hastings RP. Social and professional support for parents of adolescents with severe intellectual disabilities. *J Appl Res Intellect Disabil* 2004;**17**:181–90. doi:10.1111/j.1468-3148.2004.00197.x

28 Marquis S, Hayes M V., McGrail K. Factors Affecting the Health of Caregivers of Children Who Have an Intellectual/Developmental Disability. *J Policy Pract Intellect Disabil* 2019;**16**:201–16. doi:10.1111/jppi.12283

29 Bellomo TR, Prasad S, Munzer T, *et al.* The impact of the COVID-19 pandemic on children with autism spectrum disorders. *J Pediatr Rehabil Med* 2020;**Preprint**:1–6. doi:10.3233/PRM-200740

30 Colizzi M, Sironi E, Antonini F, *et al.* Psychosocial and behavioral impact of COVID-19 in autism spectrum disorder: An online parent survey. *Brain Sci* 2020;**10**. doi:10.3390/brainsci10060341

31 Willner P, Rose J, Stenfert Kroese B, *et al.* Effect of the COVID-19 pandemic on the mental

For peer review only - http://bmjopen.bmj.com/site/about/guidelines.xhtml

health of carers of people with intellectual disabilities. *J Appl Res Intellect Disabil* 2020;**n/a**. doi:10.1111/jar.12811

32 Couglan S. Coronavirus: Parents of disabled children ‘cut off and ignored’. BBC News. 2020.www.bbc.co.uk/news/education-52806105. Published May 27.

33 Rogers G, Perez-Olivas G, Stenfert Kroese B, *et al.* The experiences of mothers of children and young people with intellectual disabilities during the first COVID-19 lockdown period. *J Appl Res Intellect Disabil* 2021;:1–10. doi:10.1111/jar.12884

34 Westrupp EM, Stokes MA, Fuller-Tyszkiewicz M, *et al.* Subjective wellbeing in parents during the COVID-19 pandemic in Australia. *J Psychosom Res* 2021;**145**:110482. doi:10.1016/j.jpsychores.2021.110482

35 Asbury K, Fox L, Deniz E, *et al.* How is COVID-19 Affecting the Mental Health of Children with Special Educational Needs and Disabilities and Their Families? *J Autism Dev Disord* 2020;**51**:1772–80. doi:10.1007/s10803-020-04577-2

36 Cacioppo M, Bouvier S, Bailly R, *et al.* Emerging health challenges for children with physical disabilities and their parents during the COVID-19 pandemic : The ECHO French survey. *Ann Phys Rehabil Med* 2020.

37 Grumi S, Provenzi L, Gardani A, *et al.* Rehabilitation services lockdown during the COVID-19 emergency: the mental health response of caregivers of children with neurodevelopmental disabilities. *Disabil Rehabil* 2020;**43**:27–32. doi:10.1080/09638288.2020.1842520

38 Harris PA, Taylor R, Thielke R, *et al.* Research electronic data capture (REDCap)-A metadata-driven methodology and workflow process for providing translational research informatics support. *J Biomed Inform* 2009;**42**:377–81. doi:10.1016/j.jbi.2008.08.010

39 Nikolaidis A, Paksarian D, Alexander L, *et al.* The Coronavirus Health and Impact Survey (CRISIS) reveals reproducible correlates of pandemic-related mood states across the Atlantic. *medRxiv Prepr Serv Heal Sci* Published Online First: 27 August 2020. doi:10.1101/2020.08.24.20181123

40 Kushllck A, Blunden R, Cox G. A method of rating behaviour characteristics for use in large scale surveys of mental handicap. *Psychol Med* 1973;**3**:466–78. doi:10.1017/S0033291700054271

41 Goodman A, Lamping DL, Ploubidis GB. When to Use Broader Internalising and Externalising Subscales Instead of the Hypothesised Five Subscales on the Strengths and Difficulties Questionnaire (SDQ): Data from British Parents, Teachers and Children. *J Abnorm Child Psychol* 2010;**38**:1179–91. doi:10.1007/s10802-010-9434-x

42 Zigmond AS, Snaith RP. The Hospital Anxiety and Depression Scale. *Acta Psychiatr Scand* 1983;**67**:361–70. doi:10.1111/j.1600-0447.1983.tb09716.x

43 Hastings RP. Parental stress and behaviour problems of children with developmental disability. J. Intellect. Dev. Disabil. 2002;**27**:149–60. doi:10.1080/1366825021000008657

44 Braun V, Clarke V. Using thematic analysis in psychology. *Qual Res Psychol* 2006;**3**:77–101.

45 Braun V, Clarke V. *Successful Qualitative Research: A practical Guide for Beginners*. SAGE Publications 2013.

46 Braun V, Clarke V. Reflecting on reflexive thematic analysis. *Qual Res Sport Exerc Heal* 2019;**11**:589–97. doi:10.1080/2159676X.2019.1628806

For peer review only

For peer review only - http://bmjopen.bmj.com/site/about/guidelines.xhtml

47 Fontanesi L, Marchetti D, Mazza C, *et al.* The Effect of the COVID-19 Lockdown on Parents: A Call to Adopt Urgent Measures. *Psychol Trauma Theory, Res Pract Policy* 2020;**12**:79–81. doi:10.1037/tra0000672

48 Alexander R, Ravi A, Barclay H, *et al.* Guidance for the Treatment and Management of COVID-19 Among People with Intellectual Disabilities. *J Policy Pract Intellect Disabil* 2020;**17**:256–69. doi:10.1111/jppi.12352

49 Jeste S, Hyde C, Distefano C, *et al.* Changes in access to educational and healthcare services for individuals with intellectual and developmental disabilities during COVID-19 restrictions. *J Intellect Disabil Res* 2020;**64**:825–33. doi:10.1111/jir.12776

50 Provenzi L, Grumi S, Borgatti R. Alone With the Kids: Tele-Medicine for Children With Special Healthcare Needs During COVID-19 Emergency. *Front Psychol* 2020;**11**:1–6. doi:10.3389/fpsyg.2020.02193

51 Krekel C, Swanke S, De Neve J-E, *et al.* Are Happier People More Compliant? Global Evidence From Three Large-Scale Surveys During Covid-19 Lockdowns. 2020. www.iza.org (accessed 5 Jan 2021).

For peer review only

Click here to enter text.

ID	Gender	Age	SDQ
P1	M	9	Very High
P2	M	5	Very High
P3	F	12	Close to Average
P4	F	5	Very High
P5	F	7	High
P6	F	13	Slightly Raised
P7	F	6	Very High
P8	M	13	High
P9	M	11	Close to Average
P10	M	10	Very High
P11	F	9	High
P12	M	8	Very High
P13	M	13	Close to Average
P14	M	7	Very High
P15	F	13	Very High
P16	F	10	Close to Average
P17	M	9	Very High
P18	M	15	Very High
P19	M	14	High
P20	M	12	Slightly Raised
P21	M	9	Close to Average
P22	F	9	High
P23	M	10	Very High

Table 1: Participant characteristics
M: Male; F: Female; SDQ; Strengths and Difficulties Questionnaire

Theme	Sub-theme
Managing pre-existing challenges, in a time of increased strain for everyone "you just feel like our life was so different to other people's"	"We've been in lockdown since he was born" Social distancing is the norm
	Left behind: "I feel like they just left people who are vulnerable behind for two months."

Click here to enter text.

For peer review only

	Planning for complex needs “there’s a lot more things that I needed to do than the average sort of family”<br>• Fewer resources, but behavioural issues are the same<br>• Explaining covid
Mixed emotions around the challenges and unexpected benefits of lockdown: “The pandemic was nice but really hard”	Desperate for hugs: “I don’t like lockdown because I want snuggles with my nanny”
	Happy at home: “Everyone’s keeping distance from me and that’s how I like it”
	Strained relationships: “Being constantly 24/7 together definitely did build up pressure”
	Spending time together and slowing down: “It brought us a lot closer together”
Support matters	Transition to telehealth “I don’t think you can replace face to face with a telephone”<br>Considering equality and equity in remote schooling<br>• Equality through access to technology: “I think the first thing to do is make sure everyone is able to access what they’re providing”<br>• Equity of access lagging behind as adaptations aren’t always appropriate
	Considering equality and equity in remote schooling<br>• Equality through access to technology: “I think the first thing to do is make sure everyone is able to access what they’re providing”<br>• Equity of access lagging behind as adaptations aren’t always appropriate
	Checking in: “just being able to have that support bubble, rather than being locked in your own four walls”

Table 2: Summary of themes and sub-themes

For peer review only

Supplementary file

Interview schedule

1. How do you feel the coronavirus pandemic is affecting you and your family, if at all? It may be helpful to consider your response in terms of routine, appointments, respite care, support networks, visiting family, childcare arrangements, work and related thoughts and feelings.

Prompts:

a. Can you tell me a bit more about the coronavirus pandemic has changed your and your child's:

i. Day to day life?
ii. Childcare?
iii. Access to services? Have any appointments been re-arranged, taken place online or been cancelled/postponed? How easy have you found accessing online/telephone appointments?
iv. Seeing friends and family?
v. Access to support networks?

2. How do you feel the coronavirus pandemic is affecting your ability to cope?

3. How do you feel the coronavirus pandemic is affecting your parenting skills?

4. How are you getting along as a family? / How is everyone getting along together?

5. How do you feel the physical (social) distancing and lockdown/isolation measures have affected you and your child/children during the coronavirus pandemic, if at all?
If this is affecting you, can you explain or list in what ways?
For example, consider the impact on your support network, social activities, social media and online socialising, and general contact with family, friends and services etc.

6. How do you feel that the easing of lockdown measures/restrictions are affecting you and your child/children, if at all? If this is affecting you, can you explain or list in what ways? For example, consider the impact on your support network, social activities, social media and online socialising, and general contact with family, friends and services etc.

7. In an ideal world, what resources or allowances would you need to manage lockdown/ to help your child?

8. Are there any coping strategies that you are using during this coronavirus pandemic? If so, what are these coping strategies and how have they helped you?

9. Have there been any unexpected positives during the pandemic? Is there anything from the lockdown that you would like to carry forward into everyday life for you/your child?

10. Is there anything else about your experiences during this pandemic that you would like to mention?

For peer review only - http://bmjopen.bmj.com/site/about/guidelines.xhtml

Genetic variants of participants

Variant Type	Variants
Aneuploidy	48,XXYY
Aneuploidy	45,X
CNV	chr17p11.2 Duplication
CNV	chr18q12.3 Triplication
CNV	chr3q26.33-q27.1 Deletion
Aneuploidy	48,XXYY
CNV	chr7q11.23 Deletion
CNV	chr10q22.3-q23.2 Deletion
CNV	chr5q31.3 Duplication & chrXp22.1 Deletion
CNV	chr1q21.1-q21.2 Deletion
SNV	chr20p13 Missense & chrXq25 Missense
SNV	chrXp22.12 Splice site
CNV	chr16p13.11-p12.3 Duplication & chr7p21.1 Deletion
SNV	chr2q24.3 Missense
CNV	chr5q14.3 Deletion
SNV	chrXq26.1 Missense
CNV	chr16p13.11 Deletion
CNV	chr13q33.2-q33.3 Deletion
CNV	chr18p11.32-p11.21 & chrXp22.12 Duplication
CNV	chr7p21.1 Deletion
CNV	chr15q11.1-q13.1 Mosaic duplication
CNV	chr16p13.11-p12.3 Duplication
CNV	chr16p13.11 Deletion

Table S1: Genetic variants of IMAGINE-ID children with IDD

For peer review only - http://bmjopen.bmj.com/site/about/guidelines.xhtml

STROBE Statement—checklist of items that should be included in reports of observational studies

	Item No	Recommendation	Page No
**Title and abstract**	1	(*a*) Indicate the study's design with a commonly used term in the title or the abstract	1
		(*b*) Provide in the abstract an informative and balanced summary of what was done and what was found	2
**Introduction**			
Background/rationale	2	Explain the scientific background and rationale for the investigation being reported	3
Objectives	3	State specific objectives, including any prespecified hypotheses	3-4
**Methods**			
Study design	4	Present key elements of study design early in the paper	4
Setting	5	Describe the setting, locations, and relevant dates, including periods of recruitment, exposure, follow-up, and data collection	4
Participants	6	(*a*) *Cohort study*—Give the eligibility criteria, and the sources and methods of selection of participants. Describe methods of follow-up<br>*Case-control study*—Give the eligibility criteria, and the sources and methods of case ascertainment and control selection. Give the rationale for the choice of cases and controls<br>*Cross-sectional study*—Give the eligibility criteria, and the sources and methods of selection of participants	4
		(*b*) *Cohort study*—For matched studies, give matching criteria and number of exposed and unexposed<br>*Case-control study*—For matched studies, give matching criteria and the number of controls per case	N/A
Variables	7	Clearly define all outcomes, exposures, predictors, potential confounders, and effect modifiers. Give diagnostic criteria, if applicable	4-5
Data sources/ measurement	8*	For each variable of interest, give sources of data and details of methods of assessment (measurement). Describe comparability of assessment methods if there is more than one group	*4*
Bias	9	Describe any efforts to address potential sources of bias	5
Study size	10	Explain how the study size was arrived at	4
Quantitative variables	11	Explain how quantitative variables were handled in the analyses. If applicable, describe which groupings were chosen and why	N/A Descriptive statistics
Statistical methods	12	(*a*) Describe all statistical methods, including those used to control for confounding	N/A
		(*b*) Describe any methods used to examine subgroups and interactions	N/A
		(*c*) Explain how missing data were addressed	N/A
		(*d*) *Cohort study*—If applicable, explain how loss to follow-up was addressed<br>*Case-control study*—If applicable, explain how matching of cases and controls was addressed<br>*Cross-sectional study*—If applicable, describe analytical methods taking account of sampling strategy	N/A
		(*e*) Describe any sensitivity analyses	N/A

Continued on next page

For peer review only - http://bmjopen.bmj.com/site/about/guidelines.xhtml

**Results**			
Participants	13*	(a) Report numbers of individuals at each stage of study—eg numbers potentially eligible, examined for eligibility, confirmed eligible, included in the study, completing follow-up, and analysed	5
		(b) Give reasons for non-participation at each stage	N/A
		(c) Consider use of a flow diagram	N/A
Descriptive data	14*	(a) Give characteristics of study participants (eg demographic, clinical, social) and information on exposures and potential confounders	5
		(b) Indicate number of participants with missing data for each variable of interest	5
		(c) *Cohort study*—Summarise follow-up time (eg, average and total amount)	N/A
Outcome data	15*	*Cohort study*—Report numbers of outcome events or summary measures over time	*5-6*
		Case-control study—Report numbers in each exposure category, or summary measures of exposure	N/A
		Cross-sectional study—Report numbers of outcome events or summary measures	N/A
Main results	16	(*a*) Give unadjusted estimates and, if applicable, confounder-adjusted estimates and their precision (eg, 95% confidence interval). Make clear which confounders were adjusted for and why they were included	N/A
		(*b*) Report category boundaries when continuous variables were categorized	N/A
		(*c*) If relevant, consider translating estimates of relative risk into absolute risk for a meaningful time period	N/A
Other analyses	17	Report other analyses done—eg analyses of subgroups and interactions, and sensitivity analyses	N/A
**Discussion**			
Key results	18	Summarise key results with reference to study objectives	12
Limitations	19	Discuss limitations of the study, taking into account sources of potential bias or imprecision. Discuss both direction and magnitude of any potential bias	14-15
Interpretation	20	Give a cautious overall interpretation of results considering objectives, limitations, multiplicity of analyses, results from similar studies, and other relevant evidence	12-15
Generalisability	21	Discuss the generalisability (external validity) of the study results	14
**Other information**			
Funding	22	Give the source of funding and the role of the funders for the present study and, if applicable, for the original study on which the present article is based	15

*Give information separately for cases and controls in case-control studies and, if applicable, for exposed and unexposed groups in cohort and cross-sectional studies.

Note: An Explanation and Elaboration article discusses each checklist item and gives methodological background and published examples of transparent reporting. The STROBE checklist is best used in conjunction with this article (freely available on the Web sites of PLoS Medicine at http://www.plosmedicine.org/, Annals of Internal Medicine at http://www.annals.org/, and Epidemiology at http://www.epidem.com/). Information on the STROBE Initiative is available at www.strobe-statement.org.

For peer review only

Standards for Reporting Qualitative Research (SRQR) Table

No.	Topic	Page/line reference
S1	Title	Page 1, Line 1
S2	Abstract	Page 2, Lines 2-14
S3	Problem formulation	See entire Introduction section (Pages 3 – 4)
S4	Research question	Page 3, Line 42 – Page 4, Line 2
S5	Qualitative approach and research paradigm	Page 5, Lines 10-26
S6	Researcher characteristics and reflexivity	Page 5, Lines 15-21
S7	Context	Page 4, Lines 5-16
S8	Sampling strategy	Page 4, Lines 9-10
S9	Ethical issues	Page 4, Lines 15-16
S10	Data collection methods	See entire Methods section (pages 4 – 5)
S11	Instruments	Page 4, Line 22 – Page 5, Line 8
S12	Units of study	Page 5, Lines 30 – 19
S13	Data processing	Page 5, Lines 10 – 26
S14	Data analysis	Page 5, Lines 10 – 26
S15	Techniques to enhance trustworthiness	Page 5, Lines 10 – 26
S16	Synthesis and interpretation	See entire Results section (pages 5 – 12)
S17	Links to empirical data	See entire Results section (Pages 5 – 12)
S18	Integration with prior work, implications, transferability, and contributions to the field	See entire Discussion section (Pages 12 – 15)
S19	Limitations	Page 14, Line 26 – Page 5, Line 12
S20	Conflicts of interest	Page 15, Line 31
S21	Funding	Page 15, Lines 27 - 30

For peer review only
